# Concordance of genetic determinants of acute coronary artery disease between European and Chinese populations

**DOI:** 10.1093/gigascience/giag084

**Published:** 2026-08-11

**Authors:** Ahmed Edris, Kuang Lin, Sofia Massa, Iain Turnbull, Iona Y Millwood, Paul Sherliker, Liming Li, Jun Lv, Canqing Yu, Dianjianyi Sun, Pei Pei, Daniel Avery, Fang Liu, Anuj Goel, Hugh Watkins, Zhengming Chen, Robin G Walters, Derrick A Bennett, Robert Clarke, China Kadoorie Biobank Study Group, J Chen, Z Chen, R Clarke, R Collins, L Li, C Wang, J Lv, R Peto, R Walters, D Avery, D Bennett, R Boxall, K H Chan, Y Chen, Z Chen, J Clarke, R Clarke, H Du, A E Mohamed, H Fry, P K Im, A Iona, C Kartsonaki, H Lam, K Lin, J Liu, I Millwood, S Morris, Q Nie, A Pozarickij, P Ryder, D Schmidt, B Stevens, I Turnbull, R Walters, B Wang, L Wang, N Wright, L Yang, X Yang, P Yao, X Han, C Hou, Q Xia, C Liu, J Lv, P Pei, D Sun, C Yu, N Chen, D Liu, Z Tang, N Chen, Q Jiang, J Lan, M Li, Y Liu, F Meng, J Meng, R Pan, Y Qin, P Wang, S Wang, L Wei, L Zhou, C Dong, P Ge, X Ren, Z Li, E Mao, T Wang, H Zhang, X Zhang, J Chen, X Hu, X Wang, Z Guo, H Li, Y Li, M Weng, S Wu, S Yan, M Zou, X Zhou, Z Guo, Q Kang, Y Li, B Yu, Q Xu, L Chang, L Fan, S Feng, D Zhang, G Zhou, Y Gao, T He, P He, C Hu, H Sun, X Zhang, B Chen, Z Fu, Y Huang, H Liu, Q Xu, L Yin, H Long, X Xu, H Zhang, L Zhang, J Su, R Tao, M Wu, J Yang, J Zhou, Y Zhou, Y Hu, Y Hua, J Jin, F Liu, J Liu, Y Lu, L Ma, A Tang, J Zhang, L Cheng, R Du, R Gao, F Li, S Li, Y Liu, F Ning, Z Pang, X Sun, X Tian, S Wang, Y Zhai, H Zhang, W Hou, S Lv, J Wang, X Chen, X Wu, N Zhang, W Zhou, X Chen, J Li, J Liu, G Luo, Q Sun, X Zhong, W Gong, R Hu, H Wang, M Wang, M Yu, L Chen, Q Gu, D Pan, C Wang, K Xie, X Zhang

**Affiliations:** Clinical Trial Service Unit, Nuffield Department of Population Health, University of Oxford, Oxford, OX37LF, UK; Clinical Trial Service Unit, Nuffield Department of Population Health, University of Oxford, Oxford, OX37LF, UK; Clinical Trial Service Unit, Nuffield Department of Population Health, University of Oxford, Oxford, OX37LF, UK; Clinical Trial Service Unit, Nuffield Department of Population Health, University of Oxford, Oxford, OX37LF, UK; Clinical Trial Service Unit, Nuffield Department of Population Health, University of Oxford, Oxford, OX37LF, UK; Clinical Trial Service Unit, Nuffield Department of Population Health, University of Oxford, Oxford, OX37LF, UK; Department of Epidemiology and Biostatistics, School of Public Health, Peking University, Beijing, 100191, China; Peking University Center for Public Health and Epidemic Preparedness and Response, Beijing, 100191, China; Key Laboratory of Epidemiology of Major Diseases, Peking University, Ministry of Education, Beijing, 100191, China; Department of Epidemiology and Biostatistics, School of Public Health, Peking University, Beijing, 100191, China; Peking University Center for Public Health and Epidemic Preparedness and Response, Beijing, 100191, China; Key Laboratory of Epidemiology of Major Diseases, Peking University, Ministry of Education, Beijing, 100191, China; Department of Epidemiology and Biostatistics, School of Public Health, Peking University, Beijing, 100191, China; Peking University Center for Public Health and Epidemic Preparedness and Response, Beijing, 100191, China; Key Laboratory of Epidemiology of Major Diseases, Peking University, Ministry of Education, Beijing, 100191, China; Department of Epidemiology and Biostatistics, School of Public Health, Peking University, Beijing, 100191, China; Peking University Center for Public Health and Epidemic Preparedness and Response, Beijing, 100191, China; Key Laboratory of Epidemiology of Major Diseases, Peking University, Ministry of Education, Beijing, 100191, China; Peking University Center for Public Health and Epidemic Preparedness and Response, Beijing, 100191, China; Clinical Trial Service Unit, Nuffield Department of Population Health, University of Oxford, Oxford, OX37LF, UK; Suzhou CDC, Suzhou, 215004, Jiangsu, China; Division of Cardiovascular Medicine, Radcliffe Department of Medicine, University of Oxford, Oxford, OX3 9DU, UK; Division of Cardiovascular Medicine, Radcliffe Department of Medicine, University of Oxford, Oxford, OX3 9DU, UK; Clinical Trial Service Unit, Nuffield Department of Population Health, University of Oxford, Oxford, OX37LF, UK; Clinical Trial Service Unit, Nuffield Department of Population Health, University of Oxford, Oxford, OX37LF, UK; Clinical Trial Service Unit, Nuffield Department of Population Health, University of Oxford, Oxford, OX37LF, UK; Clinical Trial Service Unit, Nuffield Department of Population Health, University of Oxford, Oxford, OX37LF, UK

**Keywords:** genetic determinants, acute coronary artery disease, stroke, other vascular diseases, Chinese and European populations

## Abstract

**Aims:**

This study examined the concordance of associations of single nucleotide polymorphisms (SNPs) for acute coronary artery disease (CAD) discovered in Europeans with acute CAD, chronic ischaemic heart disease (IHD) or stroke in Chinese adults.

**Methods:**

In a nested case-control study of acute CAD (4,748 cases/66,227 controls) in China Kadoorie Biobank (CKB), we compared associations of 224 SNPs for acute CAD discovered in Europeans in CARDIOGRAMplusC4D (CC4D) with acute CAD in Chinese. We compared associations of a genetic score (GS-CAD) for 224 SNPs for acute CAD with chronic IHD (*n* = 9,000), heart failure (*n* = 1,229), stroke (*n* = 10,208), CVD risk factors (*n* = 70,364), and plasma proteomics (*n* = 3,904) in CKB.

**Results:**

The strength of associations of 224 CAD SNPs discovered in Europeans was moderately correlated with those for acute CAD in Chinese (*r* = 0.53), but significant associations were only replicated (*P* < 0.05) for 25 SNPs. The strength of associations with acute CAD for a 1 SD higher GS for acute CAD in Chinese was 3-fold greater than for chronic IHD or heart failure (1.22 [1.18–1.26] vs. 1.07 [1.05–1.10] vs. 1.06 [1.00–1.12], respectively) and 7-fold greater than for stroke (1.03; 1.01–1.05). Higher levels of GS-CAD were strongly associated with low-density lipoprotein cholesterol, moderately with systolic blood pressure and weakly with body mass index, but not with proteomics.

**Conclusions:**

The findings demonstrate a moderate concordance of genetic determinants for acute CAD between Europeans and Chinese that were specific for CAD and only weakly associated with chronic IHD, heart failure, or ischaemic stroke.

## Graphical Abstract

**Graphical Abstract:**
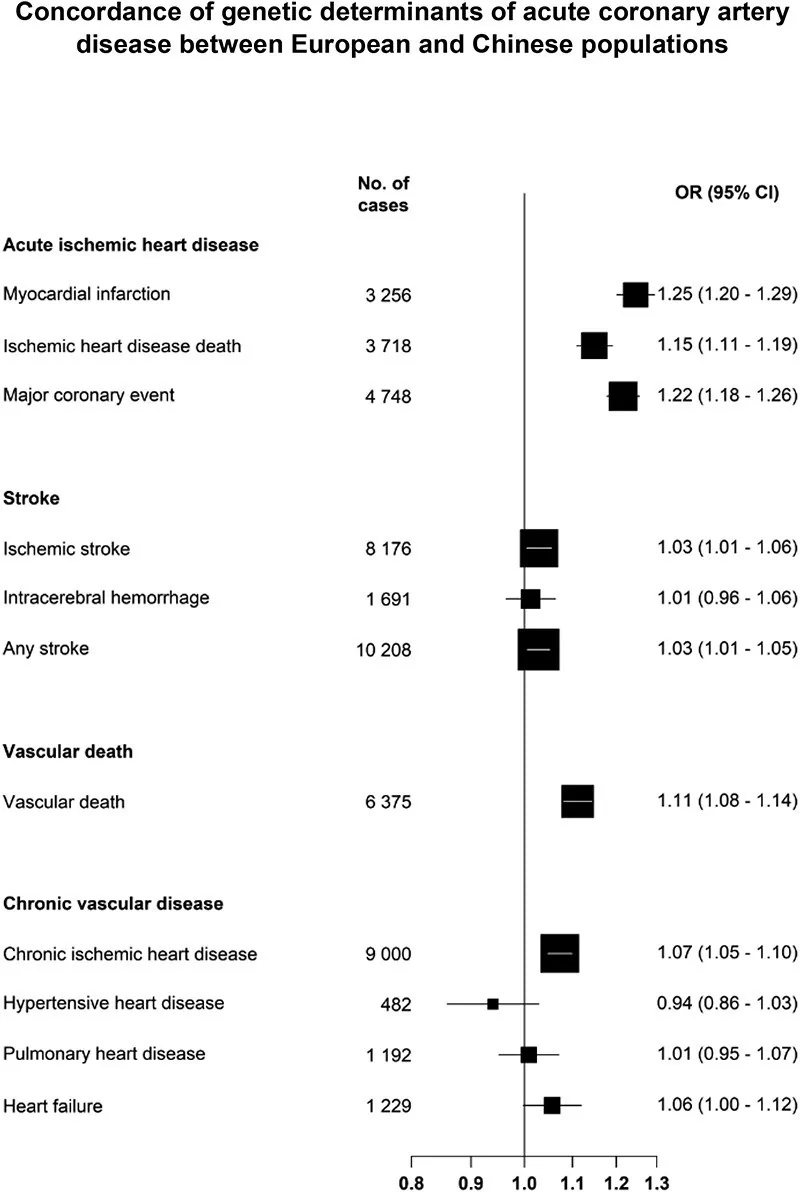

## Introduction

Coronary artery disease (CAD) and stroke are the leading causes of morbidity and mortality worldwide [1]. The worldwide prevalence of these diseases has increased almost 2-fold over the last 3 decades [2], and cardiovascular disease (CVD) mortality currently accounts for about 40% of all deaths in China [3, 4]. Premature deaths (<70 years) from CVD have declined in both Western and Chinese populations over the last few decades reflecting improvements in lifestyle and access to medical treatment to reduce levels of CVD risk factors.

The prevalence of current cigarette smoking is 4-fold higher in Chinese men than in European men (44 vs. 12%), but mean levels of low-density lipoprotein cholesterol (LDL-C) (2.5 vs. 3.0 mmol/l) or body mass index (BMI) are lower (24.5 vs. 27.5 kg/m^2^) in Chinese than in European men and women [5–8]. However, little is known about the comparative importance of genetic determinants for acute CAD (i.e., non-fatal myocardial infarction [MI] or death from ischaemic heart disease [IHD]) discovered in Europeans for acute CAD and other CVD subtypes in Chinese adults.

Genome-wide association studies (GWASs) of CVD conducted in diverse ancestry populations have greatly expanded our knowledge of risk factors for CVD, by clarifying the genetic architecture of CVD across populations, elucidation of instrumental variables for Mendelian randomization (MR) analyses to assess the relevance of such risk factors, discovery of novel targets for drug treatment of CVD and development of polygenic scores to improve risk prediction of CVD [9–16]. While meta-analyses of international consortia of GWAS studies have reported cross-ancestry differences in genetic architecture of CVD sub-types, large biobanks such as UK Biobank, Japan Biobank, or China Kadoorie Biobank (CKB) can provide additional information about differences between CVD sub-types, gene–gene or gene–environmental interactions that are not possible to discover in multi-ancestry meta-analyses of GWAS consortia of summary statistics on CVD sub-types [17–19].

GWASs of CAD in European ancestry populations have reported over 241 single nucleotide polymorphisms (SNPs) that were independently associated with CAD demonstrating a polygenic genetic architecture involving multiple SNPs in Europeans each typically having only modest effects on acute CAD [9–11]. While the effects of some of these SNPs on CAD are mediated through levels of LDL-C, systolic blood pressure (SBP), diabetes or adiposity, the mechanisms for effects of other SNPs on acute CAD are not fully understood [11–16]. Moreover, the relevance of the genetic variants for acute CAD (MI and IHD death) for other non-MI IHD subtypes (i.e., chronic IHD), non-IHD cardiac outcomes or stroke has not been widely studied in Chinese adults [10].

The different IHD subtypes vary by severity of coronary artery occlusion and reflect underlying pathology and severity of CAD. Acute CAD (involving MI or death from IHD), also referred to as “major coronary events” (MCEs), is the most serious form of acute CAD that reflects complete occlusion of one or more coronary arteries with plaque rupture and superimposed thrombosis. In contrast, the more common chronic IHD cases typically have partial occlusion of one or more coronary arteries. Acute MI is distinguished from unstable angina or chronic IHD by elevated plasma levels of troponins and characteristic ST segment changes and/or pathological Q waves on serial electrocardiogram recordings.

Comparative studies assessing the concordance of genetic determinants for acute CAD discovered in Europe for acute CAD, chronic IHD, and non-IHD cardiac diseases occurring in Chinese adults with different diet and lifestyle habits should be informative about the relevance of genetic susceptibility for acute CAD, chronic IHD, stroke and other non-IHD cardiac outcomes in China.

A recent GWAS of CAD, involving about 180,000 CAD cases, identified independent associations of 241 variants at 198 loci with CAD in Europeans [9]. Comparison of GWAS results for CAD discovered in Europeans with 30,000 CAD cases in Japanese also demonstrated consistent effect sizes for many variants, and a combined meta-analysis of CAD from European and Japanese cases yielded 38 additional variants for CAD. Previous GWAS of CAD conducted in Chinese adults identified some shared loci for CAD between Europeans and Chinese [10, 11]. Differences in the genetic architecture or frequency of common variants or effect modification by levels of established CAD risk factors could account for some differences in the incidence and severity of CAD or proportions with different IHD subtypes between Chinese and European populations [11–13]. However, there have been few comparative studies of GWAS variants for acute CAD, chronic IHD and other non-IHD cardiac outcomes in Chinese and European populations and their relevance for other CVD subtypes [14–16].

The aims of this study were to (i) compare the associations of individual SNPs for acute CAD discovered in European ancestry populations in CARDIOGRAMplusC4D with acute CAD cases in Chinese; (ii) assess the concordance of associations of acute CAD SNPs discovered in Europeans for acute CAD, chronic IHD, non-IHD cardiac conditions and stroke types using a genetic score (GS) combining such European ancestry SNPs for CAD (GS-CAD) in nested case-control studies in Chinese individuals in CKB; (iii) and compare associations of fifths of GS-CAD scores with levels of LDL-C, SBP, BMI, and plasma proteomics in controls with GWAS data in CKB [9, 17,18].

## Methods

### Study population

**Figure 1:**
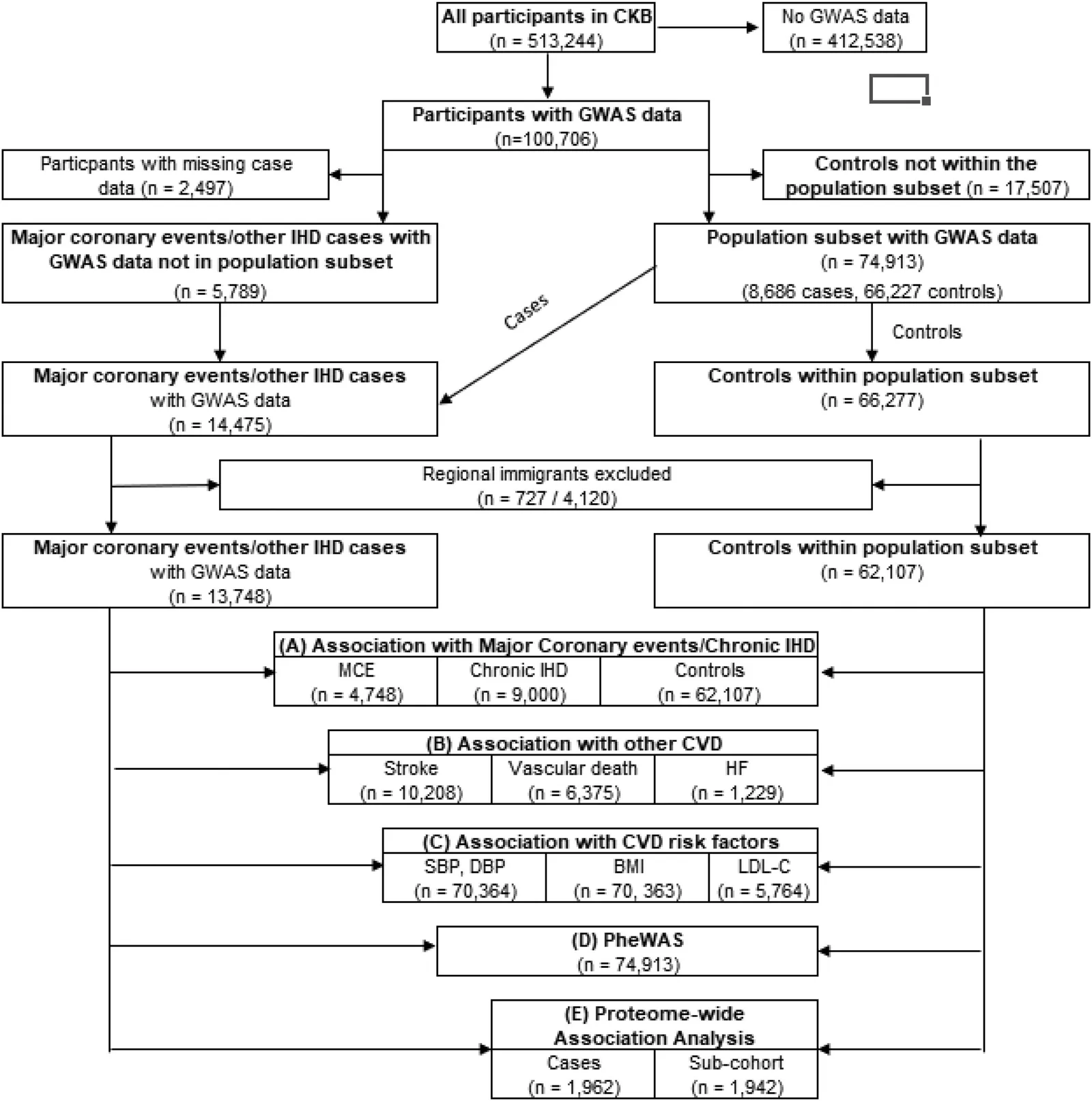
Flow diagram of study design and numbers of participants in genetic analyses of CAD SNPs with (A) MCEs and chronic IHD, (B) other CVD, (C) CVD risk factors, (D) PheWAS with 117 diseases and (E) plasma proteomics in Chinese adults.

Details of the design and methods used in the CKB study have been previously reported [18, 19]. Briefly, the CKB study involves a 12-year follow-up of a population-based prospective study of 512,891 adults aged 30–79 years who were recruited from 10 regions in China between 2004 and 2008. All participants completed an interviewer-administered questionnaire that collected data on demographic and lifestyle characteristics (including smoking and alcohol consumption), medical history, and current use of medications. GWAS data are currently available on about one-fifth of the CKB study population (constrained by available resources) involving a population subset and additional cases with MCE, stroke and other CVD subtypes and with CAD risk factors (Fig. 1). The present report involved five separate comparisons with different subsets of the CKB study population, including (A) associations of individual SNPs for MCEs that were discovered in Europeans with MCE in Chinese and a comparative study of a GS combining these SNPs (GS-CAD) with MCE or non-MI (chronic IHD) in CKB; (B) comparison of a GS-CAD score with stroke, heart failure, or vascular death in CKB; (C) comparison of a GS-CAD score with levels of major CVD risk factors; (E) phenome-wide association study of a GS-CAD score with incidence of major diseases in CKB (Fig. 1).

All participants underwent a physical examination that recorded measurements of anthropometry, blood pressure (BP), and spirometry, and provided a blood sample. Ethics approval was obtained from both the University of Oxford (Oxford Tropical Research Ethics Committee), the Chinese Academy of Medical Sciences, Peking University, and the China National Centers for Disease Control. In addition, approvals were obtained from institutional research boards at the 10 local regions. All participants provided written informed consent for participation in this study and for their health status to be monitored by linkage with their electronic health records.

Established CVD risk factors included SBP that was measured as the mean of the last 2 measurements that were recorded after 5 min in the seated position [20]. Plasma lipid levels were measured in a subset of the population (*n* = 18,728) using AU680 chemistry analysers (Beckman-Coulter), which used immunoassays for LDL-C and HDL-C, and immunoturbidimetric assays for total cholesterol and triglycerides [21]. Plasma levels of 2923 proteins were measured using the Olink Explore 3072 panel (OLINK Biosciences, Uppsala, Sweden) for a subset of 1971 CAD cases and 2001 sub-cohort controls [22, 23].

### Genome wide genotyping

DNA extraction, genotyping, array design, and population structure analyses for the CKB have been previously described [24]. Two custom-designed arrays were used to genotype about 800,000 variants in a total of 105,408 CKB DNA samples (Fig. 1). The design of the GWAS subset of the CKB study was constrained by available financial resources for genotyping and limited to 105,408 samples (33,408 selected for nested case-control studies of CVD and COPD and 72,000 participants from a random sample of the CKB study population). On the basis of disease follow-up to 1 January 2014, this initial genotyping included all incident cases of intracerebral haemorrhage (ICH; *n* = 5,020), subarachnoid haemorrhage (SAH; *n* = 455), and fatal IHD (*n* = 753); randomly selected incident cases of ischaemic stroke (IS; *n* = 5,662), MI (*n* = 1,008), and COPD (*n* = 5,376); participants with no CVD events (*n* = 10,038) at the time of this study. The updated array design was then used to genotype a further 72,000 population samples (thereafter referred to as “population-subset”), including all available additional cases of ICH (*n* = 602), SAH (*n* = 46), MI (*n* = 1,028), and fatal IHD (*n* = 163) that had not previously been genotyped. After exclusion of samples on the basis of call rate, excess hetero- or homozygosity, sex mismatch, XY aneuploidy, and non-East-Asian ancestry, genome-wide genotype data were available on 100,706 participants. Using ~532,000 variants that passed quality control for both array versions, imputation into the TOPMed and WBBC reference panels [22] yielded ~50 million variants with an info score >0.3. Genetic analyses used genotypes extracted as dosages of the minor alleles according to the imputed probability of each genotype. Details of national and regional principal components derived for CKB have been previously published [25].

### CAD, stroke, and other vascular diseases

Disease outcomes were obtained by electronic linkage via a unique national identity number to death and disease registries (CAD, stroke, cancer, and diabetes) and to the national health insurance system, which records all inpatient hospital events. Data on all incident disease outcomes were integrated and standardised using the International Classification of Diseases 10th Edition (ICD-10) coded incident disease outcomes. Incident cases of CAD were defined as follows (Table S1): (i) acute MI (ICD-10 codes: I21–I23); (ii) MCEs, defined as either acute MI (I21–I23) or death from IHD (underlying cause of death, I20–I25); and (iii) IHD mortality (defined as any mortality event with ICD-10 I20–I25). In addition, incident cases of 8 other CVD outcomes were defined as follows: ischaemic stroke (IS: I63), intra-cerebral haemorrhage (ICH: I61), all stroke, (I60–61 and I63–64), vascular death (fatal I00–I99 and R96), chronic IHD (I25), hypertensive heart disease (I11), pulmonary heart disease (I26–I28), and heart failure (I50). The diagnostic accuracy study of IHD subtypes included retrieval of medical records from 19,135 reported first-incident IHD cases in CKB for clinician adjudication and reported that 10.2% (*n* = 1,948) of IHD cases had MI, with a diagnostic accuracy of 98% for reported MI and 93% for all IHD, respectively [17].

Logistic regression was used to assess associations of CAD SNPs with CAD or other IHD types; cases with MCE (*n* = 4,748) and chronic IHD (*n* = 9,000) were compared with controls within the population subset (*n* = 62,107) (Fig. 1A). Likewise, analyses of GS-CAD with stroke, CVD death and heart failure, stroke cases (*n* = 10,208), vascular deaths (*n* = 6,375), and HF cases (*n* = 1229) were compared with 62,107 controls in the population subset (Fig. 1B). Linear regression was used to assess associations of GS-CAD with BP (*n* = 70,364), BMI (*n* = 70,363), and LDL-C (*n* = 5,764), respectively. Phenome-wide association analysis (PheWAS) of GS-CAD for common incident major diseases in 62,107 control participants from the population subset (Fig. 1C), and logistic regression was used to assess associations with all common diseases, involving 200 or more first incident cases with GWAS data (Fig. 1D). Linear regression was used to assess associations of GS-CAD with 2,923 proteins in 1962 CAD cases and 1,942 controls (Fig. 1E).

### Statistical analyses

#### Replication of associations of European CAD variants with CVD in Chinese

Logistic regression models were used to assess associations with each CVD subtype in each region, after adjusting for age at baseline, age squared, ever smoking (defined as current or former regular smoking), sex, and the appropriate number of regional principal components per region. The region-specific results were then combined using an inverse-variance weighted (IVW) meta-analysis. Group-specific variances and 95% CI were used for categorical variables to enable comparisons between any 2 categories (including the reference category) [26, 27]. After aligning effect alleles, Pearson correlation coefficients were used to compare the coefficients for CAD outcomes and minor allele frequencies across ancestries. For assessment of replication, analyses were limited to SNPs with an allele frequency >0.01 and an info score >0.8 in the CKB. SNPs with a minor allele frequency <0.01 in East Asians and indels were excluded.

#### Construction of a GS for CAD

Figure 1 shows a flow diagram of the study design and number of participants in genetic analyses of CAD SNPs with MCEs and chronic IHD, other CVD, CVD risk factors, and PheWAS with all common major disease outcomes in CKB. Figure S1 shows the number of individual SNPs and GS combining these SNPs in CKB. GSs for CAD were estimated using the dosages of the 224 European-ancestry genome-wide significant SNPs in CARDIOGRAMplusC4D (CC4D) that were available in CKB. The weighted GS-CAD score was computed using weights based on the log ORs for CAD in European ancestry participants in CC4D using the “riskScore” function from the PredictABEL R package [28], and standardized using a *Z*-score (GS–GS mean)/(GS standard deviation). Participants were assigned to fifths of the GS-CAD score to assess the shape of the association with CVD outcomes.

#### Associations of European GS-CAD with CAD, stroke, and other CVD outcomes in Chinese

Analyses of associations with CVD risk factors were limited to the population sub-cohort for BP (*n* = 70,364) and BMI (*n* = 70,363) (Fig. 1). The associations with LDL-C were performed in a subset of 5,764 controls with no history of CAD or stroke. The associations of fifths of a GS-CAD score with CAD and other CVD outcomes were assessed separately by region using logistic regression after adjusting for age at baseline, age squared, ever smoking, sex, and regional principal components. Likewise, the associations of fifths of GS-CAD with levels of CAD risk were assessed using region-specific linear regression models, after adjusting for age at baseline, age squared, ever smoking, sex, and regional principal components.

#### Phenome-wide association analyses

A PheWAS was conducted using 117 disease phecodes, where each had 200 or more cases in the population subset of genotyped individuals in CKB. Phecodes were curated groups of ICD codes for incident cases of common major diseases with available GWAS data [29, 30]. Phecodes mapping and definitions were obtained from the Center for Precision Medicine, Vanderbilt University Medical Center, Nashville, USA [29, 30]. Logistic regression analyses were conducted for each phecode, and adjusted for age at baseline, age squared, sex, region, smoking, and the first 11 national principal components. A Bonferonni significance level was estimated after taking account of the number of phenotypes.

#### Proteome-wide analyses

A proteome-wide analysis was conducted in 3,977 participants with available data on 2,923 proteins in CKB that were assayed using the Olink Explore panel to assess associations of GS-CAD with plasma proteomics (Fig. 1). Linear regression analyses were used for each protein, adjusted for age at baseline, age squared, sex, region, smoking, plate ID, and the first national 11 principal components. A 5% false discovery rate correction was used, and a Bonferonni threshold was also estimated based on the number of protein associations tested.

*Cis*-protein quantitative trait loci (*cis-*pQTLs), defined as the *cis*-variants associated with plasma levels of 2,923 proteins at GWAS significance level, were used as instrumental variables for MR analyses [31]. Region-specific logistic regression models were used to investigate the associations of *cis-*pQTLs with MCE, after adjustment for age, age squared, sex, ever smoking, and the appropriate number of regional PCs. The results were subsequently combined using IVW meta-analysis using the Wald ratio [31]. Directionality of the associations was assessed using Steiger filtering [32]. In addition, one sample MR was conducted as a sensitivity analysis, limiting the sample to the proteomics subset, using the OneSampleMR package [33].

## Results

The baseline characteristics of CKB participants with GWAS data are shown separately for cases with MCEs or chronic IHD and controls in Table 1. Overall, compared with controls, cases with MCE were older, and more likely to be male, but similar proportions of cases and controls lived in urban and rural areas. The proportions of ever-smokers in Chinese were much greater in male cases and male controls than in female cases or female controls. MCE cases had a higher prevalence of diabetes or hypertension than controls. Likewise, MCE cases had higher mean levels of SBP and DBP than controls, respectively. Conversely, mean levels of BMI did not differ between MCE cases and controls.

**Table 1:** Baseline characteristics of participants with acute and chronic CAD and controls

Mean (SD) or %	Major coronary events (*n* = 4,748)	Chronic IHD (*n* = 9,000)	Controls (n = 62,107)
Demographic factors			
Age, mean (SD), year	62.2 (9.6)	58.9 (9.7)	51.1 (10.4)
Female, %	43.1	61.8	59.3
Urban, %	42.0	56.5	42.8
Lifestyle factors			
Male ever-smokers, %	78.0	75.3	74.4
Female ever-smokers, %	9.5	7.4	3.0
Male ever-drinkers, %	41.8	46.8	49.7
Female ever-drinkers, %	4.1	6.0	4.2
Medical history			
Diabetes^a^, %	16.6	11.1	5.0
Hypertension^a^, %	26.4	24.0	8.9
Clinical measurements			
SBP, mean (SD), mmHg	146 (25)	138(24)	131 (21)
DBP, mean (SD), mmHg	81 (13)	80 (12)	78 (11)
BMI, mean (SD), Kg/m^2^	23.8 (3.9)	24.6 (3.9)	23.6 (3.4)

a Self-reported doctor-diagnosed diabetes or diabetes identified by blood glucose at baseline; MCE: major coronary events, chronic IHD: chronic ischaemic heart disease; SBP: systolic blood pressure; DBP: diastolic blood pressure; BMI: body mass index.

### Cross-ancestry comparisons

**Figure 2:**
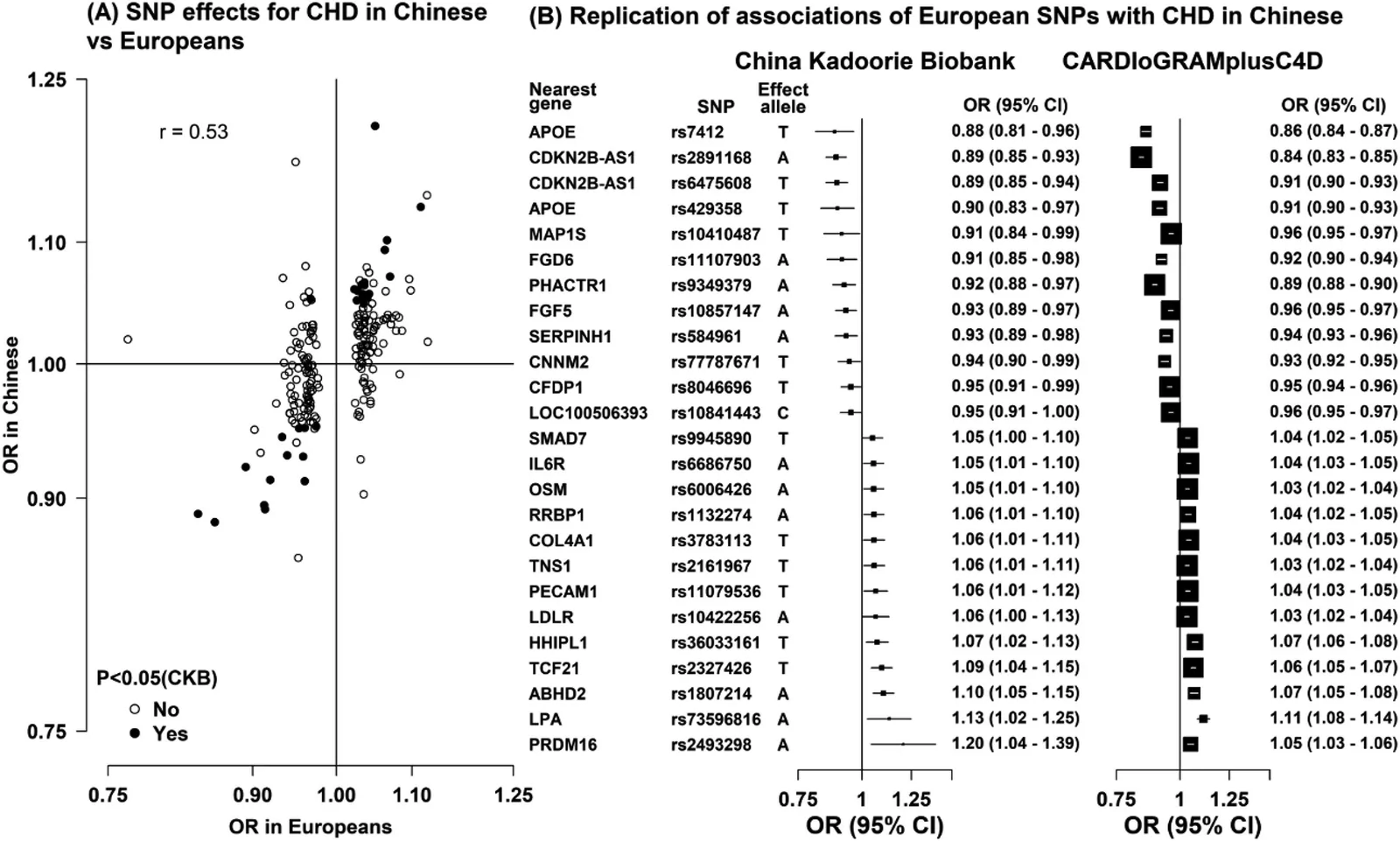
Odds ratio (OR) and 95% CI of (A) MCEs in Chinese for 224 individual CAD SNPs discovered in Europe; and (B) OR and 95% CI of MCEs in Chinese for a subset of 25 SNPs that were replicated in Chinese.

Details of the SNPs selected for analysis in the CKB are provided in Fig. S1. A total of 224 SNPs that were significantly associated with MCE in CC4D were available in CKB. Overall, the effect estimates for the 224 CC4D SNPs were positively correlated with CAD in both ancestries (correlation coefficient = 0.53, Chinese in CKB and Europeans in CC4D: Fig. 2A). A total of 25 European SNPs were significantly associated with MCE (*P* < 0.05) and the associations for these SNPs with acute CAD in Chinese and Europeans were directionally consistent in both populations (Fig. 2B, Table S2). Among these SNPs for CAD in CKB, 11 were previously associated with BP, 7 with lipids, 3 with diabetes, and 3 with BMI [9].

### Associations of GS-CAD with CAD, stroke, and other CVD subtypes

**Figure 3:**
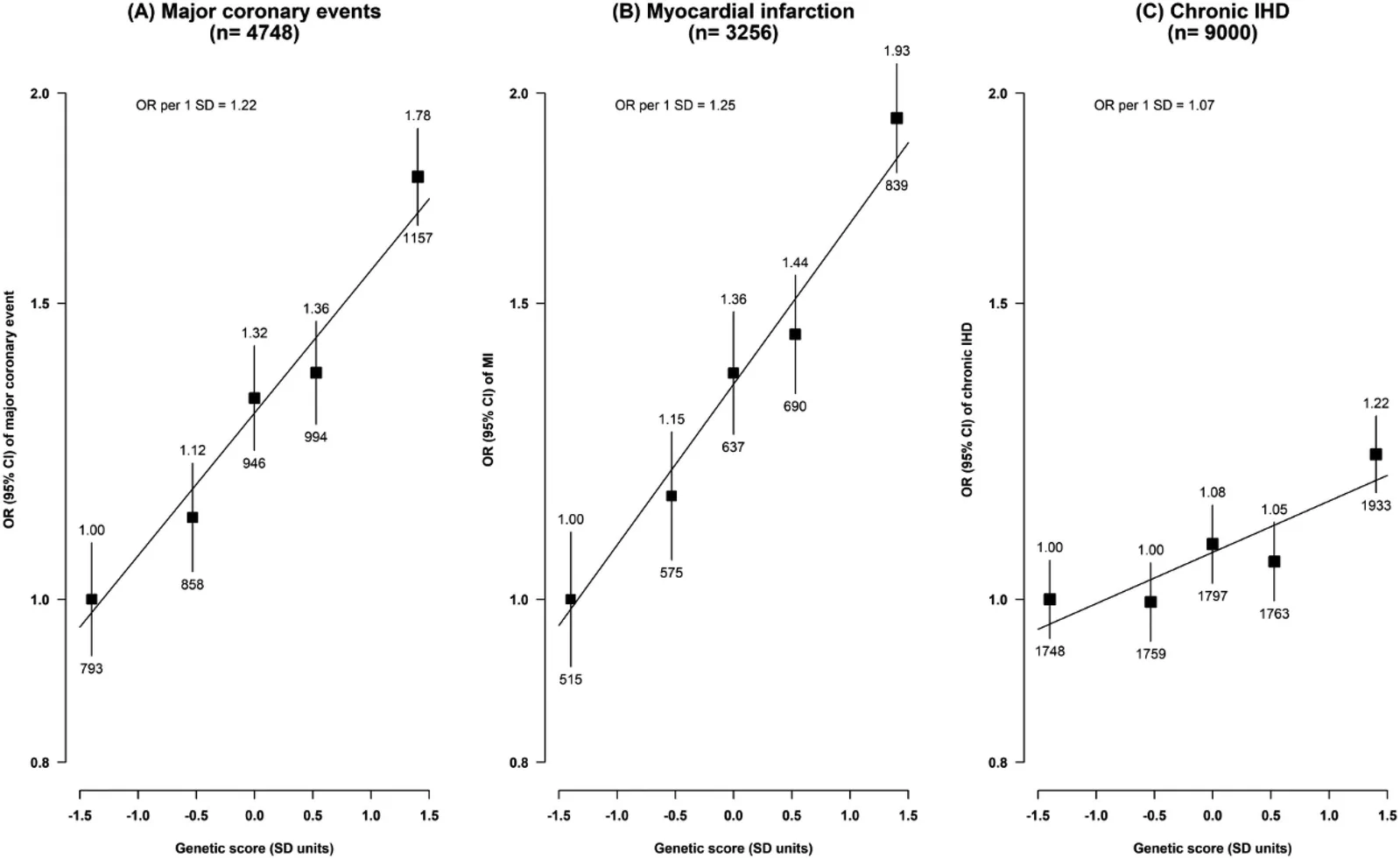
OR and (95% CI) of (A) MCEs, (B) MI, or (C) chronic IHD in Chinese for fifths of a genetic score (GS-CAD) for 224 CAD SNPs discovered in Europeans.

**Figure 4:**
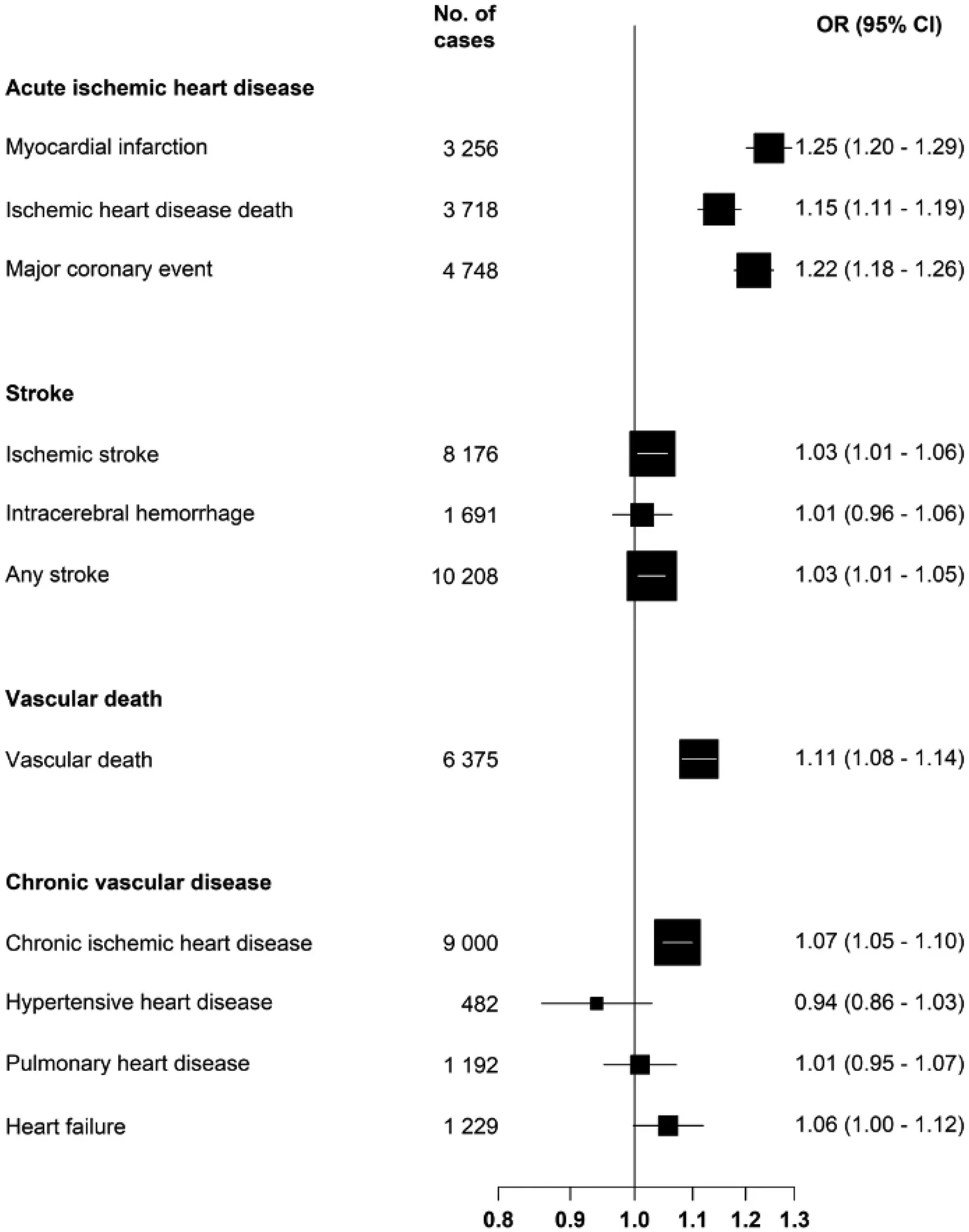
OR and (95% CI) of CAD, stroke, and other vascular diseases in Chinese for a 1 SD higher GS for 224 CAD SNPs discovered in Europeans.

Analyses of associations of fifths of GS-CAD GS with MCE, MI, or chronic IHD indicated linear positive associations with different IHD types, but the strength of the associations (OR, 95% CI) with MCE were 3-fold greater than for chronic IHD (Fig. 3: OR and 95% CI for MCE:1.22 [95% CI:1.18–1.26]; MI:1.25 [1.20–1.29]; and chronic IHD:1.07 [1.05–1.10] per 1 SD higher GS-CAD, respectively). The effects of 1 SD higher GS-CAD for 11 different CVD outcomes were examined in CKB (Fig. 4). A 1 SD higher level of GS-CAD score was associated with a higher risk of MCE, MI, fatal IHD, vascular death, and chronic IHD. However, a 1 SD higher level of GS-CAD was only weakly associated with a higher risk of any stroke (chiefly IS, but not with ICH) or with heart failure, but not at all with hypertensive heart disease or pulmonary heart disease. The top 3 variants most strongly associated with higher risks of MCEs in Chinese included *APOE* and 2 variants for *CDKN2B-AS1* and the top 3 variants inversely associated with MCE included *PRDM16, LPA*, and *ABHD2*, respectively, consistent with the findings in Europeans. Overall, the OR (95% CI) for MCE for a GS for all 224 SNPs was similar to the OR for a subset of 25 variants that were successfully replicated (*P* < 0.05) with MCE in CKB (1.22; [1.18–1.26] vs. 1.21 [1.17–1.25]: Table S3).

### Associations of GS-CAD with CVD risk factors

**Figure 5:**
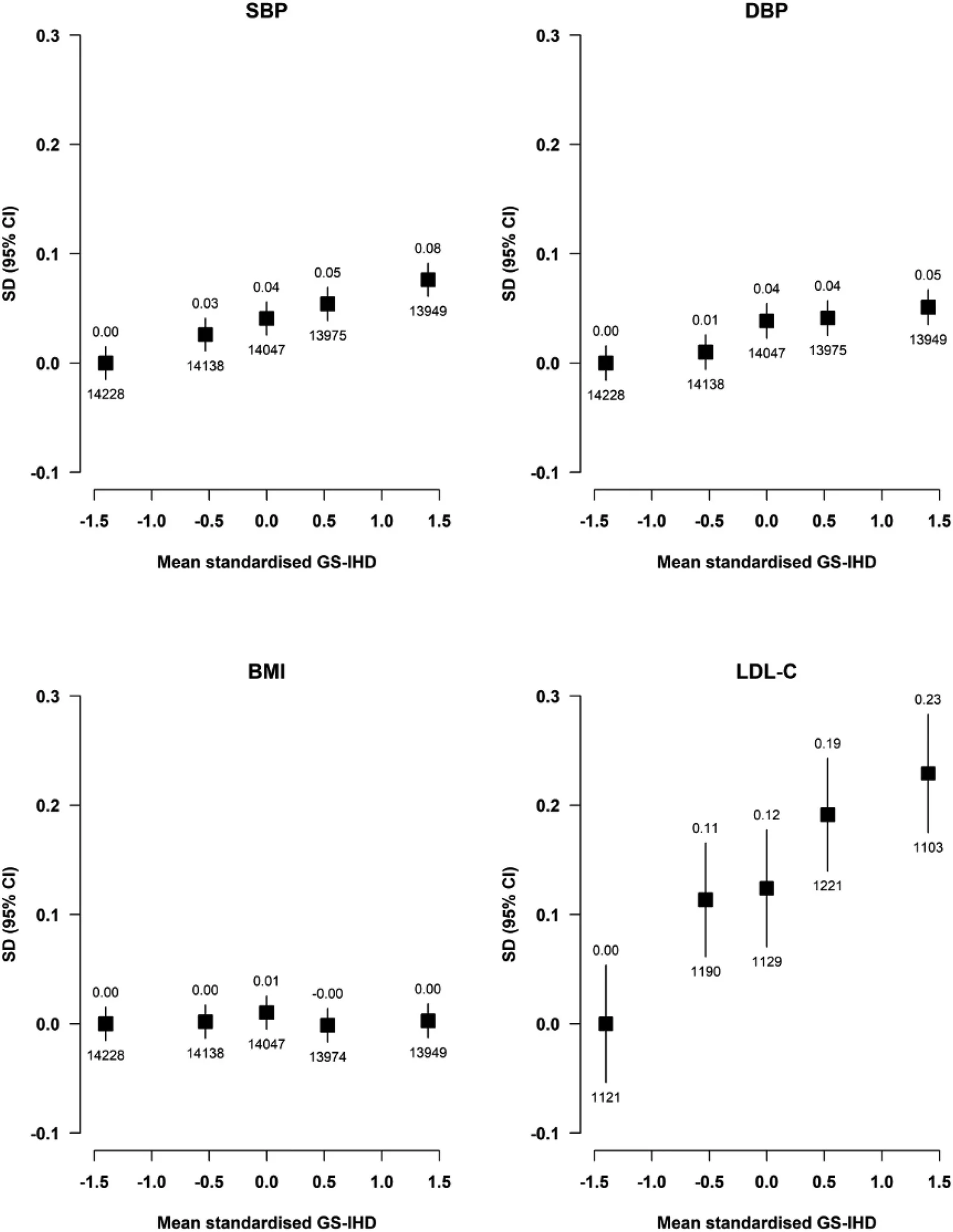
Associations of fifths of a GS for 224 CAD SNPs discovered in Europeans with BP, BMI, and LDL-cholesterol in Chinese. Values shown are line plots for 1 SD higher levels of SBP, diastolic blood pressure (DBP), BMI, and LDL-cholesterol (LDL-C) versus mean standardised genetic score (GS-CAD) per quintile. The number of participants included in each quintile is indicated below each data point.

Figure 5 shows associations of fifths of a genetic risk score for acute CAD (GS-CAD) with mean levels of CVD risk factors including (A) SBP; (B) DBP, (C) BMI, and (D) LDL-C, respectively. The findings indicated strong linear positive associations of increasing fifths of GS-CAD with higher levels LDL-C, but only moderately strong associations with higher levels of SBP and weaker associations with DBP, but no associations with BMI (Fig. 5).

### PheWAS of GS-CAD score with other CVD and non-CVD disease outcomes

A total of 15 major diseases, involving 117 phenotypes, were tested in phenome-wide analyses for associations with a 1 SD higher GS-CAD GS (Fig. S2). Three major diseases were significantly associated with the GS-CAD after correction for multiple testing: MI (OR: 1.26; 95% CI: 1.20–1.33), coronary atherosclerosis (OR: 1.10; 95% CI: 1.07–1.13) and occlusion of cerebral arteries (OR: 1.04; 95% CI: 1.02–1.08), respectively, highlighting the specificity for these associations.

### Associations of GS-CAD score with plasma proteomics

Analyses of associations of GS-CAD with plasma levels of 2,923 proteins indicated that a 1 SD higher score was associated with higher levels of 2 proteins after correction for multiple testing: Lipoprotein-associated phospholipase A2 (PLA2G7: coefficient 0.05; 95% CI: 0.03–0.07) and Sushi-containing domain protein (SUSD2: coefficient: −0.02; 95% CI: -0.01–0.04) (Fig. S3). However, there was an association of GS-CAD with SUSD2 in a sensitivity analysis after excluding variants within 1 mB of the causal gene region for each protein. Furthermore, 2 *cis*-PQTLs were identified, one for each of the 2 proteins (PLA2G7 and SUSD2: Table S4), but both one-sample and 2-sample MR analyses provided no causal support for CAD for either of these 2 proteins (Fig. S4).

## Discussion

This study examined the associations of 224 SNPs for CAD that were discovered in European studies with CAD in Chinese adults and confirmed a moderate concordance of genetic determinants of CAD between these 2 diverse ancestry populations. Despite a relatively limited number of SNPs (*n* = 25) being nominally significantly associated with CAD (at *P* < 0.05) in CKB, a weighted GS-CAD score using weights derived from the European population was strongly associated with CAD in Chinese, but only weakly associated with chronic IHD or CVD mortality in Chinese adults. Individuals in the top vs. bottom fifths of a genetic score (GS-CAD) for CAD were associated with a 2-fold higher risk of MI, but with only a 20% higher risk of chronic IHD. The GS-CAD score was only weakly associated with a higher risk of IS, and was unrelated with HS.

The GS-CAD score was also strongly associated with mean levels of LDL-C and SBP, and to lesser extent with DBP, but not with BMI. A PheWAS analysis for 117 phenotypes in Chinese adults indicated that the GS-CAD score was significantly associated with higher risk of MI, coronary atherosclerosis, and occlusion of the cerebral arteries, respectively. Analysis of associations of GS-CAD with 2923 proteins demonstrated associations with PLA2G7 and SUSD2, but MR analyses did not provide any causal support for associations of either of these 2 proteins with CAD.

The number of CVD deaths and proportions of total deaths due to CVD have increased exponentially in China compared with Western countries in recent decades [34]. Several studies have also reported a greater disease severity for CAD in East Asians than in Europeans [35]. Heritability of CAD has been estimated to be ~40–60% in twin studies [15] (with the independent variants for IHD identified in the present study explaining ~36% of heritability); it is likely that most of the differences in incidence of acute CAD or CAD severity between Europeans and East Asians may reflect differences in diet and lifestyle or variable access to medical treatment [9]. Comparing the associations of genetic variants for CAD in different genetic ancestries can help to understand the genetic architecture for CAD in diverse populations. Moreover, as most GWAS studies have been conducted in European populations, the generalizability of the resulting GWAS or GS-CAD GSs for CAD in other ancestries will require further validation in future cycles of international GWAS consortia of CAD that will include a greater number of CAD cases from East Asian populations [36]. A previous study of associations of European ancestry GS-CAD GS with CAD in non-European participants in UK Biobank reported a good correlation across most included ancestries, except for Africans, but the analyses were based on small numbers of CAD cases from non-European ancestry populations [37]. The percentage of replicated SNPs with CAD and correlation between effect sizes for CAD between Europeans and Chinese were consistent with those previously reported between European and Japanese ancestry populations [9].

Moreover, a European ancestry GS-CAD score was associated with a higher risk of coronary artery atherosclerosis and cerebral artery occlusion in phenome-wide analyses. The results are consistent with a shared aetiology of atherosclerotic diseases between Chinese and European ancestries. With the exception of LDL-C and SBP, the GS-CAD was not strongly associated with other CAD risk factors [38, 39], but the study included too few CAD cases with GWAS data and plasma lipids at baseline to conduct formal mediation analyses of CAD by levels of LDL-C and SBP.

In a separate comparative report of GWAS associations of Lp(a)-related variants with Lp(a) levels in Chinese vs. Europeans in the CKB study, we demonstrated only a modest overlap of Lp(a)-related variants with Lp(a) levels in both populations with only a single SNP rs73596816 being strongly associated with Lp(a) levels in both Chinese and Europeans [40]. In contrast, the 2 lead SNPs most strongly associated with Lp(a) levels and MI in Europeans [41] were unrelated to Lp(a) or MI in Chinese with rs10455872 being very rare and rs3798120 SNP having only a minimal effect on Lp(a) levels in Chinese. However, our previous study reported that the rs73596816 SNP was strongly related to Lp(a) levels and risk of MI in both Chinese and East Asian populations. The present report confirmed the comparable strength of associations of rs73596816 with risk of MI in both ancestry populations (OR [95% CI]: 1.13 [1.02–1.25] vs. 1.11 [1.08–1.14] in Chinese vs. Europeans, respectively) [40]. Despite differences in the genetic architecture of the *LPA* locus between Chinese and Europeans, both the present and previous reports demonstrated that equivalent genetically predicted differences in Lp(a) levels had comparable effects on MI in both ancestry populations [40, 41].

The present analysis demonstrated only a weak positive association of the GS-CAD score with ischaemic stroke or occlusion of cerebral arteries in a PheWAS analysis in addition to MI and coronary atherosclerosis. Hence, the results of this study add to a growing body of evidence highlighting the shared biological pathways between acute CAD and large artery ischaemic stroke and provide further support for lowering LDL-C and SBP for prevention of occlusive atherosclerotic vascular diseases in both populations.

In contrast, we found little evidence of shared genetic pathways between CAD with hypertensive or pulmonary heart diseases. Previous studies suggested some shared heritability between CAD and ischaemic stroke [42], and a significant overlap of genetic variants between ischaemic stroke and CAD [43]. Importantly, 6 known CAD risk loci, including 9p21 (the locus with the smallest *P*-value in our replication analysis), were previously associated with ischaemic stroke [44]. Despite the 9p21 locus being one of the most studied variants for IHD, the mechanism of action of this locus on risk of CAD has yet to be elucidated. Importantly, the 9p21 locus has been previously associated with atherosclerosis, but not with thrombosis or plaque rupture [45]. The findings suggest that 9p21 may play a role in both CAD and acute IS, mediated partly through large artery atherosclerosis in both Chinese and European populations.

The chief strengths of the present study include the large number of cases of CAD, stroke, and other CVD outcomes and CVD risk factors from a single prospective study in Chinese adults recruited from 10 diverse geographical regions in China [35, 46]. Prospective cohort studies offer several major advantages beyond case-control studies, especially when the goal is to understand how exposures relate to outcomes over time. Cohort studies also enable analyses of the temporal sequence and measurement of incident disease and study of multiple outcomes from single exposures. However, the study also had several limitations. First, the number of cases of MI, MCE, or IHD was too limited to have sufficient statistical power to replicate associations with lower frequency variants or variants with smaller effect sizes. We estimate that the present study had about 88% power to detect MCE associations with variants with a MAF of 0.2 and an OR of 1pql.1 at our Bonferroni significance level (0.05/224), with lower power to detect associations with variants with lower MAF (e.g., 16% for MAF of 0.05 and OR of 1.1) [47]. Second, we excluded phenotypes with less than 200 participants in our PheWAS analysis, and, hence, could have missed some phenotypes associated with GS-CAD score [30]. An alternative explanation for the null associations of CAD-GS with plasma proteomics suggests that CAD SNPs may be involved in initiation of atherosclerosis of the coronary arteries, but plasma proteomics may be more closely related to determinants of progression of atherosclerosis.

## Conclusions

Overall, this study demonstrated a moderate concordance of genetic determinants of acute CAD between European and Chinese adults, and a GS-CAD GS combining all such variants was moderately strongly associated with acute CAD in Chinese adults. However, the strength of association of this GS-CAD GS with acute CAD was 3-fold stronger than that for chronic IHD or heart failure and 7-fold stronger than that for stroke. The GS-CAD score was also strongly associated with levels of LDL-C or SBP, but only moderately associated with BMI. The results highlight a shared genetic architecture of CAD between Europeans and Chinese that is partly mediated through levels of LDL-C and SBP and provides support for lowering mean levels of these risk factors for prevention of CAD in Chinese population. When the currently planned cohort-wide whole genome sequence data are available on all 0.5 M participants in CKB, it should be possible to conduct further analyses of the concordance of CAD genes for rarer variants between Europeans and Chinese in addition to CAD associations with additional individual genes, gene–gene interactions, or gene–environmental interactions between Chinese and European populations.

## Availability of source code and requirements

- Project name: Edris-2025-genetic-determinants-of-acute-coronary-artery-disease
- Project repository: https://github.com/ckbiobank/Edris-2025-genetic-determinants-of-acute-coronary-artery-disease
- License: MIT license
- Operating system(s): Platform independent
- Programming language: R Project for Statistical Computing
- RRID:SCR_001905

## Abbreviations

BBJ, Biobank Japan; BMI, body mass index; CC4D, CARDIoGRAMplusC4D; CKB, China Kadoorie Biobank; CDC, Center of Disease Control; CVD, cardiovascular diseases; DBP, diastolic blood pressure; ECG, electrocardiogram; FDR, false discovery rate; GS, genetic score; GS-CAD, genetic risk score for CAD; GWAS, genome-wide association studies; HDL-C, high-density lipoprotein cholesterol; IHD, ischaemic heart disease; LDL-C, low-density lipoprotein cholesterol; Mb, megabase; MCE, major coronary events; MI, myocardial infarction; MR, Mendelian randomization; PheWAS, phenome-wide association studies; PLA2G7, lipoprotein-associated phospholipase A2; pQTLs, protein quantitative trait loci; QC, quality control; SBP, systolic blood pressure; SNPs, single nucleotide polymorphisms; SUSD2, Sushi-containing domain protein; TOPMed, Trans-Omics for Precision Medicine; WBBC, West Lake Biobank for Chinese; WHR, waist-to-hip ratio.

## Supplementary Material

giag084_Supplemental_File

giag084_Authors_Response_To_Reviewer_Comments_Original_Submission

giag084_GIGA-D-25-00454_Original_Submission

giag084_GIGA-D-25-00454_Revision_1

giag084_Reviewer_1_Report_Original_Submission Reviewer 1 -- 1/4/2026

giag084_Reviewer_1_Report_Revision_1 Reviewer 1 -- 5/8/2026

giag084_Reviewer_2_Report_Original_Submission Reviewer 2 -- 3/16/2026

giag084_Reviewer_2_Report_Revision_1 Reviewer 2 -- 5/8/2026

## Data Availability

Data for this study is described on CKB Research Tracker (DAR-2023-00342: Ahmed Edris Mohamed). Data on the analyses of associations of the 224 CAD SNPs for acute CAD in Europeans [9] and results in Chinese are available under the University of Oxford’s Open Source International (MIT) license. The code used for data analysis, the data underlying the figures presented in this study, and the summary results on associations of the 224 CAD SNPs with acute CAD and other vascular outcomes in CKB are included in the CKB Project GitHub [48]. The China Kadoorie Biobank (CKB) is a blood-based prospective study of 512,000 Chinese adults. The data for the present report are accessible to investigators via the CKB study website [49]. Details about the study are provided on the CKB data access pages [50]. Data from baseline, first and second resurveys, and disease follow-up are available under the CKB Open Access Data Policy to bona fide researchers. Sharing of genotyping data is constrained by the Administrative Regulations on Human Genetic Resources of the People’s Republic of China. Access to these and certain other data is available through collaboration with CKB researchers. Genotyping data were exported from China to the Oxford CKB International Coordinating Centre under Data Export Approvals 2014-13 and 2015-39 from the Office of Chinese Human Genetic Resource Administration.

## Additional files

**Supplementary Figure S1:** Distribution of SNPs from European and Japanese populations in the study.

**Supplementary Figure S2:** Phenome-wide association of a genetic risk score for CHD with all common disease outcomes in CKB.

**Supplementary Figure S3:** Proteome-wide analysis of GS-CHD scores with 3977 proteins.

**Supplementary Figure S4:** MR results for PLA2G7 and SUSD2 effects on MCE in CKB.

**Supplementary Table S1:** Study end-points and associated ICD-10 codes.

**Supplementary Table S2:** OR (95% CI) of major coronary events in CKB for variants identified in a meta-analysis of CARDIoGRAMplusC4D and Biobank Japan.

**Supplementary Table S3:** Phenotypes associated with replicated acute IHD variants in PheWAS of CKB.

**Supplementary Table S4:** Comparison of OR (95% CI) of major coronary events for 1 SD higher genetic score for 224 European SNPs for CAD with subset of 25 SNPs that were replicated at *P* < 0.05 in CKB.

**Supplementary Table S5:** Distribution of pQTLs identified for SUSD2 and PLA2G7 proteins.
