# Supplementary material for: Concordance of genetic determinants of acute coronary artery disease between European and Chinese populations
Source: Gigascience. 2026 Aug 11;15:giag084. doi: 10.1093/gigascience/giag084 (PMC13543311; doi:10.1093/gigascience/giag084)
Supplement: Supplementary file 1 [file giag084_authors_response_to_reviewer_comments_original_submission.pdf]

Author's Response To Reviewer Comments

RESPONSE TO REVIEWER COMMENTS ON GIGA-D-25-00454
"Concordance of genetic determinants of acute coronary artery disease between European and Chinese populations".

The verbatim comments of the two reviewers are outlined using a standard font and our responses to their comments and suggestions are made using a bold font. In addition, we itemized the page numbers where we made the revisions to the manuscript.

Reviewer #1:

1.1 Such studies are often in large size and may draw attention from the editors and readers. However, the authors may need to explain the clinical relevance of such studies either in the Introduction or in the Discussion, especially after so many high-cost GWAS studies of various technological platforms had been published in the past about three decades. Did these studies ever help in any sense or manner for health ?
RESPONSE: Genetic studies of complex-trait diseases have greatly advanced our knowledge of the genetic architecture of ischaemic heart disease, elucidation of causal relevance of risk factors and discovery of novel targets for treatment. Advances include three main fields: (A) Understanding the biological origins of cardiovascular disease (CVD) (B) discovery of known and novel targets for drug treatment of CVD; (C) improving risk prediction through polygenic risk scores. We have added a paragraph to the Introduction on pages 5/6 to state the following: "Genome-wide association (GWAS) studies of CVD conducted in diverse ancestry populations have greatly expanded our knowledge of risk factors for CVD, by clarifying the genetic architecture of CVD across populations, elucidation of instrumental variables for Mendelian randomization (MR) analyses to assess the causal relevance of risk factors, discovery of novel targets for drug treatment of CVD and development of polygenic scores to improve CVD risk prediction.(9-16) While meta-analyses of international consortia of GWAS studies have helped to elucidate cross-ancestry differences in CVD traits and subtypes, large biobanks like UK Biobank, Japan Biobank and China Kadoorie Biobank can also provide additional information about differences between phenotypes, gene-gene and gene-environmental interactions that are not possible to discover in multi-ancestry meta-analyses of GWAS consortia of CVD sub-types." (17-19)

1.2. Another major issue is the study design. In this study, the cases are from a large population-based study. There may be many strengths of this study design. There are apparently also limitations. A possible major limitation of such a study design is the diagnostic accuracy of the diseases under study. Thus, the authors may need to compare this kind of studies with studies based on clinical cases collected from hospitalized patients.
RESPONSE: Prospective cohort studies offer several major advantages beyond case-control studies, especially when the goal is to understand how exposures relate to outcomes over time. Cohort studies also provide the ability to establish temporal sequence, measure incidence, and study multiple outcomes from a single exposure. We added a paragraph in the Introduction on page 5/6: "Genome-wide association (GWAS) studies of CVD conducted in diverse ancestry populations have greatly expanded our knowledge of risk factors for CVD, by clarifying the genetic architecture of CVD across populations, elucidation of instrumental variables for Mendelian randomization (MR) analyses to assess the causal relevance of such risk factors, discovery of novel targets for drug treatment of CVD and development of polygenic scores to improve risk prediction of CVD.(9-16) While meta-analyses of international consortia of GWAS studies have reported cross-ancestry differences in genetic architecture of CVD sub-types, large biobanks such as UK-Biobank, Japan Biobank or China Kadoorie Biobank can provide additional information about differences between CVD sub-types, gene-gene or gene-environmental interactions that are not possible to discover in multi-ancestry meta-analyses of GWAS consortia of summary statistics on CVD sub-types. (17-19)"
The diagnostic accuracy of first incident MI cases in Chinese adults was very high. We revised the sentence on the diagnostic accuracy of reported first incident cases of MI and all IHD cases in CKB on page 10/11: "The diagnostic accuracy study of IHD subtypes included retrieval of medical records from 19,135 reported first-incident IHD cases in CKB for clinician adjudication and reported that 10.2% (n=1948) of IHD cases had MI, with a diagnostic accuracy of 98% for reported MI and 93% for all IHD, respectively."(17)

1.3. GWAS data are only available in about 20% of the total Kadoorie study participants. The authors may need to clarify why. If the selection was for purpose, the authors should also clarify.
RESPONSE: We have revised the description of the study design and selection for GWAS, and the population subset was constrained by available financial resources for genotyping. On page 9, we added the following sentence: "The design of the GWAS subset of the CKB study was constrained by available financial resources for genotyping and limited to 105,408 samples (33,408 selected for nested case-control studies of CVD and COPD and 72,000 participants from a random sample of the CKB study population)." Since the reviewer was concerned about why our analyses were limited to 105,000 with GWAS data, we also added sentence at the end of the report to indicate that ongoing whole genome sequence data will shortly be available on 0.5M participants in CKB that will allow us to compare associations with CAD for lower frequency variants in addition to gene-gene interactions and gene-environmental interactions that may also differ between Chinese and European ancestry populations. On page 24, we added the following statements: "When the currently planned cohort-wide whole genome sequence data are available on all 0.5M participants in CKB, it should be possible to conduct further analyses of the concordance of CAD genes for rarer variants between Europeans and Chinese in addition to CAD associations with additional individual genes, gene-gene interactions or gene-environmental interactions between Chinese and European populations".

1.4. There are several corresponding authors from the UK and several coauthors from China. This reviewer guesses that these Chinese authors might also contribute to this project significantly. Is it worthwhile to have a Chinese expert to be among the corresponding authors for the proper interpretation of the findings.
RESPONSE: As China Kadoorie Biobank is jointly supervised by scientists from Peking University, Beijing, China and University of Oxford, Oxford, England, we have an agreed co-authorship policy for all publications that recognize the contribution of both teams to all research outputs. All projects are reviewed by a Research Management Committee prior to any analyses and recorded on a Research Tracker system. All primary CKB reports that involve CKB data as a primary output include key co-authors from China and Oxford who are members of the steering committee of the study and these include 4 senior PI from China in addition to one PI from one of the 10 regional centers. All authors are recorded on a research management electronic platform that can be audited. We have agreed that reports led by colleagues in China include first and senior authors and corresponding authors from China and vice versa for those reports who are led by colleagues in Oxford. This division is pragmatic and equitable and works best for smooth running of publications led by the CKB Collaborative Study Group.

Reviewer #2:

2.1. Only 25 of 224 European CAD SNPs were nominally replicated in the Chinese dataset, which limits the strength of the conclusion regarding cross-ancestry concordance. The authors should discuss more fully the potential roles of limited statistical power, allele-frequency differences, and differences in linkage disequilibrium structure.
RESPONSE: We have acknowledged these limitations in the Discussion. We estimate that the study had only about 88% power to detect MCE associations with variants with a MAF of 0.2 and an OR of 1.1 at our Bonferroni significance level (0.05/224). We have modified a paragraph on the study limitations to explain the limited statistical power on page 21 as follows: "First, the number of cases of MI, MCE, or IHD were too limited to have sufficient statistical power to replicate associations with lower frequency variants or variants with smaller effect sizes. We estimate that that the present study had about 88% power to detect MCE associations with variants with a MAF of 0.2 and an OR of 1.1 at our Bonferroni significance level (0.05/224), with lower power to detect associations with variants with lower MAF (e.g. 16% for MAF of 0.05 and OR of 1.1).(47)
2.2. The genetic score was constructed using European-derived weights; this is reasonable for testing transferability, but the authors should clarify whether alternative score constructions were evaluated and whether recalibration in the Chinese population changes the results.
RESPONSE: The rationale of the paper was to use conventional methods to estimate the polygenic risk score using European weights, because they were based on much larger sample sizes. In due course, the CKB will obtain genetic data on all 500,000 in the study and then have much greater power to generate improved polygenic scores using Chinese weights. We have acknowledged this limitation in the Discussion

and added a sentence at the end of the Discussion. On Page 24, we state: "When the currently planned cohort-wide whole genome sequence data are available on all 0.5M participants in CKB, it should be possible to conduct further analyses of the concordance of CAD genes for rarer variants between Europeans and Chinese in addition to CAD associations with additional individual genes, gene-gene interactions or gene-environmental interactions between Chinese and European populations.

2.3. The interpretation that the shared genetic architecture is partly mediated through LDL-C and SBP appears stronger than warranted by the presented analyses, which are associative rather than formal mediation analyses. This wording should be softened unless additional analyses are provided. The proteomic analysis is interesting but appears underpowered relative to the large number of proteins tested, so the negative findings should be interpreted more cautiously.
RESPONSE: We have revised the statements on mediation of genetic risk scores for ischemic heart disease by LDL-C and SBP and more importantly by proteomic analyses. We have acknowledged that the moderate sample size of IHD cases precludes formal mediation analyses and added this to the limitations section in the Discussion. On page 19 the modified the statement on LDL-C and SBP states: "With the exception of LDL-C and SBP, the GS-CAD was not strongly associated with other CAD risk factors,(38, 39) but the study included too few CAD cases with GWAS data and plasma lipids at baseline to conduct formal mediation analyses of CAD by levels of LDL-C and SBP."

Editorial Manager®

Cookie Preference Center

We use cookies which are necessary to make our site work. We may also use additional cookies to analyze, improve and personalize our content and your digital experience. For more information, see our Cookie Policy.

You may choose not to allow some types of cookies. However, blocking some types may impact your experience of our site and the services we are able to offer. See the different category headings below to find out more or change your settings.

Allow all

Manage Consent Preferences

Strictly Necessary Cookies

Always active

These cookies are necessary for the website to function and cannot be switched off in our systems. They are usually only set in response to actions made by you which

amount to a request for services, such as setting your privacy preferences, logging in or filling in forms. You can set your browser to block or alert you about these cookies, but some parts of the site will not then work.

Functional Cookies

These cookies enable the website to provide enhanced functionality and personalisation. They may be set by us or by third party providers whose services we have added to our pages. If you do not allow these cookies then some or all of these services may not function properly.

Performance Cookies

These cookies allow us to count visits and traffic sources so we can measure and improve the performance of our site. They help us to know which pages are the most and least popular and see how visitors move around the site.

Targeting Cookies

These cookies may be set through our site by our advertising partners. They may be used by those companies to build a profile of your interests and show you relevant adverts on other sites. If you do not allow these cookies, you will experience less targeted advertising.

Cookie List

Clear

Apply Cancel
Consent Leg.Interest

Confirm my choices

Power
