# Supplementary material for: Concordance of genetic determinants of acute coronary artery disease between European and Chinese populations
Source: Gigascience. 2026 Aug 11;15:giag084. doi: 10.1093/gigascience/giag084 (PMC13543311; doi:10.1093/gigascience/giag084)

GigaScience

Concordance of genetic determinants of acute coronary artery disease between European and Chinese populations

--Manuscript Draft--

**Manuscript Number:**	GIGA-D-25-00454
**Full Title:**	Concordance of genetic determinants of acute coronary artery disease between European and Chinese populations
**Article Type:**	Research
**Funding Information:**	Wellcome Trust (212946/Z/18/Z, 202922/Z/16/Z, 104085/Z/14/Z, 088158/Z/09/Z) — Prof Zhengming Chen<br>Innovative Research Group Project of the National Natural Science Foundation of China (82192901, 82192904, 82192900, 82388102) — Prof Liming Li<br>Noncommunicable Chronic Diseases-National Science and Technology Major Project (2023ZD0510101, 2023ZD0510100) — Prof Liming Li<br>Medical Research Council (MC_UU_00017/1, MC_UU_12026/2, MC_U137686851) — Prof Zhengming Chen<br>British Heart Foundation's Big Beat Challenge award to CureHeart (BBC/F/21/220106) — Prof Hugh Watkins
**Abstract:**	Abstract (n=250)<br>Aims: This study examined the concordance of associations of single nucleotide polymorphisms (SNPs) for acute coronary artery disease (CAD) discovered in Europeans with acute CAD, chronic ischaemic heart disease (IHD) or stroke in Chinese adults.<br>Methods: In a nested case-control study of acute CAD (4748 cases/66,227 controls) in China Kadoorie Biobank (CKB), we compared associations of 224 SNPs for acute CAD discovered in Europeans in CARDIOGRAMplusC4D (CC4D) with acute CAD in Chinese. We compared associations of a genetic score (GS-CAD) for 224 SNPs for acute CAD with chronic IHD (n=9000), heart failure (n=1229), stroke (n=10,208) and CVD risk factors (n=70,364) and plasma proteomics (n=3904) in CKB.<br>Results: Overall, the strength of associations of 224 CAD SNPs in Europeans was moderately correlated with those for acute CAD in Chinese (r=0.53) and significant associations were replicated (p<0.05) for 25 SNPs. The strength of associations with acute CAD for a 1 SD higher genetic score for acute CAD in Chinese were 3-fold greater than for chronic IHD or heart failure (1.22 [1.18-1.26] vs 1.07 [1.05-1.10] vs 1.06 [1.00-1.12], respectively) and 7-fold greater than for stroke (1.03; 1.01-1.05). Higher levels of GS-CAD were strongly associated with LDL-cholesterol (LDL-C), moderately with systolic blood pressure (SBP) and weakly with body mass index (BMI), but not with proteomics.<br>Conclusion: The results demonstrate moderate concordance in genetic determinants for acute CAD between Europeans and Chinese that was strongly related to LDL-C, and to a lesser extent with SBP and BMI, but only weakly associated with chronic IHD or stroke.
**Corresponding Author:**	Robert Clarke<br>Oxford University: University of Oxford<br>Oxford, Oxfordshire UNITED KINGDOM
**Corresponding Author Secondary Information:**	
**Corresponding Author's Institution:**	Oxford University: University of Oxford
**Corresponding Author's Secondary Institution:**	

Powered by Editorial Manager® and ProduXion Manager® from Aries Systems Corporation

First Author:	Robert Clarke, FRCP, MD
First Author Secondary Information:	
Order of Authors:	Robert Clarke, FRCP, MD
	Ahmed Edris, PhD
	Kuang Lin, PhD
	Sofia Massa, PhD
	Iona Y Millwood, DPhil
	Paul Sherliker, MSc
	Liming Li, MPH
	Jun Lv, PhD
	Canqing Yu, PhD
	Dianjianyi Sun, PhD
	Pei Pei, BSc
	Daniel Avery, MSc
	Fang Liu, BSc
	Anuj Goel, MSc
	Hugh Watkins, MD
	Zhengming Chen, DPhil
	Robin G Walters, PhD
	Derrick A Bennett, PhD
Order of Authors Secondary Information:	
Additional Information:	
Question	Response
Are you submitting this manuscript to a special series or article collection?	No
**Experimental design and statistics**<br><br>Full details of the experimental design and statistical methods used should be given in the Methods section, as detailed in our Minimum Standards Reporting Checklist. Information essential to interpreting the data presented should be made available in the figure legends.<br><br>Have you included all the information requested in your manuscript?	Yes
**Resources**<br><br>A description of all resources used,	Yes

including antibodies, cell lines, animals and software tools, with enough information to allow them to be uniquely identified, should be included in the Methods section. Authors are strongly encouraged to cite Research Resource Identifiers (RRIDs) for antibodies, model organisms and tools, where possible.<br><br>Have you included the information requested as detailed in our Minimum Standards Reporting Checklist?	
**Availability of data and materials**<br><br>All datasets and code on which the conclusions of the paper rely must be either included in your submission or deposited in publicly available repositories (where available and ethically appropriate), referencing such data using a unique identifier in the references and in the "Availability of Data and Materials" section of your manuscript.<br><br>Have you have met the above requirement as detailed in our Minimum Standards Reporting Checklist?	No
If not, please give reasons for any omissions below.<br><br>as follow-up to "**Availability of data and materials**<br><br>All datasets and code on which the conclusions of the paper rely must be either included in your submission or deposited in publicly available repositories (where available and ethically appropriate), referencing such data using a unique identifier in the references and in the "Availability of Data and Materials" section of your manuscript.	We can provide summary statistics for all genetic data on publication of the report.

Powered by Editorial Manager® and ProduXion Manager® from Aries Systems Corporation

Have you have met the above requirement as detailed in our Minimum Standards Reporting Checklist?<br><br>"	
GigaScience has policies and guidelines in place for the use of generative AI-writing tools such as ChatGPT. If you have used such writing tools to assist with writing the manuscript this must be declared and cited in the text. Authors should not list AI-writing tools and other AI-assisted technologies as an author or co-author and should acknowledge that they are fully responsible for text generated or refined by AI-writing tools.<p><br><br>A summary of use (particularly in the introduction or among methods) needs to be included at the end of the paper, and the outputs should also be included as a supplementary file hosted in GigaDB or other open repositories. Please read our guidelines for more information. <p><br><br>By submitting to GigaScience, you are aware of the journal's AI-writing tools policy, and if you have declared use of such tools below, you have acknowledged this where appropriate in your manuscript and have made a summary of use and outputs available. <p><br><b>AI-assisted writing tools have been used in the preparation of this manuscript?	No

Manuscript Click here to access/download;Manuscript;CC4D paper-4-11-2025.docx

Concordance of genetic determinants of acute coronary artery disease between European and Chinese populations

Ahmed Edris PhD[1], Kuang Lin PhD[1], Sofia Massa PhD[1], Iain Turnbull MB,[1] Iona Y. Millwood DPhil[1], Paul Sherliker MSc[1], Liming Li MPH[2,3,4], Jun Lv PhD[2,3,4], Canqing Yu PhD[2,3,4], Dianjianyi Sun PhD[2,3,4], Pei Pei BSc[3], Daniel Avery MSc[1], Fang Liu BSc[5], Anuj Goel MSc[6], Hugh Watkins MD[6], Zhengming Chen DPhil[1], Robin G. Walters PhD[1*], Derrick A. Bennett PhD[1*], Robert Clarke MD[1*] on behalf of the China Kadoorie Biobank Study Group[†]

[1] Clinical Trial Service Unit, Nuffield Department of Population Health, University of Oxford, Oxford, UK;

[2] Department of Epidemiology & Biostatistics, School of Public Health, Peking University, Beijing, China;

[3] Peking University Center for Public Health and Epidemic Preparedness and Response, Beijing, China;

[4] Key Laboratory of Epidemiology of Major Diseases, Peking University, Ministry of Education, Beijing, China;

[5] Suzhou CDC, Suzhou, Jiangsu, China;

[6] Division of Cardiovascular Medicine, Radcliffe Department of Medicine, University of Oxford, Oxford, UK.

*Equal contribution; [†] Members of the CKB Study Group are shown in the appendix.

Address for correspondence:

Professor Robert Clarke	or	Professor Derrick Bennett
Nuffield Department of		Nuffield Department of
Population Health		Population Health
Old Road Campus		Old Road Campus
Roosevelt Drive		Roosevelt Drive
Oxford		Oxford
OX3 7LF		OX3 7LF
United Kingdom		United Kingdom
Email: robert.clarke@ndph.ox.ac.uk		derrick.bennett@ndph.ox.ac.uk

4 November 2025

Short title: Genetic determinants of acute coronary artery disease

Word count: Abstract 250; Text: 4547

Key words: Genetic determinants, acute coronary artery disease, stroke, other vascular diseases, Chinese and European populations.

Abstract (n=250)

Aims: This study examined the concordance of associations of single nucleotide polymorphisms (SNPs) for acute coronary artery disease (CAD) discovered in Europeans with acute CAD, chronic ischaemic heart disease (IHD) or stroke in Chinese adults.

Methods: In a nested case-control study of acute CAD (4748 cases/66,227 controls) in China Kadoorie Biobank (CKB), we compared associations of 224 SNPs for acute CAD discovered in Europeans in CARDIOGRAMplusC4D (CC4D) with acute CAD in Chinese. We compared associations of a genetic score (GS-CAD) for 224 SNPs for acute CAD with chronic IHD (n=9000), heart failure (n=1229), stroke (n=10,208) and CVD risk factors (n=70,364) and plasma proteomics (n=3904) in CKB.

Results: Overall, the strength of associations of 224 CAD SNPs in Europeans was moderately correlated with those for acute CAD in Chinese (r=0.53) and significant associations were replicated (p<0.05) for 25 SNPs. The strength of associations with acute CAD for a 1 SD higher genetic score for acute CAD in Chinese were 3-fold greater than for chronic IHD or heart failure (1.22 [1.18-1.26] vs 1.07 [1.05-1.10] vs 1.06 [1.00-1.12], respectively) and 7-fold greater than for stroke (1.03; 1.01-1.05). Higher levels of GS-CAD were strongly associated with LDL-cholesterol (LDL-C), moderately with systolic blood pressure (SBP) and weakly with body mass index (BMI), but not with proteomics.

Conclusion: The results demonstrate moderate concordance in genetic determinants for acute CAD between Europeans and Chinese that was strongly related to LDL-C, and to a lesser extent with SBP and BMI, but only weakly associated with chronic IHD or stroke.

Non-standard abbreviations and acronyms

BBJ:	Biobank Japan
BMI:	Body mass index
CC4D	CARDIoGRAMplusC4D
CKB	China Kadoorie Biobank
CDC	Center of Disease Control
CVD	Cardiovascular diseases
DBP	Diastolic blood pressure
ECG	Electrocardiogram
FDR	False discovery rate
GS	Genetic score
GS-CAD	Genetic risk score for CAD
GWAS	Genome-wide association studies
HDL-C	High-density lipoprotein cholesterol
IHD	Ischaemic heart disease
LDL-C	Low-density lipoprotein cholesterol
Mb	Megabase
MCE	Major coronary events
MI	Myocardial infarction
MR	Mendelian randomization
PheWAS	Phenome-wide association studies
PLA2G7	Lipoprotein-associated phospholipase A2
pQTLs	Protein quantitative trait loci
QC	Quality control
SBP	Systolic blood pressure
SNPs	Single nucleotide polymorphisms
SUSD2	Sushi-containing domain protein
TOPMed	Trans-Omics for Precision Medicine
WBBC	West Lake Biobank for Chinese
WHR	Waist-to-hip ratio

Lay Summary

Differences in risk of heart attack and other types of ischaemic heart disease vary between ancestries and may reflect higher levels of cigarette smoking and blood pressure or lower levels of blood lipids in Chinese than in Europeans, but whether these may also reflect differences in genetic factors has not yet been established.

In a population study of Chinese adults, we compared the strength of associations of 224 genetic markers for acute heart attack or death from ischaemic heart disease discovered in Europeans in a study of over 210,000 cases and 1 million controls with almost 4748 acute heart attack cases, 9000 chronic ischaemic heart disease cases and 10,200 stroke cases in Chinese adults in the China Kadoorie Biobank. We compared the strengths of associations of genetic markers for acute heart attack in Europeans with those for acute heart attack, chronic heart disease (IHD) or stroke in Chinese adults. The results demonstrated high levels of agreement in the strength of associations of genetic markers for heart attack between European and Chinese adults and these markers also influenced levels of low-density lipoprotein cholesterol and blood pressure. However, the genetic markers for acute heart attack were less strongly associated with other types of heart disease or stroke in Chinese adults. The findings suggest a shared genetic architecture for acute heart attack between Europeans and Chinese that is partly mediated through blood lipids and blood pressure that is specific for complete blockage of large arteries supplying the heart rather than chronic heart disease or stroke.

Introduction

Coronary artery disease (CAD) and stroke are the leading causes of morbidity and mortality worldwide.[1] The prevalence of these diseases has increased globally by about 2-fold over the last three decades.[2] CVD mortality now accounts for about 40% of all deaths in China.[3, 4] Premature deaths (<70 years) from cardiovascular disease (CVD) have declined in both Western and Chinese populations over the last few decades reflecting improvements in lifestyle and access to medical treatment to reduce levels of CVD risk factors.

The prevalence of current cigarette smoking is much higher in Chinese men than in Europeans, and mean levels of low-density lipoprotein cholesterol (LDL-C) and body mass index are lower in both men and women in China than in Europe, but little is known about the comparative importance of genetic determinants for acute CAD (i.e., non-fatal myocardial infarction or death from ischaemic heart disease [IHD]) discovered in Europeans for acute CAD in Chinese adults. [5-8]

Genome-wide association studies of CAD in European ancestry populations have reported over 241 single nucleotide polymorphisms (SNPs) that were independently associated with CAD demonstrating a polygenic genetic architecture involving multiple SNPs in Europeans each typically having only modest effects on acute CAD.[9-11] While the effects of some of these SNPs on CAD are mediated through levels of LDL-C, SBP, diabetes or adiposity, but the mechanisms for effects of other SNPs on acute CAD are not fully understood.[11-16] Likewise, the relevance of the genetic variants for acute CAD (MI and IHD death) for other non-MI IHD subtypes (i.e., chronic IHD), non-IHD cardiac outcomes or stroke has not been widely studied in Chinese adults. [10]

The different IHD subtypes vary by severity of coronary artery occlusion and reflect underlying pathology and severity of CAD. Acute coronary artery disease (CAD) (involving myocardial infarction [MI] or death from ischaemic heart disease [IHD]), also referred to as "major coronary events" in clinical trials and practice is the most serious form of acute CAD that reflects complete occlusion of one or more coronary arteries with plaque rupture and superimposed thrombosis. In contrast, the more common chronic IHD cases typically have partial occlusion of one or more coronary arteries. Acute MI is distinguished from unstable angina or chronic IHD by elevated plasma levels of troponins and characteristic ST segment changes and or pathological Q waves on serial electrocardiograms (ECG) recordings.

A previous study of the diagnostic accuracy of first hospitalized cases of MI and non-MI IHD in Chinese adults reported that the diagnostic accuracy of MI cases was 98% and non-MI IHD cases was 93% consistent with findings in Europeans.(17) Consequently, comparative studies assessing the concordance of genetic determinants for acute CAD discovered in Europe for acute CAD, chronic IHD and non-IHD cardiac diseases occurring in Chinese adults with different diet and lifestyle habits should be informative about the relevance of genetic susceptibility for acute CAD, chronic IHD, stroke and other non-IHD cardiac outcomes in China.

A recent genome-wide association study (GWAS) of CAD, involving about 180,000 CAD cases identified independent associations for 241 variants at 198 loci for CAD in Europeans.(9) Comparison of GWAS results for CAD in Europeans with those in Japanese (about 30,000 CAD cases) demonstrated consistent effect sizes for many variants, and meta-analysis of Europeans with Japanese yielded 38 additional variants for acute CAD. Previous GWAS of CAD conducted in Chinese adults also identified some shared loci for CAD between Europeans and Chinese.(10, 11) Differences in the

genetic architecture or frequency of common variants or effect modification by levels of established CAD risk factors could contribute to the differences in the incidence and severity of CAD or IHD subtypes between Chinese and European populations.[11-13] However, there have been few comparative studies of GWAS variants for acute CAD, chronic IHD and other non-IHD cardiac outcomes in Chinese and European populations and their relevance for other CVD subtypes.[14-16]

The aims of this study were to: (i) compare the concordance of associations of individual SNPs for acute CAD discovered in European ancestry populations in CARDIOGRAMplusC4D with acute CAD in Chinese; (ii) assess the concordance of associations of acute CAD SNPs discovered in Europe for acute CAD, chronic IHD, non-IHD cardiac conditions and stroke types using a genetic score combining such European ancestry SNPs for CAD (GS-CAD) in a nested case-control studies in Chinese individuals in CKB; (iii) and compare associations of fifths of GS-CAD score with levels of LDL-C, systolic blood pressure (SBP), body mass index (BMI) and plasma proteomics in controls with GWAS data in CKB.

Methods

Study population

Details of the design and methods used in the CKB study have been previously reported. [18, 19] Briefly, the CKB study involves a 12-year follow-up of population-based prospective study of 512,891 adults aged 30-79 years that were recruited from 10 regions in China between 2004 and 2008. All participants completed an interviewer-administered questionnaire that collected data on demographic and lifestyle characteristics (including smoking and alcohol consumption), medical history, and

current use of medications. GWAS data were available on about one fifth of the CKB study population involving a population subset and additional cases with MCE, stroke and other CVD subtypes and with CAD risk factors (**Figure 1**). The present report involved five separate comparisons with different subsets of the CKB study population, including: (A) associations of individual SNPs for major coronary events (MCE) that were discovered in Europeans with MCE in Chinese and a comparative study of a genetic score combining these SNPs (GS-CAD) with MCE or non-MI (chronic IHD) in CKB; (B) comparison of a GS-CAD score with stroke, heart failure or vascular death in CKB; (C) comparison of a GS-CAD score with levels of major CVD risk factors; (E) phenome-wide association study of a GS-CAD score with all available major disease outcomes in CKB (**Figure 1**).

All participants underwent a physical examination that recorded measurements of anthropometry, blood pressure (BP), and spirometry, and provided a blood sample. Ethics approval was obtained from both University of Oxford (Oxford Tropical Research Ethics Committee), the Chinese Academy of Medical Sciences, Peking University and the China National Centers for Disease Control. In addition, approvals were obtained from institutional research boards at the 10 local regions. All participants provided written informed consent for participation in this study and for their health status to be monitored by linkage with their electronic health records.

Established CVD risk factors included SBP that was measured as the mean of the last two measurements that were recorded after 5 minutes in the seated position.[20] Plasma lipid levels were measured in a subset of the population (n=18,728) using AU680 chemistry analysers (Beckman-Coulter), which used immunoassays for LDL-C and HDL-C, and immunoturbidimetric assays for total cholesterol and

triglycerides.[21] Plasma levels of 2923 proteins were measured using the Olink Explore 3072 panel (OLINK Biosciences, Uppsala, Sweden) for a subset of 1971 CAD cases and 2001 sub-cohort controls.[22, 23]

Genome wide genotyping

DNA extraction, genotyping, array design, and population structure analyses for the CKB have been previously described.[24] Two custom-designed arrays were used to genotype about 800,000 variants. Genotyping and QC of a total of 105,408 CKB DNA samples are summarized in **Figure 1**. The initial CKB array design was used to genotype 33,408 samples that had been selected for nested case-control studies of cardiovascular disease and COPD. On the basis of disease follow-up to January 1, 2014, this initial genotyping included all incident cases of intracerebral haemorrhage (ICH; n= 5,020), subarachnoid haemorrhage (SAH; n=455), and fatal IHD (n=753); randomly selected incident cases of ischaemic stroke (IS; n=5,662), MI (n=1,008), and COPD (n=5,376); participants with no cardiovascular events (n = 10,038) at time of this study. The updated array design was then used to genotype a further 72,000 population samples (thereafter referred to as “population-subset”), including all available additional cases of ICH (n=602), SAH (n=46), MI (n=1,028), and fatal IHD (n=163) that had not previously been genotyped. After exclusion of samples on the basis of call rate, excess hetero- or homozygosity, sex mismatch, XY aneuploidy, and non-East-Asian ancestry, genome-wide genotype data were available on 100,706 participants. Using ~532,000 variants that passed quality control for both array versions, imputation into the TOP Med and WBBC reference panels[22] yielded ~50 million variants with an info score >0.3. Genetic analyses used genotypes extracted as dosages of the minor allele according to the imputed probability of each genotype.

The details of national and regional principal components derivation for CKB were previously published.[25]

CAD, stroke and other vascular diseases

Disease outcomes were obtained by electronic linkage via a unique national identity number to death and disease registries (CAD, stroke, cancer and diabetes) and to the national health insurance system which records all in-patient hospital events. Data on all incident disease outcomes were integrated and standardised using the International Classification of Diseases 10th Edition (ICD-10) coded incident disease outcomes. Incident cases of CAD were defined as follows (**Table S1**): (i) acute MI (ICD-10 codes: I21-I23); (ii) major coronary events (MCE), defined as either acute MI (I21-I23) or death from IHD (underlying cause of death, I20-I25); and (iii) IHD mortality (defined as any mortality event with ICD-10 I20-I25). In addition, incident cases of eight other cardiovascular disease (CVD) outcomes were defined as follows: ischaemic stroke (IS: I63), intra-cerebral haemorrhage (ICH: I61), all stroke, (I60-61 and I63-64), vascular death (fatal I00-I99 and R96); chronic IHD (I25); hypertensive heart disease (I11), pulmonary heart disease (I26-I28) and heart failure (I50).

Logistic regression was used to assess associations of CAD SNPs with CAD or other IHD types, cases with MCE (n=4748) and chronic IHD (n=9000) were compared with controls within the population subset (n=62,107) (**Figure 1A**). Likewise, analyses of GS-CAD with stroke, CVD death and heart failure, stroke cases (n=10208), vascular deaths (n=6375) and HF cases (n=1229) were compared with 62,107 controls in the population subset (**Figure 1B**). Linear regression was used to assess associations of GS-CAD with blood pressure (n=70,364), BMI (n=70,363) and LDL-C (n=5764), respectively. PheWAS analyses of GS-CAD with all common diseases in 62,107 control participants from the population subset (**Figure 1C**) and logistic regression was

used to assess associations with all common diseases, involving 200 or more first incident cases with GWAS data (**Figure 1D**). Linear regression was used to assess associations of GS-CAD with 2923 proteins in 1962 CAD cases and 1942 controls (**Figure 1E**).

Statistical analyses

Replication of associations of European CAD variants with CVD in Chinese

Logistic regression models were used to assess associations with each CVD subtype in each region, after adjusting for age at baseline, age squared, ever smoking (defined as: current or former regular smoking), sex, and the appropriate number of regional principal components per region. The region-specific results were then combined using an inverse-variance weighted (IVW) meta-analysis. Group-specific variances and 95%CI were used for categorical variables to enable comparisons between any two categories (including the reference category).[26, 27] After aligning effect alleles, Pearson correlation coefficients were used to compare the coefficients for CAD outcomes and minor allele frequencies across ancestries. For assessment of replication, analyses were limited to SNPs with an allele frequency >0.01 and info score>0.8 in the CKB. SNPs with a minor allele frequency <0.01 in East Asians and indels were excluded.

Construction of a genetic score for CAD

Figure 1 shows a flow diagram of the study design and number of participants in genetic analyses of CAD SNPs with major coronary events and chronic IHD, other CVD, CVD risk factors and phenome-wide association analysis (PheWAS) with all common major disease outcomes in CKB. **Figure S1** shows the number of individual SNPs and genetic score combining these SNPs in CKB. Genetic scores (GS) for CAD

were estimated using the dosages of the 224 European ancestry genome-wide significant SNPs in CC4D that were available in CKB). The weighted GS-CAD score was computed using weights based on the log ORs for CAD in European ancestry participants in CC4D using the “riskScore” function from the PredictABEL R package,[28] and standardized using a Z-score (GS- GS mean)/(GS standard deviation). Participants were assigned to fifths of the GS-CAD score to assess the shape of the association with CVD outcomes.

Associations of European GS-CAD with CAD, stroke and other CVD outcomes in Chinese

Analyses of associations with CVD risk factors were limited to the population sub-cohort for blood pressure (n=70,364) and BMI (n=70,363) (**Figure 1**). The associations with LDL-C were performed in a subset of 5764 controls with no history of CAD or stroke. The associations of fifths of a GS-CAD score with CAD and other CVD outcomes were assessed region-specifically via logistic regression after adjusting for age at baseline, age squared, ever smoking, sex, and regional principal components. Likewise, the associations of fifths of GS-CAD with levels of CAD risk were assessed using region-specific linear regression models, after adjusting for age at baseline, age squared, ever smoking, sex, and regional principal components.

Phenome-wide association analyses

A phenome-wide association analysis (PheWAS) was conducted using 117 disease phecodes, where each had 200 or more cases in the population subset of genotyped individuals in CKB. Phecodes are curated groupings of ICD codes that were designed

to capture all available ICD diagnosis codes per condition, focusing on conditions relevant for GWAS analyses.[29, 30] Phecodes mappings and definition are maintained by the Center for Precision Medicine at Vanderbilt University Medical Center (https://www.phewascatalog.org/phecodes).[29, 30] Logistic regression analyses were conducted for each phenotype, adjusted for age at baseline, age squared, sex, region, smoking and the first 11 national principal components. A Bonferonni significance level was estimated after taking account the number of phenotypes.

Proteome-wide analyses

A proteome-wide analysis was conducted in 3977 participants with available data on 2923 proteins in CKB that were assayed using the Olink Explore panel to assess associations of GS-CAD with plasma proteomics (**Figure 1**). Linear regression analyses were used for each protein, adjusted for age at baseline, age squared, sex, region, smoking, plate ID and the first national 11 principal components. A 5% false discovery rate (FDR) correction was used, and a Bonferonni-threshold was also estimated based on the number of protein associations tested.

Cis-protein quantitative trait loci (cis-pQTLs), defined as the cis-variants associated with plasma levels of 2923 proteins at GWAS significance level were used as instrumental variables for Mendelian randomization (MR) analyses.[31] Region-specific logistic regression models were used to investigate the associations of these two pQTLs with MCE, after adjustment for age, age squared, sex, ever smoking, and the appropriate number of regional PCs. The results were subsequently combined using inverse-variance weighted meta-analysis using the Wald ratio.[31] Directionality of the associations was assessed using Steiger filtering.[32] In addition, one sample MR was

conducted as a sensitivity analysis, limiting the sample to the proteomics subset, using the OneSampleMR package.[33]

Results

The baseline characteristics of cases classified by major coronary events (MCE) or chronic IHD and controls are shown in **Table 1**. Overall, compared with controls, cases with MCE were older, and more likely to be male, but similar proportions of cases and controls lived in urban and rural areas. The proportions of ever-smokers in Chinese were much greater in male cases and male controls that female cases or female controls. MCE cases had a higher prevalence of diabetes or hypertension than controls. Likewise, MCE cases had higher mean levels of SBP and DBP than controls, respectively. Conversely, mean levels of BMI did not differ between MCE cases and controls.

Cross-ancestry comparisons

Details of the SNPs selected for analysis in the CKB are provided in **Figure S1**. A total of 224 SNPs that were significantly associated with MCE in CC4D were available in CKB. Overall, the effect estimates for the 224 CC4D SNPs were positively correlated with CAD in both ancestries (correlation coefficient=0.53, Chinese in CKB and Europeans in CC4D: **Figure 2A**). A total of 25 European SNPs were significantly associated with MCE ($p<0.05$) and the associations of these identical SNPs with acute CAD in Chinese and Europeans were directionally consistent in both populations (**Figure 2B, Table S2**). Among these SNPs for CAD in CKB, 11 were previously associated with blood pressure, 7 with lipids, 3 with diabetes and 3 with BMI.[9]

Associations of GS-CAD with CAD, stroke and other CVD subtypes

Analyses of fifths of GS-CAD genetic score with MCE, MI and chronic IHD indicated linear positive associations of this genetic score with different IHD types, but the strength of the associations (OR, 95%CI) with MCE were 3-fold greater than for chronic IHD (**Figure 3**: OR and 95%CI for MCE:1.22 [95%CI:1.18-1.26]; MI:1.25 [1.20-1.29]; and chronic IHD:1.07 [1.05-1.10] per 1 SD higher GS-CAD, respectively). The effects of 1 SD higher GS-CAD for 11 different CVD outcomes were examined in CKB (**Figure 4**). A 1 SD higher level of GS-CAD score was associated with a higher risk of MCE, MI, fatal IHD, vascular death and chronic IHD. However, a 1 SD higher level of GS-CAD was only weakly associated with a higher risk of any stroke (chiefly IS, but not with ICH) or with heart failure, but not at all with hypertensive heart disease or pulmonary heart disease. The top 3 variants most strongly associated with higher risks of major coronary events (MCE) in Chinese included *APOE* and two variants for *CDKN2B-AS1* and the top 3 variants inversely associated with MCE included *PRDM16, LPA*, and *ABHD2,* respectively, consistent with the findings in Europeans. Overall, the OR (95%CI) for MCE for a genetic score for all 224 SNPs was 1.22 (1.18-1.26) and for the subset of 25 variants that were successfully replicated at $p<0.05$ in CKB was 1.21 (1.17-1.25) (**Table S3**).

Associations of GS-CAD with CVD risk factors

Figure 5 shows associations of fifths of a genetic risk score for acute CAD (GS-CAD) with mean levels of CVD risk factors including: (A) SBP; (B) DBP, (C) BMI and (D) LDL-C, respectively. The findings indicated linear and strong positive associations of fifths of GS-CAD genetic score with higher levels LDL-C, but only

moderate associations with levels of SBP, and to a lesser extent with DBP, but no associations with BMI (**Figure 5**).

PheWAS of GS-CAD score with other CVD and non-CVD disease outcomes

A total of 15 groups of diseases, involving 117 phenotypes were tested in phenome-wide analyses for their associations with a 1 SD higher GS-CAD genetic score (**Figure S2**). Three phenotypes were significantly associated with the GS after correction for multiple testing: MI (OR: 1.26; 95%CI: 1.20-1.33), coronary atherosclerosis (OR: 1.10; 95%CI: 1.07-1.13) and occlusion of cerebral arteries (OR: 1.04; 95%CI: 1.02-1.08), respectively demonstrating the specificity for these associations.

Associations of GS-CAD score with plasma proteomics

Analyses of associations of GHS-CAD with plasma levels of 2923 proteins indicated that a 1 SD higher score was associated with higher levels of two proteins after correction for multiple testing: Lipoprotein-associated phospholipase A2 (PLA2G7: coefficient 0.05; 95%CI: 0.03-0.07) and Sushi-containing domain protein (SUSD2: coefficient: -0.02; 95%CI: -0.01- -0.04) (**Figure S3**). However, there was no evidence of an association with SUSD2 in a sensitivity analysis using a modified score that excluded variants located within 1 megabase of the annotated gene region for each protein. Furthermore, two cis-PQTLs were identified, one for each of the two proteins, (PLA2G7) and SUSD2 (**Table S4**), but there was no evidence for a causal role for these proteins for CAD risk in Chinese using either one-sample or two-sample MR analyses with each pQTL as an instrumental variable for their respective proteins (**Figure S4**).

Discussion

This study assessed associations of 224 SNPs for acute CAD that were discovered in Europeans with acute CAD in Chinese adults and confirmed a moderate concordance of genetic determinants between these ancestry populations. Despite a relatively limited number of SNPs (n=25) being nominally significant for associations with CAD (at p<0.05) in CKB, a weighted GS-CAD score using weights derived from a European population was moderately strongly associated with acute CAD in Chinese, but was only weakly associated with chronic IHD outcomes or vascular mortality in Chinese adults. Individuals in the top vs bottom fifths of GS-CAD were associated with about a 2-fold higher risk of MI, but with only 20% higher risk of chronic IHD. The GS-CAD score was also weakly positively associated with a higher risk of IS and was unrelated to HS.

The GS-CAD score was strongly associated with plasma levels of LDL-C, and to a lesser extent with SBP, but not with BMI. A PheWAS analysis for 117 phenotypes in Chinese indicated that the GS-CAD score was significantly associated with higher risk of MI, coronary atherosclerosis and occlusion of the cerebral arteries, respectively. Analysis of associations of GS-CAD with 2923 proteins demonstrated associations with PLA2G7 and SUSD2. However, only PLA2G7 remained associated with the GS when variants within 1 Mb of the gene location (variants within the cis-region) were removed. Mendelian randomization analyses suggested that neither of the associations of these two proteins with CAD were causal.

The number of CVD deaths and proportions of total deaths due to CVD has increased exponentially in China compared with Western countries in recent decades.[34] Several studies have reported a greater disease severity for CAD in Asians than in Europeans.[35] Heritability of CAD has been estimated to be ~40-60% in twin studies

[15] (with the independent variants for IHD identified in the present study explaining ~36% of heritability), it is likely that most of the differences in acute CAD incidence and severity between European and East Asians reflects differences in diet and lifestyle and medical treatment.[9] Comparing the associations of genetic variants for CAD in different genetic ancestries can help to understand the genetic architecture for CAD in diverse populations. Moreover, as most of GWAS studies have been conducted in European populations, the generalizability of the resulting GWAS or GS-CAD genetic score for CAD in other ancestries may require validation in future cycles of CARDIoGRAMplusC4D that are expected to include a greater number of cases from East Asian populations.[36] A previous study of associations of European ancestry GS-CAD genetic score with CAD in non-European participants in UK-Biobank reported a good correlation across most included ancestries, except for Africans, but the analyses were based on very small numbers of CAD cases from non-European ancestry populations.[37] The percentage of replicated SNPs and correlations between effect sizes between European and Chinese in the present study were consistent with previous reports comparing European and Japanese ancestry populations.[9]

Moreover, a European ancestry GS-CAD score was associated with a higher risk of coronary artery atherosclerosis and cerebral artery occlusion in a phenome-wide analysis. The results are consistent with a shared aetiology for atherosclerotic diseases between Chinese and European ancestries. With the exception of LDL-C and SBP, the GS-CAD was not strongly associated with other CAD risk factors and the results are consistent with previous reports on the shared importance of blood lipids between Europeans and East Asian ancestries and with stronger associations with acute than chronic IHD.[38, 39] Thus, LDL-C and SBP are major determinants of the shared genetic aetiology of CAD in both populations. The results also suggest that

differences in CAD severity between Europeans and Chinese ancestries may reflect differences in levels of SBP and LDL-C in these populations.

In a separate comparative report of GWAS associations of Lp(a)-related variants with Lp(a) in Chinese vs Europeans, we demonstrated only a modest overlap of Lp(a)-related variants with Lp(a) levels in both populations with only a single SNP rs73596816 being strongly associated with Lp(a) levels in both Chinese and Europeans.[40] In contrast, the two lead SNPs most strongly associated with Lp(a) levels and MI in Europeans[41] were unrelated to Lp(a) or MI in Chinese with rs10455872 being very rare and rs3798120 SNP having only a minimal effect on Lp(a) levels in Chinese. However, our previous study reported that the rs73596816 SNP was strongly related to Lp(a) levels and risk of MI in both Chinese and East Asians populations. The present report confirmed the comparable strength of associations of rs73596816 with risk of MI in both ancestry populations (OR [95%CI]: 1.13 [1.02-1.25] vs 1.11 [1.08-1.14] in Chinese vs Europeans, respectively).[40] Despite differences in the genetic architecture of the *LPA* locus between Chinese and Europeans, both the present and previous reports demonstrated that equivalent genetically-predicted differences in Lp(a) levels had comparable effects on MI in both ancestry populations.[40,41]

The present analysis demonstrated only a weak positive association of the GS-CAD score with ischaemic stroke or occlusion of cerebral arteries in a PheWAS analysis in addition to MI and coronary atherosclerosis. Hence, the results of this study add to a growing body of evidence highlighting the shared biological pathways between acute CAD and large artery ischaemic stroke and provide further support for lowering LDL-C and SBP for prevention of occlusive atherosclerotic vascular diseases.

In contrast, we found little evidence of shared genetic pathways between CAD with hypertensive or pulmonary heart diseases. Previous studies suggested some shared heritability between CAD and ischaemic stroke,[42] and a significant overlap of genetic variants between ischaemic stroke and CAD.[43] Importantly, six known CAD risk loci, including 9p21 (the locus with the smallest p value in our replication analysis), were previously associated with either ischaemic or large artery stroke.[44] Despite the 9p21 locus being one of the most studied variants for IHD, its mechanism of action is not yet fully understood. Importantly, the 9p21 locus has been previously associated with atherosclerosis, but not with thrombosis or plaque rupture.[45] The findings suggesting that 9p21 has a role in both CAD and acute IS may be mediated through effects on atherosclerosis, contributing to a shared genetic architecture of CAD in both Chinese and European populations.

The chief strengths of the present study include the large number of cases of CAD, stroke and other CVD outcomes and CVD risk factors from a single study in Chinese adults recruited from 10 diverse geographical regions in China.[35, 46] However, the study also had several limitations. First, the number of cases of MI, MCE, or IHD were insufficient to have sufficient statistical power to replicate associations with lower frequency variants or variants with smaller effect sizes. Second, we excluded phenotypes with less than 200 participants in our PheWAS analysis, and, hence, could have missed some phenotypes associated with GS-CAD score. [30] The sample size for proteomics analysis may be too small to detect associations with low frequency variants or variants associated with small effects on risk of CAD. An alternative explanation for the null associations of CAD-GS with proteomics may suggest that CAD SNPs may be involved in initiation of atherosclerosis of the coronary arteries, but

plasma proteomics may more closely related to determinants of progression of atherosclerosis.

Conclusions

Overall, this study demonstrated a moderate concordance of genetic determinants of acute CAD between European and Chinese adults and a GS-CAD genetic score combining all such variants was moderately strongly associated with acute CAD in Chinese adults. However, the strength of association of this GS-CAD genetic score with acute CAD was 3-fold stronger than for chronic IHD or heart failure and 7-fold stronger than for stroke. The GS-CAD score was also strongly associated with levels of LDL-C or SBP, but only modestly associated with BMI. The results highlighted a shared genetic architecture of acute CAD between Europeans and Chinese that is partly mediated through LDL-C and SBP and provides support for lowering mean levels of these risk factors for prevention of CAD in the Chinese population in addition to further research on strategies to lower levels of Lp(a) or inflammatory markers for prevention of acute CAD in Chinese as in European populations.

Acknowledgments: The chief acknowledgment is to the participants, the project staff, the China CDC and its regional offices for assisting with the fieldwork and long-term follow up for mortality and morbidity.

Funding: The CKB baseline survey and the first re-survey were supported by the Kadoorie Charitable Foundation in Hong Kong. The long-term follow-up has been supported by Wellcome grants to Oxford University (212946/Z/18/Z, 202922/Z/16/Z, 104085/Z/14/Z, 088158/Z/09/Z) and grants from the National Natural Science Foundation of China (82192900, 82192901, 82192904，82388102) and the Noncommunicable Chronic Diseases-National Science and Technology Major Project (2023ZD0510100).The UK Medical Research Council (MC_UU_00017/1,MC_UU_12026/2, MC_U137686851), Cancer Research UK (C16077/A29186; C500/A16896) and the British Heart Foundation (CH/1996001/9454), provide core funding to the Clinical Trial Service Unit and Epidemiological Studies Unit at Oxford University for the project.

Conflict of interest/Competing interests: None

Ethics approval: The China Kadoorie Biobank (CKB) complies with all required ethical standards for medical research on human subjects. Ethical approvals were obtained and been maintained by the relevant institutional ethical research committees in the UK and China.

Consent to participate/publication: All participants provided written informed consent.

Data Access Statement: The China Kadoorie Biobank (CKB) is a global resource for the investigation of lifestyle, environmental, blood biochemical and genetic factors as determinants of common diseases. The CKB study group is committed to making the cohort data available to the scientific community in China, the UK and worldwide to advance knowledge about the causes, prevention and treatment of disease. Details of the data that are currently available to open access users and how to access these are shown on: http://www.ckbiobank.org/site/data-access. Additional information on the data access underlying these results can be provided by contacting ckbaccess@ndph.ox.ac.uk. For the purpose of Open Access, the author has applied a CC-BY public copyright licence to any Author Accepted Manuscript version arising from this submission. Data is shared for no charge to researchers in China, and to all other researchers for £2500 to cover costs.

Code availability: Details of analyses of summary level data and intermediate files are provided on https://pheweb.ckbiobank.org and R codes used for analyses are deposited using GitHub with URLs provided in accepted reports for publications to comply with journal requirements.

Disclosures: None.

Author Contributions: AE conducted statistical analyses and wrote the first draft of the report. KL identified genetic instruments for the study and SM conducted the initial statistical analyses. RC and DAB designed the study, supervised analyses and revised the report. PS generated figures. CQ supervised local data acquisition and CY, JL, DS, PP, LL, FL and ZC coordinated data acquisition centrally in China for the baseline and long-term follow-up. DA supervised data curation. IYM and RGW supervised the genome-wide analyses. All authors approved the final version of the report for publication.

References

1. Vaduganathan M, Mensah GA, Turco JV, Fuster V, Roth GA. The Global Burden of Cardiovascular Diseases and Risk: A Compass for Future Health. J Am Coll Cardiol. 2022;80(25):2361-71.
2. Roth GA, Mensah GA, Johnson CO, Addolorato G, Ammirati E, Baddour LM, et al. Global Burden of Cardiovascular Diseases and Risk Factors, 1990-2019: Update From the GBD 2019 Study. J Am Coll Cardiol. 2020;76(25):2982-3021.
3. Li JJ, Liu HH, Li S. Landscape of cardiometabolic risk factors in Chinese population: a narrative review. Cardiovasc Diabetol. 2022;21(1):113.
4. Rehman S, Rehman E, Mumtaz A, Jianglin Z. Cardiovascular Disease Mortality and Potential Risk Factor in China: A Multi-Dimensional Assessment by a Grey Relational Approach. Int J Public Health. 2022;67:1604599.
5. Doi T, Langsted A, Nordestgaard BG. Lipoproteins, Cholesterol, and Atherosclerotic Cardiovascular Disease in East Asians and Europeans. J Atheroscler Thromb. 2023;30(11):1525-46.
6. Oakkar EE, Stevens J, Truesdale KP, Cai J. BMI and all-cause mortality among Chinese and Caucasians: the People's Republic of China and the Atherosclerosis Risk in Communities Studies. Asia Pac J Clin Nutr. 2015;24(3):472-9.
7. Lewington S, Lacey B, Clarke R, Guo Y, Kong XL, Yang L, et al. The Burden of Hypertension and Associated Risk for Cardiovascular Mortality in China. JAMA Intern Med. 2016;176(4):524-32.
8. Organization WH. WHO Global Report on Trends in Prevalence of Tobacco Use 2000-2025. 2021.
9. Aragam KG, Jiang T, Goel A, Kanoni S, Wolford BN, Atri DS, et al. Discovery and systematic characterization of risk variants and genes for coronary artery disease in over a million participants. Nat Genet. 2022;54(12):1803-15.
10. Lu X, Wang L, Chen S, He L, Yang X, Shi Y, et al. Genome-wide association study in Han Chinese identifies four new susceptibility loci for coronary artery disease. Nat Genet. 2012;44(8):890-4.
11. Assimes TL, Roberts R. Genetics: Implications for Prevention and Management of Coronary Artery Disease. J Am Coll Cardiol. 2016;68(25):2797-818.
12. Dandona S, Stewart AF, Chen L, Williams K, So D, O'Brien E, et al. Gene dosage of the common variant 9p21 predicts severity of coronary artery disease. J Am Coll Cardiol. 2010;56(6):479-86.
13. Jing J, Su L, Zeng Y, Tang X, Wei J, Wang L, et al. Variants in 9p21 Predicts Severity of Coronary Artery Disease in a Chinese Han Population. Ann Hum Genet. 2016;80(5):274-81.
14. Ralapanawa U, Sivakanesan R. Epidemiology and the Magnitude of Coronary Artery Disease and Acute Coronary Syndrome: A Narrative Review. J Epidemiol Glob Health. 2021;11(2):169-77.
15. Zdravkovic S, Wienke A, Pedersen NL, Marenberg ME, Yashin AI, De Faire U. Heritability of death from coronary heart disease: a 36-year follow-up of 20 966 Swedish twins. J Intern Med. 2002;252(3):247-54.
16. Tcheandjieu C, Zhu X, Hilliard AT, Clarke SL, Napolioni V, Ma S, et al. Large-scale genome-wide association study of coronary artery disease in genetically diverse populations. Nat Med. 2022;28(8):1679-92.
17. Turnbull I, Clarke R, Wright N, Sun D, Kaartsonaki C, Pei P, Yu C et al. Diagnostic accuracy, treatment and prognosis of myocardial infarction: an 11-year follow-up of a community-based cohort of 0.5 million Chinese adults. (Submitted)
18. Chen Z, Chen J, Collins R, Guo Y, Peto R, Wu F, et al. China Kadoorie Biobank of 0.5 million people: survey methods, baseline characteristics and long-term follow-up. Int J Epidemiol. 2011;40(6):1652-66.

19. Walters RG, Millwood IY, Lin K, Schmidt Valle D, McDonnell P, Hacker A, et al. Genotyping and population characteristics of the China Kadoorie Biobank. Cell Genomics. 2023;3(8):100361.
20. Clarke R, Wright N, Walters R, Gan W, Guo Y, Millwood IY, et al. Genetically Predicted Differences in Systolic Blood Pressure and Risk of Cardiovascular and Noncardiovascular Diseases: A Mendelian Randomization Study in Chinese Adults. Hypertension. 2023;80(3):566-76.
21. Sun L, Clarke R, Bennett D, Guo Y, Walters RG, Hill M, et al. Causal associations of blood lipids with risk of ischaemic stroke and intracerebral haemorrhage in Chinese adults. Nat Med. 2019;25(4):569-74.
22. Said S, Pozarickij A, Lin K, Morris S, Kartsonaki C, Wright N, et al. Ancestry diversity in the genetic determinants of the human plasma proteome and associated new drug targets. 2023.
23. Mazidi M, Wright N, Yao P, Kartsonaki C, Millwood IY, Fry H, et al. Plasma Proteomics to Identify Drug Targets for Ischaemic Heart Disease. J Am Coll Cardiol. 2023;82(20): 1906-20.
24. Walters RG, Millwood IY, Lin K, Valle DS, McDonnell P, Hacker A, et al. Genotyping and population structure of the China Kadoorie Biobank. medRxiv. 2022:2022.05.02.22274487.
25. Walters RG, Millwood IY, Lin K, Schmidt Valle D, McDonnell P, Hacker A, et al. Genotyping and population characteristics of the China Kadoorie Biobank. Cell Genom. 2023;3(8):100361.
26. Easton DF, Peto J, Babiker AG. Floating absolute risk: an alternative to relative risk in survival and case-control analysis avoiding an arbitrary reference group. Stat Med. 1991;10(7):1025-35.
27. Iona A, Bragg F, Guo Y, Yang L, Chen Y, Pei P, et al. Adiposity and risks of vascular and non-vascular mortality among Chinese adults with type 2 diabetes: a 10-year prospective study. BMJ Open Diabetes Res Care. 2022;10(1).
28. Kundu S, Aulchenko YS, van Duijn CM, Janssens AC. PredictABEL: an R package for the assessment of risk prediction models. Eur J Epidemiol. 2011;26(4):261-4.
29. Verma A, Bradford Y, Dudek S, Lucas AM, Verma SS, Pendergrass SA, et al. A simulation study investigating power estimates in phenome-wide association studies. BMC Bioinformatics. 2018;19(1):120.
30. Bastarache L. Using Phecodes for Research with the Electronic Health Record: From PheWAS to PheRS. Annu Rev Biomed Data Sci. 2021;4:1-19.
31. Hemani G, Zheng J, Elsworth B, Wade KH, Haberland V, Baird D, et al. The MR-Base platform supports systematic causal inference across the human phenome. Elife. 2018;7.
32. Hemani G, Tilling K, Davey Smith G. Orienting the causal relationship between imprecisely measured traits using GWAS summary data. PLoS Genet. 2017;13(11):e1007081.
33. Palmer T, Spiller, W. and Sanderson, E. OneSampleMR - R package for one-sample Mendelian randomization and instrumental variable analyses. 2021.
34. World Health Organization. Regional Office for the Western P. People's Republic of China health system review. Manila: WHO Regional Office for the Western Pacific; 2015 2015.
35. Gijsberts CM, Seneviratna A, de Carvalho LP, den Ruijter HM, Vidanapthirana P, Sorokin V, et al. Ethnicity Modifies Associations between Cardiovascular Risk Factors and Disease Severity in Parallel Dutch and Singapore Coronary Cohorts. PLoS One. 2015;10(7):e0132278.
36. O'Sullivan JW, Raghavan S, Marquez-Luna C, Luzum JA, Damrauer SM, Ashley EA, et al. Polygenic Risk Scores for Cardiovascular Disease: A Scientific Statement From the American Heart Association. Circulation 2022;146(8):e93-e118.

37. Mars N, Kerminen S, Feng YA, Kanai M, Lall K, Thomas LF, et al. Genome-wide risk prediction of common diseases across ancestries in one million people. Cell Genom. 2022;2(4):None.
38. Kuchenbaecker K, Telkar N, Reiker T, Walters RG, Lin K, Eriksson A, et al. The transferability of lipid loci across African, Asian and European cohorts. Nat Commun. 2019;10(1):4330.
39. Kilpelainen TO, Bentley AR, Noordam R, Sung YJ, Schwander K, Winkler TW, et al. Multi-ancestry study of blood lipid levels identifies four loci interacting with physical activity. Nat Commun. 2019;10(1):376.
40. Clarke R, Wright N, Lin K, Yu C, Walters RG, Lv J, Hill M, Kartsonaki C, Millwood IY, Bennett DA, Avery D, Yang L, Chen Y, Du H, Sherliker P, Yang X, Sun D, Li L, Qu C, Marcovina S, Collins R, Chen Z, Parish S; China Kadoorie Biobank Collaborative Group.Circulation. 2025; 151: 1699-1711.
41. Clarke R, Peden JF, Hopewell JC, et al. Genetic variants associated with Lp(a) lipoprotein level and coronary disease. N Engl J Med 2009; 361: 2518-2528.
42. Banerjee A, Lim CC, Silver LE, Welch SJ, Banning AP, Rothwell PM. Familial history of stroke is associated with acute coronary syndromes in women. Circ Cardiovasc Genet. 2011;4(1):9-15.
43. Dichgans M, Malik R, Konig IR, Rosand J, Clarke R, Gretarsdottir S, et al. Shared genetic susceptibility to ischaemic stroke and coronary artery disease: a genome-wide analysis of common variants. Stroke. 2014;45(1):24-36.
44. Kessler T, Erdmann J, Dichgans M, Schunkert H. Shared genetic aetiology of coronary artery disease and atherosclerotic stroke - 2015. Curr Atheroscler Rep. 2015;17(4):498.
45. Chan K, Patel RS, Newcombe P, Nelson CP, Qasim A, Epstein SE, et al. Association between the chromosome 9p21 locus and angiographic coronary artery disease burden: a collaborative meta-analysis. J Am Coll Cardiol. 2013;61(9):957-70.
46. Hou L, Chen S, Yu H, Lu X, Chen J, Wang L, et al. Associations of PLA2G7 gene polymorphisms with plasma lipoprotein-associated phospholipase A2 activity and coronary artery disease in a Chinese Han population: the Beijing atherosclerosis study. Hum Genet. 2009;125(1):11-20.

Figure legends

Figure 1: Flow diagram of study design and numbers of participants in genetic analyses of CAD SNPs with: (A) Major Coronary Events and chronic IHD, (B) other CVD, (C) CVD risk factors, (D) PheWAS with 117 diseases and (E) plasma proteomics in Chinese adults.

Figure 2: Odds ratio (OR) and 95% CI of (A) major coronary events in Chinese for 224 individual CAD SNPs discovered in Europe; and (B) Odds ratio (OR) and 95% CI of major coronary events in Chinese for a subset of 25 SNPs that were replicated in Chinese.

Figure 3: Odds ratios (OR) and (95%CI) of (A) major coronary events, (B) myocardial infarction or (C) chronic IHD in Chinese for fifths of a genetic score (GS-CAD) for 224 CAD SNPs discovered in Europeans.

Figure 4: Odds ratios (OR) and (95%CI) of CAD, stroke, and other vascular diseases in Chinese for a 1 SD higher genetic score for 224 CAD SNPs discovered in Europeans.

Figure 5: Associations of fifths of a genetic score for 224 CAD SNPs discovered in Europeans with blood pressure, body mass index and LDL-cholesterol in Chinese. Values shown are line plots for 1 SD higher levels of systolic blood pressure (SBP), diastolic blood pressure (DBP), body mass index (BMI) and LDL-cholesterol (LDL-C) versus mean standardised genetic score (GS-CAD) per quintile. The number of participants included in each quintile is indicated below each data point.

Table 1: Baseline characteristics of participants with acute and chronic coronary artery disease and controls

Mean (SD) or %	Major Coronary Events (n=4748)	Chronic Ischaemic Heart disease (n=9000)	Controls (n=62107)
**Demographic factors**			
Age, mean (SD), yr	62.2 (9.6)	58.9 (9.7)	51.1 (10.4)
Female, %	43.1	61.8	59.3
Urban, %	42.0	56.5	42.8
**Lifestyle factors**			
Male ever-smokers, %	78.0	75.3	74.4
Female ever-smokers, %	9.5	7.4	3.0
Male ever-drinkers, %	41.8	46.8	49.7
Female ever-drinkers, %	4.1	6.0	4.2
**Medical history**			
Diabetes*, %	16.6	11.1	5.0
Hypertension*, %	26.4	24.0	8.9
**Clinical measurements**,			
SBP, mean (SD), mmHg	146 (25)	138(24)	131 (21)
DBP, mean (SD), mmHg	81 (13)	80 (12)	78 (11)
BMI, mean (SD), Kg/m^2	23.8 (3.9)	24.6 (3.9)	23.6 (3.4)

*Self-reported doctor-diagnosed diabetes or diabetes identified by blood glucose at baseline; MCE: Major coronary events, chronic IHD: chronic ischaemic heart disease; SBP: systolic blood pressure; DBP: diastolic blood pressure; BMI: body mass index

Figure 1: Flow diagram of study design and numbers of participants in genetic analyses of CAD SNPs with: (A) Major Coronary Events and chronic IHD, (B) other CVD, (C) CVD risk factors, (D) PheWAS with 117 diseases and (E) plasma proteomics in Chinese adults.

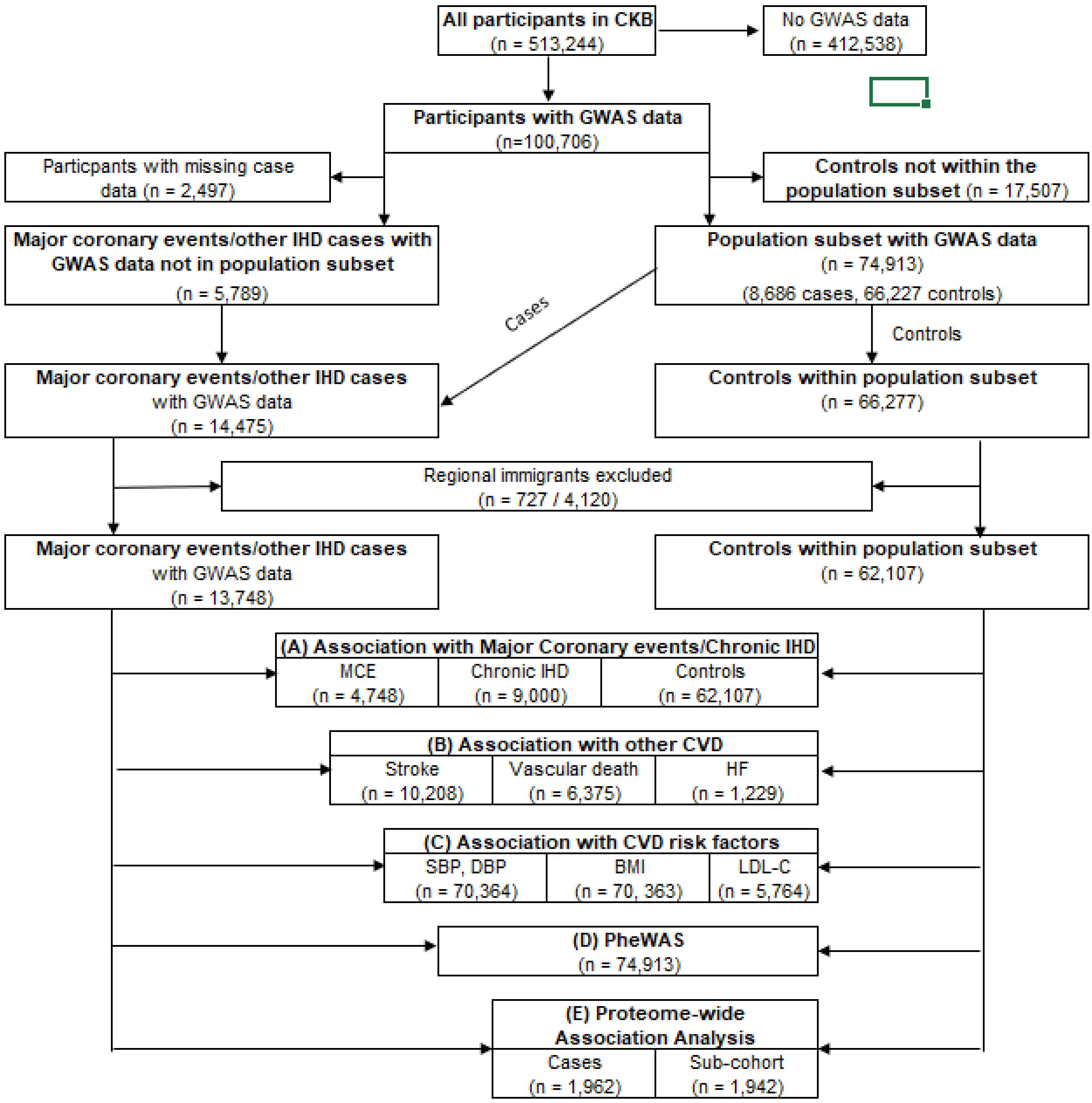

Figure 2: Associations of 224 European CAD SNPs with (A) CAD in Chinese and Europeans (B) and associations with CAD for a subset of 25 SNPs that were successfully replicated in CKB.

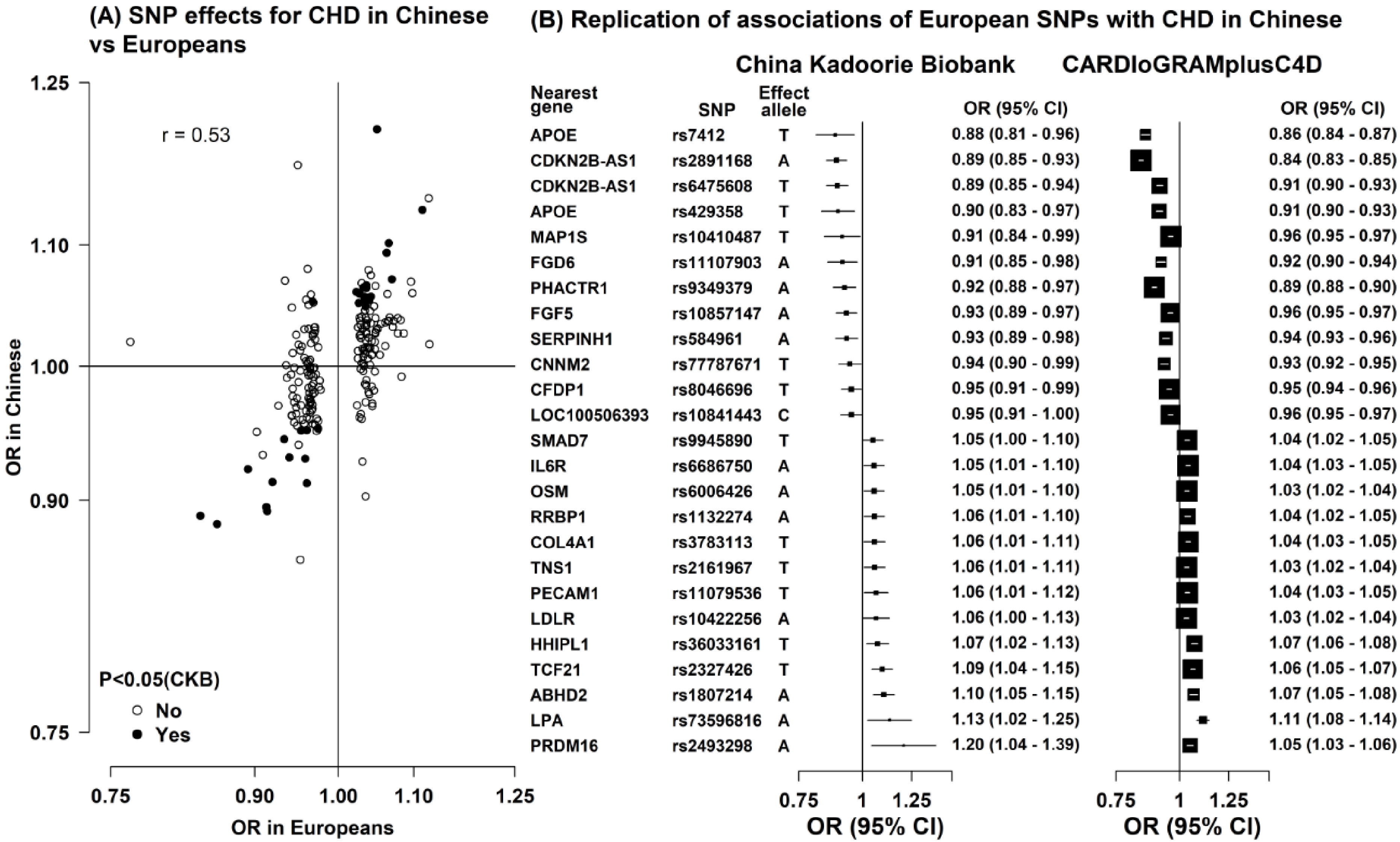

Figure 3: Odds ratios (OR) and (95%CI) of (A) major coronary events, (B) myocardial infarction or (C) chronic IHD in Chinese for fifths of a genetic score (GS-CAD) for 224 CAD SNPs discovered in Europeans.

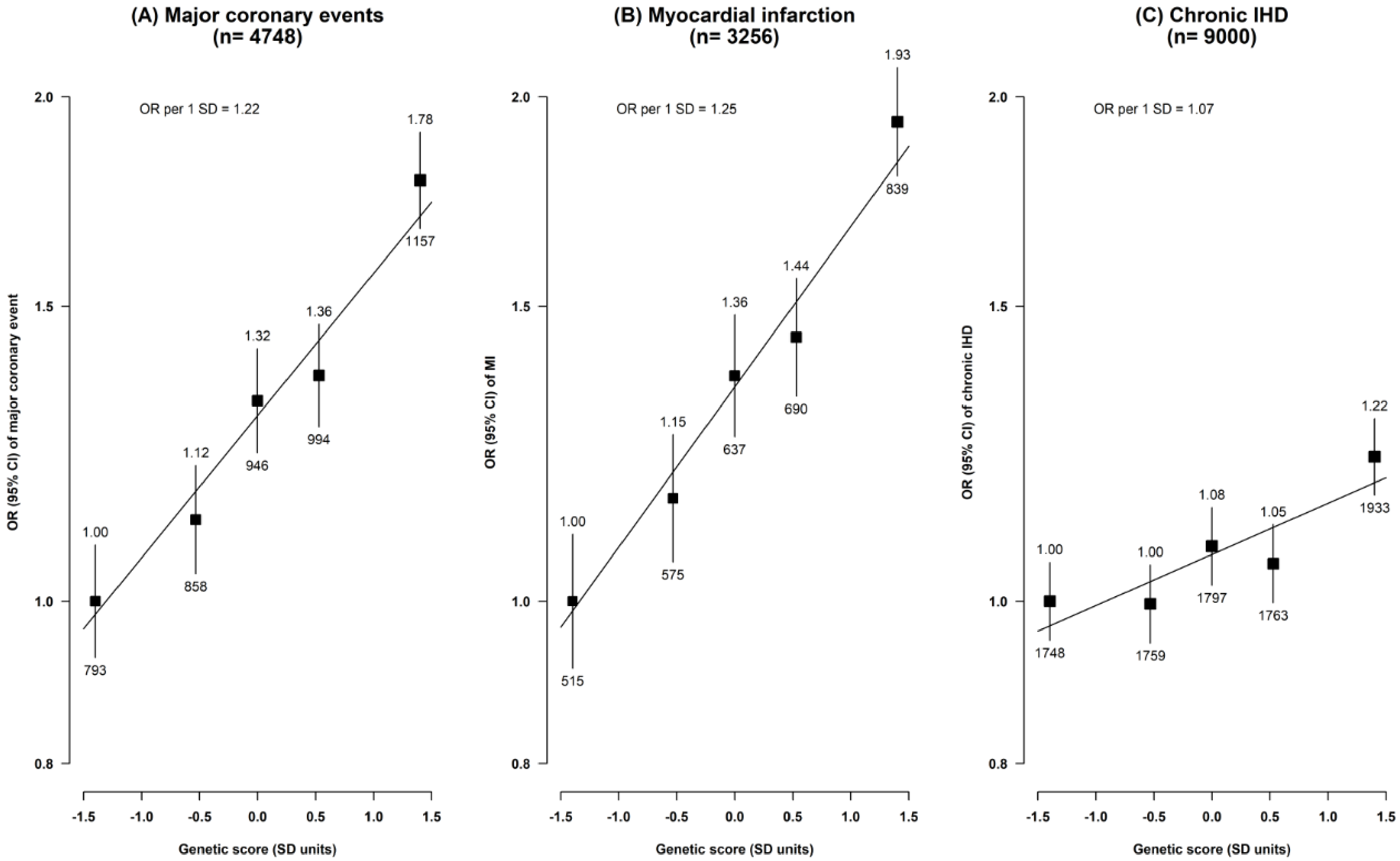

Figure 4: Odds ratios (OR) and (95% CI) of CAD, stroke and other vascular diseases in Chinese for a 1 SD higher genetic score for 224 CAD SNPs discovered in Europeans. The slope of the line represents the OR for 1 SD higher GS-CAD genetic score.

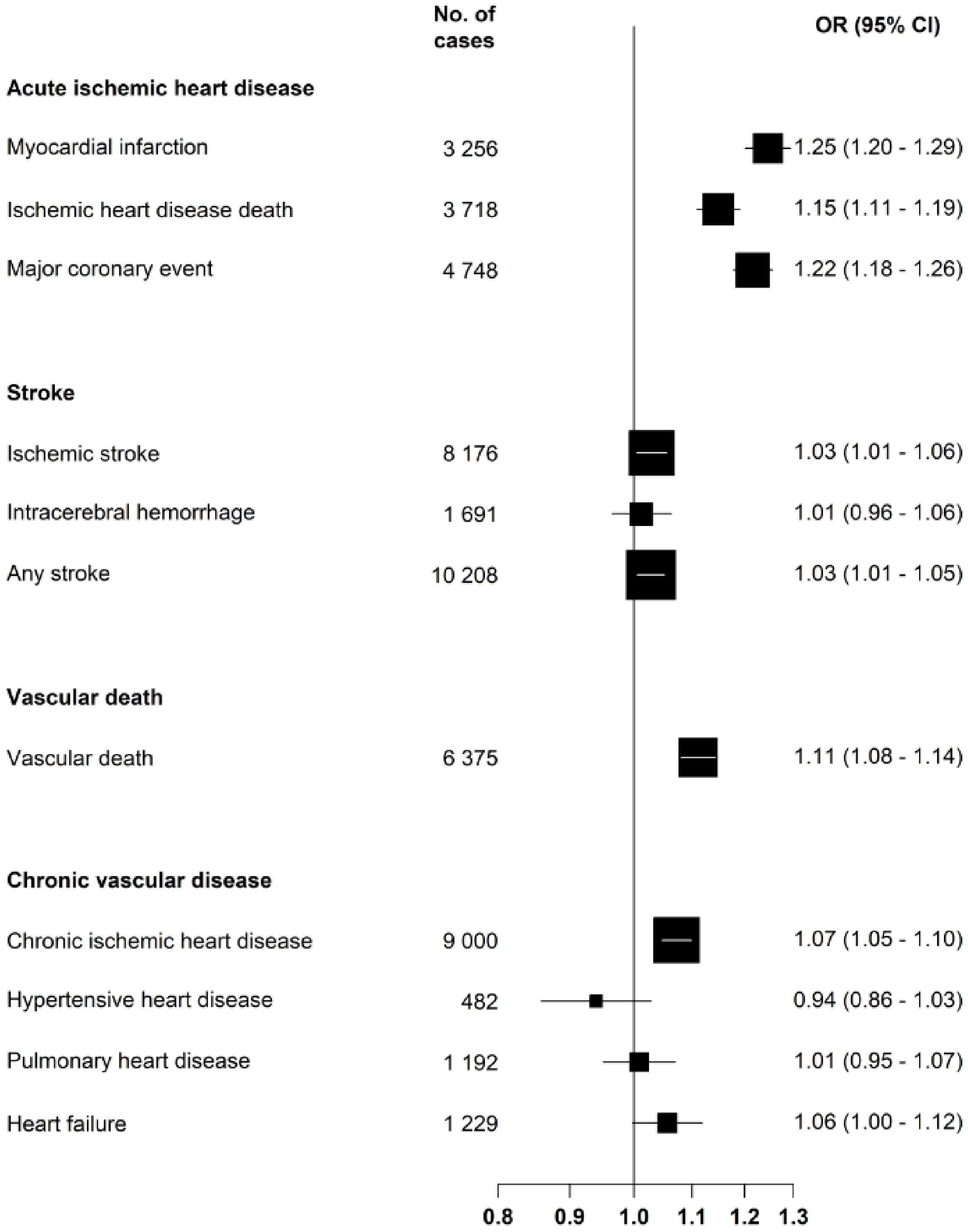

Figure 5: Associations of fifths of a European genetic score for CAD with blood pressure, body mass index and LDL-cholesterol in Chinese

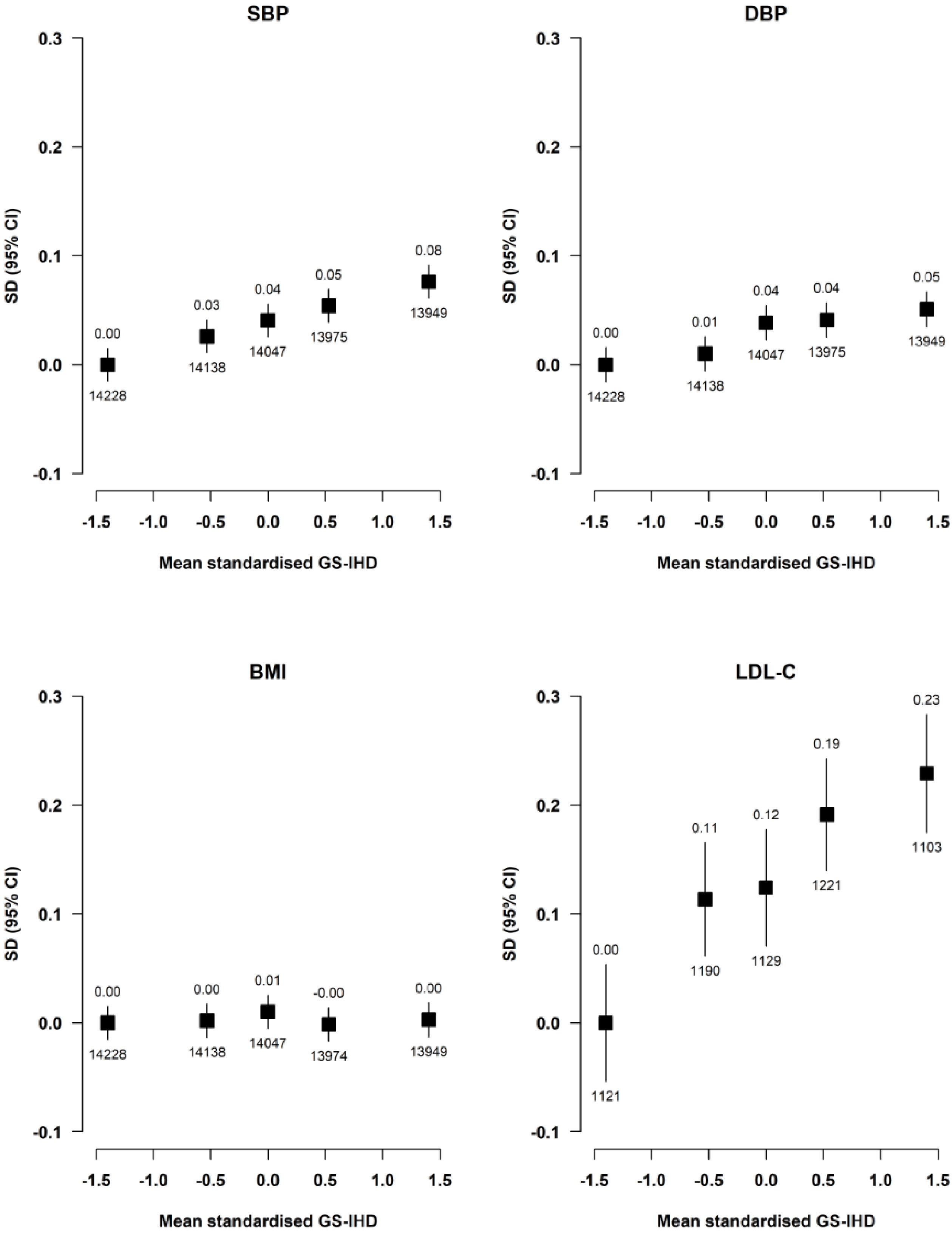

Click here to access/download
Supplementary Material
Supplementary materials for CC4D paper-9-10-2025.docx

Click here to access/download
Supplementary Material
Project_Analysis_code.R

Click here to access/download
Supplementary Material
Project_Analysis_code.R-V2-18-11-2025.txt

Click here to access/download
Supplementary Material
README.md

Personal Cover
Click here to access/download;Personal Cover;Cover letter Dr Xu -29-10-2025.docx

OXFORD POPULATION HEALTH
UNIVERSITY OF OXFORD

29 October 2025

Prof Xun Xu
Editor-in-Chief
GigaScience

Re: Concordance of genetic determinants of acute coronary artery disease between European and Chinese populations

Dear Prof Xu,

We should be very grateful if you could consider the attached report for consideration for publication in *GigaScience.* The China Kadoorie Biobank (CKB) is not as well known or as widely used as UK Biobank, but CKB is an open access study to all users for non-genetic data on 0.5M with 2 million hospital episodes and shared access to GWAS data on 100,000. BGI will soon complete WGS on 500,000 and will host a Data Shield to allow a wider access to the genetic data and disease outcomes in CKB. We should be very grateful if you might consider the attached report for publication in CKB as it is exemplar of what can be achieved in the overall cohort with WGS data on 500,000.

There has been limited GWAS studies of CAD in East Asians and hence comparative studies of associations of SNPs with CAD discovered in Europeans for acute CAD in 100,000 Chinese adults should be informative. The report compares the strength of associations of GWAS hits for CAD with CAD in East Asians. Myocardial infarction and acute CAD account for a smaller proportion of the Chinese population with ischaemic heart disease (IHD) than in Europeans and the relevance of these for acute CAD, chronic IHD and heart failure or stroke in Chinese is uncertain. In clinical practice, we are interested in targeting treatments for prevention of MACE/ASCVD outcomes, so it is interesting to assess the effects of genetic scores for acute CAD/MI, for non-MI IHD and non-MI other cardiac conditions in addition to stroke and vascular mortality.

All co-authors have approved this version of the report for publication. We believe that this report should be of interest to your readers and hope that you might consider it for publication in GigaScience. We hope that East Asian and European readers will look to your journal for high quality studies from East Asia and elsewhere.

Yours sincerely

Robert Clarke

Robert Clarke
Emeritus Professor of Epidemiology and Public Health Medicine

Richard Doll Building, Old Road Campus, Roosevelt Drive, Oxford OX3 7LF
+44(0)1865 743743 enquiries@ndph.ox.ac.uk
Head of Department
Professor Sir Rory Collins FMed Sci FRCP

Graphical abstract

Click here to access/download;Graphical Abstract;Graphical abstract.docx

Concordance of genetic determinants of acute coronary artery disease between European and Chinese populations

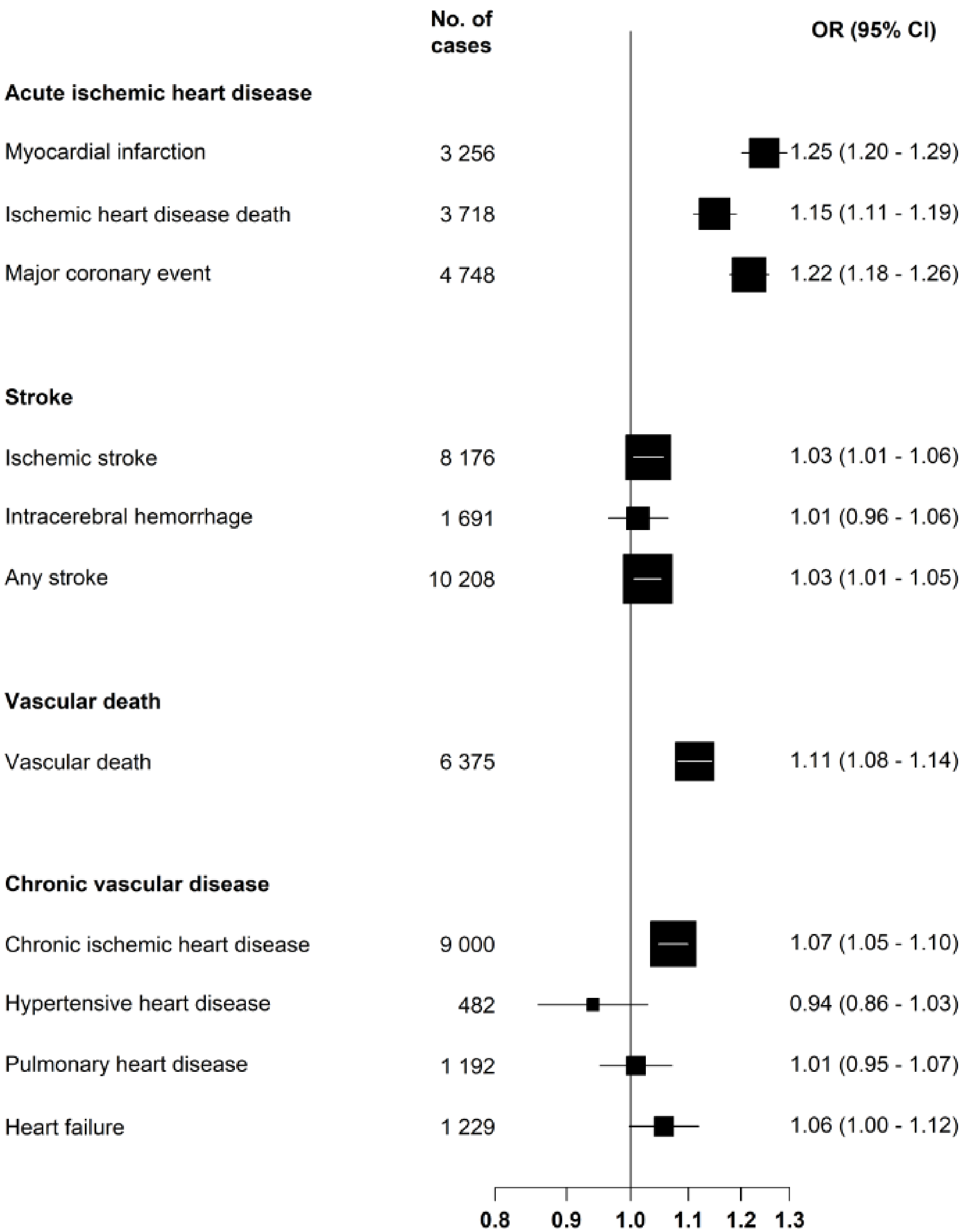

Supplement: Supplementary file 2 [file giag084_giga-d-25-00454_original_submission.pdf]
