# Supplementary material for: Concordance of genetic determinants of acute coronary artery disease between European and Chinese populations
Source: Gigascience. 2026 Aug 11;15:giag084. doi: 10.1093/gigascience/giag084 (PMC13543311; doi:10.1093/gigascience/giag084)

GigaScience

Concordance of genetic determinants of acute coronary artery disease between European and Chinese populations

--Manuscript Draft--

**Manuscript Number:**	GIGA-D-25-00454R1
**Full Title:**	Concordance of genetic determinants of acute coronary artery disease between European and Chinese populations
**Article Type:**	Research
**Funding Information:**	Wellcome Trust (212946/Z/18/Z, 202922/Z/16/Z, 104085/Z/14/Z, 088158/Z/09/Z) — Prof Zhengming Chen<br>Innovative Research Group Project of the National Natural Science Foundation of China (82192901, 82192904, 82192900, 82388102) — Prof Liming Li<br>Noncommunicable Chronic Diseases-National Science and Technology Major Project (2023ZD0510101, 2023ZD0510100) — Prof Liming Li<br>Medical Research Council (MC_UU_00017/1, MC_UU_12026/2, MC_U137686851) — Prof Zhengming Chen<br>British Heart Foundation's Big Beat Challenge award to CureHeart (BBC/F/21/220106) — Prof Hugh Watkins

Abstract:

Aims: This study examined the concordance of associations of single nucleotide polymorphisms (SNPs) for acute coronary artery disease (CAD) discovered in Europeans with acute CAD, chronic ischaemic heart disease (IHD) or stroke in Chinese adults.
Methods: In a nested case-control study of acute CAD (4748 cases/66,227 controls) in China Kadoorie Biobank (CKB), we compared associations of 224 SNPs for acute CAD discovered in Europeans in CARDIOGRAMplusC4D (CC4D) with acute CAD in Chinese. We compared associations of a genetic score (GS-CAD) for 224 SNPs for acute CAD with chronic IHD (n=9000), heart failure (n=1229), stroke (n=10,208) and CVD risk factors (n=70,364) and plasma proteomics (n=3904) in CKB.
Results: The strength of associations of 224 CAD SNPs discovered in Europeans was moderately correlated with those for acute CAD in Chinese (r=0.53), but significant associations were only replicated (p<0.05) for 25 SNPs. The strength of associations with acute CAD for a 1 SD higher genetic score for acute CAD in Chinese were 3-fold greater than for chronic IHD or heart failure (1.22 [1.18-1.26] vs 1.07 [1.05-1.10] vs 1.06 [1.00-1.12], respectively) and 7-fold greater than for stroke (1.03; 1.01-1.05).
Higher levels of GS-CAD were strongly associated with LDL-cholesterol (LDL-C), moderately with systolic blood pressure (SBP) and weakly with body mass index (BMI), but not with proteomics.
Conclusions: The findings demonstrate a moderate concordance of genetic determinants for acute CAD between Europeans and Chinese that were specific for CAD and only weakly associated with chronic IHD, heart failure or ischaemic stroke.

**Corresponding Author:**	Robert Clarke<br>Oxford University: University of Oxford<br>Oxford, Oxfordshire UNITED KINGDOM
**Corresponding Author Secondary Information:**	
**Corresponding Author's Institution:**	Oxford University: University of Oxford
**Corresponding Author's Secondary Institution:**	

Powered by Editorial Manager® and ProduXion Manager® from Aries Systems Corporation

**First Author:**	Robert Clarke, FRCP, MD
**First Author Secondary Information:**	
**Order of Authors:**	Robert Clarke, FRCP, MD
	Ahmed Edris, PhD
	Kuang Lin, PhD
	Sofia Massa, PhD
	Iona Y Millwood, DPhil
	Paul Sherliker, MSc
	Liming Li, MPH
	Jun Lv, PhD
	Canqing Yu, PhD
	Dianjianyi Sun, PhD
	Pei Pei, BSc
	Daniel Avery, MSc
	Fang Liu, BSc
	Anuj Goel, MSc
	Hugh Watkins, MD
	Zhengming Chen, DPhil
	Robin G Walters, PhD
	Derrick A Bennett, PhD
**Order of Authors Secondary Information:**	
**Response to Reviewers:**	RESPONSE TO REVIEWER COMMENTS ON GIGA-D-25-00454<br>"Concordance of genetic determinants of acute coronary artery disease between European and Chinese populations".<br><br>The verbatim comments of the two reviewers are outlined using a standard font and our responses to their comments and suggestions are made using a bold font. In addition, we itemized the page numbers where we made the revisions to the manuscript.<br><br>Reviewer #1:<br><br>1.1 Such studies are often in large size and may draw attention from the editors and readers. However, the authors may need to explain the clinical relevance of such studies either in the Introduction or in the Discussion, especially after so many high-cost GWAS studies of various technological platforms had been published in the past about three decades. Did these studies ever help in any sense or manner for health ?<br>RESPONSE: Genetic studies of complex-trait diseases have greatly advanced our knowledge of the genetic architecture of ischaemic heart disease, elucidation of causal relevance of risk factors and discovery of novel targets for treatment. Advances include three main fields: (A) Understanding the biological origins of cardiovascular disease (CVD) (B) discovery of known and novel targets for drug treatment of CVD; (C) improving risk prediction through polygenic risk scores. We have added a paragraph to the Introduction on pages 5/6 to state the following: "Genome-wide association (GWAS) studies of CVD conducted in diverse ancestry populations have greatly expanded our knowledge of risk factors for CVD, by clarifying the genetic architecture of CVD across populations, elucidation of instrumental variables for Mendelian randomization (MR) analyses to assess the causal relevance of risk factors, discovery of novel targets for drug treatment of CVD and development of polygenic scores to improve CVD risk prediction.(9-16) While meta-analyses of international consortia of GWAS studies have helped to elucidate cross-ancestry differences in CVD traits and

subtypes, large biobanks like UK Biobank, Japan Biobank and China Kadoorie Biobank can also provide additional information about differences between phenotypes, gene-gene and gene-environmental interactions that are not possible to discover in multi-ancestry meta-analyses of GWAS consortia of CVD sub-types." (17-19)

1.2. Another major issue is the study design. In this study, the cases are from a large population-based study. There may be many strengths of this study design. There are apparently also limitations. A possible major limitation of such a study design is the diagnostic accuracy of the diseases under study. Thus, the authors may need to compare this kind of studies with studies based on clinical cases collected from hospitalized patients.
RESPONSE: Prospective cohort studies offer several major advantages beyond case-control studies, especially when the goal is to understand how exposures relate to outcomes over time. Cohort studies also provide the ability to establish temporal sequence, measure incidence, and study multiple outcomes from a single exposure. We added a paragraph in the Introduction on page 5/6: "Genome-wide association (GWAS) studies of CVD conducted in diverse ancestry populations have greatly expanded our knowledge of risk factors for CVD, by clarifying the genetic architecture of CVD across populations, elucidation of instrumental variables for Mendelian randomization (MR) analyses to assess the causal relevance of such risk factors, discovery of novel targets for drug treatment of CVD and development of polygenic scores to improve risk prediction of CVD.(9-16) While meta-analyses of international consortia of GWAS studies have reported cross-ancestry differences in genetic architecture of CVD sub-types, large biobanks such as UK-Biobank, Japan Biobank or China Kadoorie Biobank can provide additional information about differences between CVD sub-types, gene-gene or gene-environmental interactions that are not possible to discover in multi-ancestry meta-analyses of GWAS consortia of summary statistics on CVD sub-types. (17-19)"
The diagnostic accuracy of first incident MI cases in Chinese adults was very high. We revised the sentence on the diagnostic accuracy of reported first incident cases of MI and all IHD cases in CKB on page 10/11: "The diagnostic accuracy study of IHD subtypes included retrieval of medical records from 19,135 reported first-incident IHD cases in CKB for clinician adjudication and reported that 10.2% (n=1948) of IHD cases had MI, with a diagnostic accuracy of 98% for reported MI and 93% for all IHD, respectively."(17)

1.3. GWAS data are only available in about 20% of the total Kadoorie study participants. The authors may need to clarify why. If the selection was for purpose, the authors should also clarify.
RESPONSE: We have revised the description of the study design and selection for GWAS, and the population subset was constrained by available financial resources for genotyping. On page 9, we added the following sentence: "The design of the GWAS subset of the CKB study was constrained by available financial resources for genotyping and limited to 105,408 samples (33,408 selected for nested case-control studies of CVD and COPD and 72,000 participants from a random sample of the CKB study population)."
Since the reviewer was concerned about why our analyses were limited to 105,000 with GWAS data, we also added sentence at the end of the report to indicate that ongoing whole genome sequence data will shortly be available on 0.5M participants in CKB that will allow us to compare associations with CAD for lower frequency variants in addition to gene-gene interactions and gene-environmental interactions that may also differ between Chinese and European ancestry populations. On page 24, we added the following statements: "When the currently planned cohort-wide whole genome sequence data are available on all 0.5M participants in CKB, it should be possible to conduct further analyses of the concordance of CAD genes for rarer variants between Europeans and Chinese in addition to CAD associations with additional individual genes, gene-gene interactions or gene-environmental interactions between Chinese and European populations".

1.4. There are several corresponding authors from the UK and several coauthors from China. This reviewer guesses that these Chinese authors might also contribute to this project significantly. Is it worthwhile to have a Chinese expert to be among the corresponding authors for the proper interpretation of the findings.

Powered by Editorial Manager® and ProduXion Manager® from Aries Systems Corporation

RESPONSE: As China Kadoorie Biobank is jointly supervised by scientists from Peking University, Beijing, China and University of Oxford, Oxford, England, we have an agreed co-authorship policy for all publications that recognize the contribution of both teams to all research outputs. All projects are reviewed by a Research Management Committee prior to any analyses and recorded on a Research Tracker system. All primary CKB reports that involve CKB data as a primary output include key co-authors from China and Oxford who are members of the steering committee of the study and these include 4 senior PI from China in addition to one PI from one of the 10 regional centers. All authors are recorded on a research management electronic platform that can be audited. We have agreed that reports led by colleagues in China include first and senior authors and corresponding authors from China and vice versa for those reports who are led by colleagues in Oxford. This division is pragmatic and equitable and works best for smooth running of publications led by the CKB Collaborative Study Group.

Reviewer #2:

2.1. Only 25 of 224 European CAD SNPs were nominally replicated in the Chinese dataset, which limits the strength of the conclusion regarding cross-ancestry concordance. The authors should discuss more fully the potential roles of limited statistical power, allele-frequency differences, and differences in linkage disequilibrium structure.
RESPONSE: We have acknowledged these limitations in the Discussion. We estimate that the study had only about 88% power to detect MCE associations with variants with a MAF of 0.2 and an OR of 1.1 at our Bonferroni significance level (0.05/224). We have modified a paragraph on the study limitations to explain the limited statistical power on page 21 as follows: "First, the number of cases of MI, MCE, or IHD were too limited to have sufficient statistical power to replicate associations with lower frequency variants or variants with smaller effect sizes. We estimate that that the present study had about 88% power to detect MCE associations with variants with a MAF of 0.2 and an OR of 1.1 at our Bonferroni significance level (0.05/224), with lower power to detect associations with variants with lower MAF (e.g. 16% for MAF of 0.05 and OR of 1.1).(47)
2.2. The genetic score was constructed using European-derived weights; this is reasonable for testing transferability, but the authors should clarify whether alternative score constructions were evaluated and whether recalibration in the Chinese population changes the results.
RESPONSE: The rationale of the paper was to use conventional methods to estimate the polygenic risk score using European weights, because they were based on much larger sample sizes. In due course, the CKB will obtain genetic data on all 500,000 in the study and then have much greater power to generate improved polygenic scores using Chinese weights. We have acknowledged this limitation in the Discussion and added a sentence at the end of the Discussion. On Page 24, we state: "When the currently planned cohort-wide whole genome sequence data are available on all 0.5M participants in CKB, it should be possible to conduct further analyses of the concordance of CAD genes for rarer variants between Europeans and Chinese in addition to CAD associations with additional individual genes, gene-gene interactions or gene-environmental interactions between Chinese and European populations.

2.3. The interpretation that the shared genetic architecture is partly mediated through LDL-C and SBP appears stronger than warranted by the presented analyses, which are associative rather than formal mediation analyses. This wording should be softened unless additional analyses are provided. The proteomic analysis is interesting but appears underpowered relative to the large number of proteins tested, so the negative findings should be interpreted more cautiously.
RESPONSE: We have revised the statements on mediation of genetic risk scores for ischemic heart disease by LDL-C and SBP and more importantly by proteomic analyses. We have acknowledged that the moderate sample size of IHD cases precludes formal mediation analyses and added this to the limitations section in the Discussion. On page 19 the modified the statement on LDL-C and SBP states: "With the exception of LDL-C and SBP, the GS-CAD was not strongly associated with other CAD risk factors,(38, 39) but the study included too few CAD cases with GWAS data and plasma lipids at baseline to conduct formal mediation analyses of CAD by levels of

Powered by Editorial Manager® and ProduXion Manager® from Aries Systems Corporation

	LDL-C and SBP.”
**Additional Information:**	
**Question**	**Response**
Are you submitting this manuscript to a special series or article collection?	No
**Experimental design and statistics**<br><br>Full details of the experimental design and statistical methods used should be given in the Methods section, as detailed in our Minimum Standards Reporting Checklist. Information essential to interpreting the data presented should be made available in the figure legends.<br><br>Have you included all the information requested in your manuscript?	Yes
**Resources**<br><br>A description of all resources used, including antibodies, cell lines, animals and software tools, with enough information to allow them to be uniquely identified, should be included in the Methods section. Authors are strongly encouraged to cite Research Resource Identifiers (RRIDs) for antibodies, model organisms and tools, where possible.<br><br>Have you included the information requested as detailed in our Minimum Standards Reporting Checklist?	Yes
**Availability of data and materials**<br><br>All datasets and code on which the conclusions of the paper rely must be either included in your submission or deposited in publicly available repositories (where available and ethically appropriate), referencing such data using a unique identifier in the references and in the “Availability of Data and Materials” section of your manuscript.	No

Powered by Editorial Manager® and ProduXion Manager® from Aries Systems Corporation

Have you have met the above requirement as detailed in our Minimum Standards Reporting Checklist?	
If not, please give reasons for any omissions below.<br><br>as follow-up to "**Availability of data and materials**<br><br>All datasets and code on which the conclusions of the paper rely must be either included in your submission or deposited in publicly available repositories (where available and ethically appropriate), referencing such data using a unique identifier in the references and in the "Availability of Data and Materials" section of your manuscript.<br><br>Have you have met the above requirement as detailed in our Minimum Standards Reporting Checklist?<br><br>"	We can provide summary statistics for all genetic data on publication of the report.
GigaScience has policies and guidelines in place for the use of generative AI-writing tools such as ChatGPT. If you have used such writing tools to assist with writing the manuscript this must be declared and cited in the text. Authors should not list AI-writing tools and other AI-assisted technologies as an author or co-author and should acknowledge that they are fully responsible for text generated or refined by AI-writing tools.<p><br><br>A summary of use (particularly in the introduction or among methods) needs to be included at the end of the paper, and the outputs should also be included as a supplementary file hosted in GigaDB or other open repositories. Please read our guidelines for more information. <p><br><br>By submitting to GigaScience, you are aware of the journal's AI-writing tools	No

policy, and if you have declared use of such tools below, you have acknowledged this where appropriate in your manuscript and have made a summary of use and outputs available. <p><br><b>AI-assisted writing tools have been used in the preparation of this manuscript?	

Manuscript

Click here to access/download;Manuscript;CC4D paper-3-8-2026-V9-CLEAN final.docx

Concordance of genetic determinants of acute coronary artery disease between European and Chinese populations

Ahmed Edris PhD[1], Kuang Lin PhD[1], Sofia Massa PhD[1], Iain Turnbull MB,[1] Iona Y. Millwood DPhil[1], Paul Sherliker MSc[1], Liming Li MPH[2,3,4], Jun Lv PhD[2,3,4], Canqing Yu PhD[2,3,4], Dianjianyi Sun PhD[2,3,4], Pei Pei BSc[3], Daniel Avery MSc[1], Fang Liu BSc[5], Anuj Goel MSc[6], Hugh Watkins MD[6], Zhengming Chen DPhil[1], Robin G. Walters PhD[1*], Derrick A. Bennett PhD[1*], Robert Clarke MD[1*] on behalf of the China Kadoorie Biobank Study Group[†]

[1] Clinical Trial Service Unit, Nuffield Department of Population Health, University of Oxford, Oxford, UK;

[2] Department of Epidemiology & Biostatistics, School of Public Health, Peking University, Beijing, China;

[3] Peking University Center for Public Health and Epidemic Preparedness and Response, Beijing, China;

[4] Key Laboratory of Epidemiology of Major Diseases, Peking University, Ministry of Education, Beijing, China;

[5] Suzhou CDC, Suzhou, Jiangsu, China;

[6] Division of Cardiovascular Medicine, Radcliffe Department of Medicine, University of Oxford, Oxford, UK.

*Equal contribution; [†] Members of the CKB Study Group are shown in the appendix.

Address for correspondence:

Professor Robert Clarke or Professor Derrick Bennett

Nuffield Department of Population Health
Old Road Campus
Roosevelt Drive
Oxford
OX3 7LF
United Kingdom

Email: robert.clarke@ndph.ox.ac.uk derrick.bennett@ndph.ox.ac.uk

3 August 2026

Short title: Genetic determinants of acute coronary artery disease

Word count: Abstract 246; Text: 4752

Key words: Genetic determinants, acute coronary artery disease, stroke, other vascular diseases, Chinese and European populations.

Abstract

Aims: This study examined the concordance of associations of single nucleotide polymorphisms (SNPs) for acute coronary artery disease (CAD) discovered in Europeans with acute CAD, chronic ischaemic heart disease (IHD) or stroke in Chinese adults.

Methods: In a nested case-control study of acute CAD (4748 cases/66,227 controls) in China Kadoorie Biobank (CKB), we compared associations of 224 SNPs for acute CAD discovered in Europeans in CARDIOGRAMplusC4D (CC4D) with acute CAD in Chinese. We compared associations of a genetic score (GS-CAD) for 224 SNPs for acute CAD with chronic IHD (n=9000), heart failure (n=1229), stroke (n=10,208) and CVD risk factors (n=70,364) and plasma proteomics (n=3904) in CKB.

Results: The strength of associations of 224 CAD SNPs discovered in Europeans was moderately correlated with those for acute CAD in Chinese (r=0.53), but significant associations were only replicated (p<0.05) for 25 SNPs. The strength of associations with acute CAD for a 1 SD higher genetic score for acute CAD in Chinese were 3-fold greater than for chronic IHD or heart failure (1.22 [1.18-1.26] vs 1.07 [1.05-1.10] vs 1.06 [1.00-1.12], respectively) and 7-fold greater than for stroke (1.03; 1.01-1.05). Higher levels of GS-CAD were strongly associated with LDL-cholesterol (LDL-C), moderately with systolic blood pressure (SBP) and weakly with body mass index (BMI), but not with proteomics.

Conclusions: The findings demonstrate a moderate concordance of genetic determinants for acute CAD between Europeans and Chinese that were specific for CAD and only weakly associated with chronic IHD, heart failure or ischaemic stroke.

Introduction

Coronary artery disease (CAD) and stroke are the leading causes of morbidity and mortality worldwide.[1] The worldwide prevalence of these diseases has increased almost 2-fold over the last three decades,[2] and CVD mortality currently accounts for about 40% of all deaths in China.[3, 4] Premature deaths (<70 years) from cardiovascular disease (CVD) have declined in both Western and Chinese populations over the last few decades reflecting improvements in lifestyle and access to medical treatment to reduce levels of CVD risk factors.

The prevalence of current cigarette smoking is 4-fold higher in Chinese men than in European men (44% vs 12%), but mean levels of low-density lipoprotein cholesterol (LDL-C) (2.5 vs 3.0 mmol/L) or body ma the causal ress index are lower (24.5 vs 27.5 kg/m^2) in Chinese than in European men and women.[5-8] However, little is known about the comparative importance of genetic determinants for acute CAD (i.e., non-fatal myocardial infarction or death from ischaemic heart disease [IHD]) discovered in Europeans for acute CAD and other CVD subtypes in Chinese adults.

Genome-wide association (GWAS) studies of CVD conducted in diverse ancestry populations have greatly expanded our knowledge of risk factors for CVD, by clarifying the genetic architecture of CVD across populations, elucidation of instrumental variables for Mendelian randomization (MR) analyses to assesslevance of such risk factors, discovery of novel targets for drug treatment of CVD and development of polygenic scores to improve risk prediction of CVD.[9-16] While meta-analyses of international consortia of GWAS studies have reported cross-ancestry differences in genetic architecture of CVD sub-types, large biobanks such as UK-Biobank, Japan Biobank or China Kadoorie Biobank can provide additional information about differences between CVD sub-types, gene-gene or gene-environmental interactions

that are not possible to discover in multi-ancestry meta-analyses of GWAS consortia of summary statistics on CVD sub-types. [17-19]

Genome-wide association studies of CAD in European ancestry populations have reported over 241 single nucleotide polymorphisms (SNPs) that were independently associated with CAD demonstrating a polygenic genetic architecture involving multiple SNPs in Europeans each typically having only modest effects on acute CAD.[9-11] While the effects of some of these SNPs on CAD are mediated through levels of LDL-C, SBP, diabetes or adiposity, the mechanisms for effects of other SNPs on acute CAD are not fully understood.[11-16] Moreover, the relevance of the genetic variants for acute CAD (MI and IHD death) for other non-MI IHD subtypes (i.e., chronic IHD), non-IHD cardiac outcomes or stroke has not been widely studied in Chinese adults. [10]

The different IHD subtypes vary by severity of coronary artery occlusion and reflect underlying pathology and severity of CAD. Acute coronary artery disease (CAD) (involving myocardial infarction [MI] or death from ischaemic heart disease [IHD]), also referred to as "major coronary events" is the most serious form of acute CAD that reflects complete occlusion of one or more coronary arteries with plaque rupture and superimposed thrombosis. In contrast, the more common chronic IHD cases typically have partial occlusion of one or more coronary arteries. Acute MI is distinguished from unstable angina or chronic IHD by elevated plasma levels of troponins and characteristic ST segment changes and or pathological Q waves on serial electrocardiogram (ECG) recordings.

Comparative studies assessing the concordance of genetic determinants for acute CAD discovered in Europe for acute CAD, chronic IHD and non-IHD cardiac diseases occurring in Chinese adults with different diet and lifestyle habits should be informative

about the relevance of genetic susceptibility for acute CAD, chronic IHD, stroke and other non-IHD cardiac outcomes in China.

A recent genome-wide association study (GWAS) of CAD, involving about 180,000 CAD cases identified independent associations of 241 variants at 198 loci with CAD in Europeans.[9] Comparison of GWAS results for CAD discovered in Europeans with 30,000 CAD cases in Japanese also demonstrated consistent effect sizes for many variants, and a combined meta-analysis of CAD from European and Japanese cases yielded 38 additional variants for CAD. Previous GWAS of CAD conducted in Chinese adults identified some shared loci for CAD between Europeans and Chinese.[10, 11] Differences in the genetic architecture or frequency of common variants or effect modification by levels of established CAD risk factors could account for some differences in the incidence and severity of CAD or proportions with different IHD subtypes between Chinese and European populations.[11-13] However, there have been few comparative studies of GWAS variants for acute CAD, chronic IHD and other non-IHD cardiac outcomes in Chinese and European populations and their relevance for other CVD subtypes.[14-16]

The aims of this study were to: (i) compare the associations of individual SNPs for acute CAD discovered in European ancestry populations in CARDIOGRAMplusC4D with acute CAD cases in Chinese; (ii) assess the concordance of associations of acute CAD SNPs discovered in Europeans for acute CAD, chronic IHD, non-IHD cardiac conditions and stroke types using a genetic score combining such European ancestry SNPs for CAD (GS-CAD) in a nested case-control studies in Chinese individuals in CKB; (iii) and compare associations of fifths of GS-CAD scores with levels of LDL-C, systolic blood pressure (SBP), body mass index (BMI) and plasma proteomics in controls with GWAS data in CKB. [9, 17,18]

Methods

Study population

Details of the design and methods used in the CKB study have been previously reported.[18, 19] Briefly, the CKB study involves a 12-year follow-up of population-based prospective study of 512,891 adults aged 30-79 years that were recruited from 10 regions in China between 2004 and 2008. All participants completed an interviewer-administered questionnaire that collected data on demographic and lifestyle characteristics (including smoking and alcohol consumption), medical history, and current use of medications. GWAS data are currently available on about one fifth of the CKB study population (constrained by available resources) involving a population subset and additional cases with MCE, stroke and other CVD subtypes and with CAD risk factors (**Figure 1**). The present report involved five separate comparisons with different subsets of the CKB study population, including: (A) associations of individual SNPs for major coronary events (MCE) that were discovered in Europeans with MCE in Chinese and a comparative study of a genetic score combining these SNPs (GS-CAD) with MCE or non-MI (chronic IHD) in CKB; (B) comparison of a GS-CAD score with stroke, heart failure or vascular death in CKB; (C) comparison of a GS-CAD score with levels of major CVD risk factors; (E) phenome-wide association study of a GS-CAD score with incidence of major diseases in CKB (**Figure 1**).

All participants underwent a physical examination that recorded measurements of anthropometry, blood pressure (BP), and spirometry, and provided a blood sample. Ethics approval was obtained from both University of Oxford (Oxford Tropical Research Ethics Committee), the Chinese Academy of Medical Sciences, Peking University and the China National Centers for Disease Control. In addition, approvals were obtained from institutional research boards at the 10 local regions. All

participants provided written informed consent for participation in this study and for their health status to be monitored by linkage with their electronic health records.

Established CVD risk factors included SBP that was measured as the mean of the last two measurements that were recorded after 5 minutes in the seated position.[20] Plasma lipid levels were measured in a subset of the population (n=18,728) using AU680 chemistry analysers (Beckman-Coulter), which used immunoassays for LDL-C and HDL-C, and immunoturbidimetric assays for total cholesterol and triglycerides.[21] Plasma levels of 2923 proteins were measured using the Olink Explore 3072 panel (OLINK Biosciences, Uppsala, Sweden) for a subset of 1971 CAD cases and 2001 sub-cohort controls.[22, 23]

Genome wide genotyping

DNA extraction, genotyping, array design, and population structure analyses for the CKB have been previously described.[24] Two custom-designed arrays were used to genotype about 800,000 variants in a total of 105,408 CKB DNA samples (**Figure 1)**. The design of the GWAS subset of the CKB study was constrained by available financial resources for genotyping and limited to 105,408 samples (33,408 selected for nested case-control studies of CVD and COPD and 72,000 participants from a random sample of the CKB study population). On the basis of disease follow-up to January 1, 2014, this initial genotyping included all incident cases of intracerebral haemorrhage (ICH; n= 5,020), subarachnoid haemorrhage (SAH; n=455), and fatal IHD (n=753); randomly selected incident cases of ischaemic stroke (IS; n=5,662), MI (n=1,008), and COPD (n=5,376); participants with no CVD events (n = 10,038) at the

time of this study. The updated array design was then used to genotype a further 72,000 population samples (thereafter referred to as "population-subset"), including all available additional cases of ICH (n=602), SAH (n=46), MI (n=1,028), and fatal IHD (n=163) that had not previously been genotyped. After exclusion of samples on the basis of call rate, excess hetero- or homozygosity, sex mismatch, XY aneuploidy, and non-East-Asian ancestry, genome-wide genotype data were available on 100,706 participants. Using ~532,000 variants that passed quality control for both array versions, imputation into the TOPMed and WBBC reference panels[22] yielded ~50 million variants with an info score >0.3. Genetic analyses used genotypes extracted as dosages of the minor alleles according to the imputed probability of each genotype. Details of national and regional principal components derived for CKB have been previously published.[25]

CAD, stroke and other vascular diseases

Disease outcomes were obtained by electronic linkage via a unique national identity number to death and disease registries (CAD, stroke, cancer and diabetes) and to the national health insurance system which records all in-patient hospital events. Data on all incident disease outcomes were integrated and standardised using the International Classification of Diseases 10th Edition (ICD-10) coded incident disease outcomes. Incident cases of CAD were defined as follows (**Table S1**): (i) acute MI (ICD-10 codes: I21-I23); (ii) major coronary events (MCE), defined as either acute MI (I21-I23) or death from IHD (underlying cause of death, I20-I25); and (iii) IHD mortality (defined as any mortality event with ICD-10 I20-I25). In addition, incident cases of eight other cardiovascular disease (CVD) outcomes were defined as follows: ischaemic stroke (IS: I63), intra-cerebral haemorrhage (ICH: I61), all stroke, (I60-61 and I63-64), vascular death (fatal I00-I99 and R96); chronic IHD (I25); hypertensive heart disease

(I11), pulmonary heart disease (I26-I28) and heart failure (I50). The diagnostic accuracy study of IHD subtypes included retrieval of medical records from 19,135 reported first-incident IHD cases in CKB for clinician adjudication and reported that 10.2% (n=1948) of IHD cases had MI, with a diagnostic accuracy of 98% for reported MI and 93% for all IHD, respectively.[17]

Logistic regression was used to assess associations of CAD SNPs with CAD or other IHD types, cases with MCE (n=4748) and chronic IHD (n=9000) were compared with controls within the population subset (n=62,107) (**Figure 1A**). Likewise, analyses of GS-CAD with stroke, CVD death and heart failure, stroke cases (n=10208), vascular deaths (n=6375) and HF cases (n=1229) were compared with 62,107 controls in the population subset (**Figure 1B**). Linear regression was used to assess associations of GS-CAD with blood pressure (n=70,364), BMI (n=70,363) and LDL-C (n=5764), respectively. PheWAS analyses of GS-CAD for common incident major diseases in 62,107 control participants from the population subset (**Figure 1C**) and logistic regression was used to assess associations with all common diseases, involving 200 or more first incident cases with GWAS data (**Figure 1D**). Linear regression was used to assess associations of GS-CAD with 2923 proteins in 1962 CAD cases and 1942 controls (**Figure 1E**).

Statistical analyses

Replication of associations of European CAD variants with CVD in Chinese

Logistic regression models were used to assess associations with each CVD subtype in each region, after adjusting for age at baseline, age squared, ever smoking (defined as: current or former regular smoking), sex, and the appropriate number of regional principal components per region. The region-specific results were then combined

using an inverse-variance weighted (IVW) meta-analysis. Group-specific variances and 95%CI were used for categorical variables to enable comparisons between any two categories (including the reference category).[26, 27] After aligning effect alleles, Pearson correlation coefficients were used to compare the coefficients for CAD outcomes and minor allele frequencies across ancestries. For assessment of replication, analyses were limited to SNPs with an allele frequency >0.01 and an info score>0.8 in the CKB. SNPs with a minor allele frequency <0.01 in East Asians and indels were excluded.

Construction of a genetic score for CAD

Figure 1 shows a flow diagram of the study design and number of participants in genetic analyses of CAD SNPs with major coronary events and chronic IHD, other CVD, CVD risk factors and phenome-wide association analysis (PheWAS) with all common major disease outcomes in CKB. **Figure S1** shows the number of individual SNPs and genetic score combining these SNPs in CKB. Genetic scores (GS) for CAD were estimated using the dosages of the 224 European ancestry genome-wide significant SNPs in CC4D that were available in CKB). The weighted GS-CAD score was computed using weights based on the log ORs for CAD in European ancestry participants in CC4D using the "riskScore" function from the PredictABEL R package,[28] and standardized using a Z-score (GS- GS mean)/(GS standard deviation). Participants were assigned to fifths of the GS-CAD score to assess the shape of the association with CVD outcomes.

Associations of European GS-CAD with CAD, stroke and other CVD outcomes in Chinese

Analyses of associations with CVD risk factors were limited to the population sub-cohort for blood pressure (n=70,364) and BMI (n=70,363) (**Figure 1**). The associations with LDL-C were performed in a subset of 5764 controls with no history of CAD or stroke. The associations of fifths of a GS-CAD score with CAD and other CVD outcomes were assessed separately by region using logistic regression after adjusting for age at baseline, age squared, ever smoking, sex, and regional principal components. Likewise, the associations of fifths of GS-CAD with levels of CAD risk were assessed using region-specific linear regression models, after adjusting for age at baseline, age squared, ever smoking, sex, and regional principal components.

Phenome-wide association analyses

A phenome-wide association analysis (PheWAS) was conducted using 117 disease phecodes, where each had 200 or more cases in the population subset of genotyped individuals in CKB. Phecodes were curated groups of ICD codes for incident cases of common major diseases with available GWAS data.[29, 30] Phecodes mapping and definitions were obtained from the Center for Precision Medicine, Vanderbilt University Medical Center, Nashville, USA.[29, 30] Logistic regression analyses were conducted for each phecode, and adjusted for age at baseline, age squared, sex, region, smoking and the first 11 national principal components. A Bonferonni significance level was estimated after taking account of the number of phenotypes.

Proteome-wide analyses

A proteome-wide analysis was conducted in 3977 participants with available data on 2923 proteins in CKB that were assayed using the Olink Explore panel to assess associations of GS-CAD with plasma proteomics (**Figure 1**). Linear regression analyses were used for each protein, adjusted for age at baseline, age squared, sex, region, smoking, plate ID and the first national 11 principal components. A 5% false discovery rate (FDR) correction was used, and a Bonferonni-threshold was also estimated based on the number of protein associations tested.

Cis-protein quantitative trait loci (*cis*-pQTLs), defined as the cis-variants associated with plasma levels of 2923 proteins at GWAS significance level were used as instrumental variables for MR analyses.[31] Region-specific logistic regression models were used to investigate the associations of *cis*-pQTLs with MCE, after adjustment for age, age squared, sex, ever smoking, and the appropriate number of regional PCs. The results were subsequently combined using inverse-variance weighted meta-analysis using the Wald ratio.[31] Directionality of the associations was assessed using Steiger filtering.[32] In addition, one sample MR was conducted as a sensitivity analysis, limiting the sample to the proteomics subset, using the OneSampleMR package.[33]

Results

The baseline characteristics of CKB participants with GWAS data are shown separately for cases with major coronary events (MCE) or chronic IHD and controls in **Table 1**. Overall, compared with controls, cases with MCE were older, and more likely to be male, but similar proportions of cases and controls lived in urban and rural areas. The proportions of ever-smokers in Chinese were much greater in male cases and male controls than in female cases or female controls. MCE cases had a higher prevalence of diabetes or hypertension than controls. Likewise, MCE cases had higher mean levels of SBP and DBP than controls, respectively. Conversely, mean levels of BMI did not differ between MCE cases and controls.

Cross-ancestry comparisons

Details of the SNPs selected for analysis in the CKB are provided in **Figure S1**. A total of 224 SNPs that were significantly associated with MCE in CC4D were available in CKB. Overall, the effect estimates for the 224 CC4D SNPs were positively correlated with CAD in both ancestries (correlation coefficient=0.53, Chinese in CKB and Europeans in CC4D: **Figure 2A**). A total of 25 European SNPs were significantly associated with MCE ($p<0.05$) and the associations for these SNPs with acute CAD in Chinese and Europeans were directionally consistent in both populations (**Figure 2B, Table S2**). Among these SNPs for CAD in CKB, 11 were previously associated with blood pressure, 7 with lipids, 3 with diabetes and 3 with BMI.[9]

Associations of GS-CAD with CAD, stroke and other CVD subtypes

Analyses of associations of fifths of GS-CAD genetic score with MCE, MI or chronic IHD indicated linear positive associations with different IHD types, but the strength of the associations (OR, 95%CI) with MCE were 3-fold greater than for chronic IHD

(**Figure 3**: OR and 95%CI for MCE:1.22 [95%CI:1.18-1.26]; MI:1.25 [1.20-1.29]; and chronic IHD:1.07 [1.05-1.10] per 1 SD higher GS-CAD, respectively). The effects of 1 SD higher GS-CAD for 11 different CVD outcomes were examined in CKB (**Figure 4**). A 1 SD higher level of GS-CAD score was associated with a higher risk of MCE, MI, fatal IHD, vascular death and chronic IHD. However, a 1 SD higher level of GS-CAD was only weakly associated with a higher risk of any stroke (chiefly IS, but not with ICH) or with heart failure, but not at all with hypertensive heart disease or pulmonary heart disease. The top 3 variants most strongly associated with higher risks of major coronary events (MCE) in Chinese included *APOE* and two variants for *CDKN2B-AS1* and the top 3 variants inversely associated with MCE included *PRDM16, LPA*, and *ABHD2,* respectively, consistent with the findings in Europeans. Overall, the OR (95%CI) for MCE for a genetic score for all 224 SNPs was similar to the OR for a subset of 25 variants that were successfully replicated ($p<0.05$) with MCE in CKB (1.22; [1.18-1.26] vs 1.21 [1.17-1.25]: **Table S3**).

Associations of GS-CAD with CVD risk factors

Figure 5 shows associations of fifths of a genetic risk score for acute CAD (GS-CAD) with mean levels of CVD risk factors including: (A) SBP; (B) DBP, (C) BMI and (D) LDL-C, respectively. The findings indicated strong linear positive associations of increasing fifths of GS-CAD with higher levels LDL-C, but only moderately strong associations with higher levels of SBP and weaker associations with DBP, but no associations with BMI (**Figure 5**).

PheWAS of GS-CAD score with other CVD and non-CVD disease outcomes

A total of 15 major diseases, involving 117 phenotypes were tested in phenome-wide analyses for associations with a 1 SD higher GS-CAD genetic score (**Figure S2**).

Three major diseases were significantly associated with the GS-CAD after correction for multiple testing: MI (OR: 1.26; 95%CI: 1.20-1.33), coronary atherosclerosis (OR: 1.10; 95%CI: 1.07-1.13) and occlusion of cerebral arteries (OR: 1.04; 95%CI: 1.02-1.08), respectively, highlighting the specificity for these associations.

Associations of GS-CAD score with plasma proteomics

Analyses of associations of GS-CAD with plasma levels of 2923 proteins indicated that a 1 SD higher score was associated with higher levels of two proteins after correction for multiple testing: Lipoprotein-associated phospholipase A2 (PLA2G7: coefficient 0.05; 95%CI: 0.03-0.07) and Sushi-containing domain protein (SUSD2: coefficient: -0.02; 95%CI: -0.01- -0.04) (**Figure S3**). However, there was association of GS-CAD with SUSD2 in a sensitivity analysis after excluding variants within 1 mB of the causal gene region for each protein. Furthermore, two cis-PQTLs were identified, one for each of the two proteins, (PLA2G7 and SUSD2: **Table S4**), but both one-sample and two-sample MR analyses provided no causal support for CAD for either of these two proteins (**Figure S4**).

Discussion

This study examined the associations of 224 SNPs for CAD that were discovered in European studies with CAD in Chinese adults and confirmed a moderate concordance of genetic determinants of CAD between these two diverse ancestry populations. Despite a relatively limited number of SNPs (n=25) being nominally significantly associated with CAD (at p<0.05) in CKB, a weighted GS-CAD score using weights derived from the European population was strongly associated with CAD in Chinese,

but only weakly associated with chronic IHD or CVD mortality in Chinese adults. Individuals in the top vs bottom fifths of a genetic score (GS-CAD) for CAD were associated with a 2-fold higher risk of MI, but with only 20% higher risk of chronic IHD. The GS-CAD score was only weakly associated with a higher risk of IS, and was unrelated with HS.

The GS-CAD score was also strongly associated with mean levels of LDL-C and SBP, and to lesser extent with DBP, but not with BMI. A PheWAS analysis for 117 phenotypes in Chinese adults indicated that the GS-CAD score was significantly associated with higher risk of MI, coronary atherosclerosis and occlusion of the cerebral arteries, respectively. Analysis of associations of GS-CAD with 2923 proteins demonstrated associations with PLA2G7 and SUSD2, but MR analyses did not provide any causal support for associations of either of these two proteins with CAD.

The number of CVD deaths and proportions of total deaths due to CVD have increased exponentially in China compared with Western countries in recent decades.[34] Several studies have also reported a greater disease severity for CAD in East Asians than in Europeans.[35] Heritability of CAD has been estimated to be ~40-60% in twin studies [15] (with the independent variants for IHD identified in the present study explaining ~36% of heritability), it is likely that most of the differences in incidence of acute CAD or CAD severity between European and East Asians may reflect differences in diet and lifestyle or variable access to medical treatment.[9] Comparing the associations of genetic variants for CAD in different genetic ancestries can help to understand the genetic architecture for CAD in diverse populations. Moreover, as most of GWAS studies have been conducted in European populations, the generalizability of the resulting GWAS or GS-CAD genetic scores for CAD in other ancestries will require further validation in future cycles of international GWAS consortia of CAD that will

include a greater number of CAD cases from East Asian populations.[36] A previous study of associations of European ancestry GS-CAD genetic score with CAD in non-European participants in UK-Biobank reported a good correlation across most included ancestries, except for Africans, but the analyses were based on small numbers of CAD cases from non-European ancestry populations.[37] The percentage of replicated SNPs with CAD and correlation between effect sizes for CAD between Europeans and Chinese were consistent with those previously reported between European and Japanese ancestry populations.[9]

Moreover, a European ancestry GS-CAD score was associated with a higher risk of coronary artery atherosclerosis and cerebral artery occlusion in phenome-wide analyses. The results are consistent with a shared aetiology of atherosclerotic diseases between Chinese and European ancestries. With the exception of LDL-C and SBP, the GS-CAD was not strongly associated with other CAD risk factors,[38, 39] but the study included too few CAD cases with GWAS data and plasma lipids at baseline to conduct formal mediation analyses of CAD by levels of LDL-C and SBP.

In a separate comparative report of GWAS associations of Lp(a)-related variants with Lp(a) levels in Chinese vs Europeans in the CKB study, we demonstrated only a modest overlap of Lp(a)-related variants with Lp(a) levels in both populations with only a single SNP rs73596816 being strongly associated with Lp(a) levels in both Chinese and Europeans.[40] In contrast, the two lead SNPs most strongly associated with Lp(a) levels and MI in Europeans[41] were unrelated to Lp(a) or MI in Chinese with rs10455872 being very rare and rs3798120 SNP having only a minimal effect on Lp(a) levels in Chinese. However, our previous study reported that the rs73596816 SNP was strongly related to Lp(a) levels and risk of MI in both Chinese and East Asians populations. The present report confirmed the comparable strength of associations of

rs73596816 with risk of MI in both ancestry populations (OR [95%CI]: 1.13 [1.02-1.25] vs 1.11 [1.08-1.14] in Chinese vs Europeans, respectively).[40] Despite differences in the genetic architecture of the *LPA* locus between Chinese and Europeans, both the present and previous reports demonstrated that equivalent genetically-predicted differences in Lp(a) levels had comparable effects on MI in both ancestry populations.[40,41]

The present analysis demonstrated only a weak positive association of the GS-CAD score with ischaemic stroke or occlusion of cerebral arteries in a PheWAS analysis in addition to MI and coronary atherosclerosis. Hence, the results of this study add to a growing body of evidence highlighting the shared biological pathways between acute CAD and large artery ischaemic stroke and provide further support for lowering LDL-C and SBP for prevention of occlusive atherosclerotic vascular diseases in both populations.

In contrast, we found little evidence of shared genetic pathways between CAD with hypertensive or pulmonary heart diseases. Previous studies suggested some shared heritability between CAD and ischaemic stroke,[42] and a significant overlap of genetic variants between ischaemic stroke and CAD.[43] Importantly, six known CAD risk loci, including 9p21 (the locus with the smallest p-value in our replication analysis), were previously associated with either ischaemic stroke.[44] Despite the 9p21 locus being one of the most studied variants for IHD, the mechanism of action of this locus on risk of CAD has yet to be elucidated. Importantly, the 9p21 locus has been previously associated with atherosclerosis, but not with thrombosis or plaque rupture.[45] The findings suggesting that 9p21 may play a role in both CAD and acute IS mediated partly through large artery atherosclerosis in both Chinese and European populations.

The chief strengths of the present study include the large number of cases of CAD, stroke and other CVD outcomes and CVD risk factors from a single prospective study in Chinese adults recruited from 10 diverse geographical regions in China.[35, 46] Prospective cohort studies offer several major advantages beyond case-control studies, especially when the goal is to understand how exposures relate to outcomes over time. Cohort studies also enable analyses of the temporal sequence and measurement of incident disease and study of multiple outcomes from single exposures. However, the study also had several limitations. First, the number of cases of MI, MCE, or IHD were too limited to have sufficient statistical power to replicate associations with lower frequency variants or variants with smaller effect sizes. We estimate that that the present study had about 88% power to detect MCE associations with variants with a MAF of 0.2 and an OR of 1.1 at our Bonferroni significance level (0.05/224), with lower power to detect associations with variants with lower MAF (e.g. 16% for MAF of 0.05 and OR of 1.1).[47] Second, we excluded phenotypes with less than 200 participants in our PheWAS analysis, and, hence, could have missed some phenotypes associated with GS-CAD score.[30] An alternative explanation for the null associations of CAD-GS with plasma proteomics suggest that CAD SNPs may be involved in initiation of atherosclerosis of the coronary arteries, but plasma proteomics may more closely related to determinants of progression of atherosclerosis.

Conclusions

Overall, this study demonstrated a moderate concordance of genetic determinants of acute CAD between European and Chinese adults and a GS-CAD genetic score combining all such variants was moderately strongly associated with acute CAD in Chinese adults. However, the strength of association of this GS-CAD genetic score

with acute CAD was 3-fold stronger than that for chronic IHD or heart failure and 7-fold stronger than that for stroke. The GS-CAD score was also strongly associated with levels of LDL-C or SBP, but only moderately associated with BMI. The results highlight a shared genetic architecture of CAD between Europeans and Chinese that is partly mediated through levels of LDL-C and SBP and provides support for lowering mean levels of these risk factors for prevention of CAD in Chinese population. When the currently planned cohort-wide whole genome sequence data are available on all 0.5M participants in CKB, it should be possible to conduct further analyses of the concordance of CAD genes for rarer variants between Europeans and Chinese in addition to CAD associations with additional individual genes, gene-gene interactions or gene-environmental interactions between Chinese and European populations.

Abbreviations

BBJ: Biobank Japan, BMI: Body mass index, CC4D CARDIoGRAMplusC4D, CKB China Kadoorie Biobank, CDC Center of Disease Control, CVD Cardiovascular diseases, DBP Diastolic blood pressure, ECG Electrocardiogram, FDR False discovery rate, GS Genetic score, GS-CAD Genetic risk score for CAD, GWAS Genome-wide association studies, HDL-C High-density lipoprotein cholesterol, IHD Ischaemic heart disease, LDL-C Low-density lipoprotein cholesterol, Mb Megabase, MCE Major coronary events, MI Myocardial infarction, MR Mendelian randomization, PheWAS Phenome-wide association studies, PLA2G7 Lipoprotein-associated phospholipase A2, pQTLs Protein quantitative trait loci, QC Quality control, SBP Systolic blood pressure, SNPs Single nucleotide polymorphisms, SUSD2 Sushi-containing domain protein, TOPMed Trans-Omics for Precision Medicine, WBBC West Lake Biobank for Chinese, WHR Waist-to-hip ratio.

Availability of source code and requirements

•Project name: Edris-2025-genetic-determinants-of-acute-coronary-artery-disease

• Project repository: https://github.com/ckbiobank/Edris-2025-genetic-determinants-of-acute-coronary-artery-disease

• License: MIT license

• Operating system(s): Platform independent

• Programming language: R Project for Statistical Computing

• RRID:SCR_001905

Author contributions

Conceptualization: RC. Methodology: AE and SM conducted the statistical analyses, generated tables and figures; KL identified genetic instruments for the study with advice from DAB. Formal analysis: AE, SM, KL. Investigation: AE, SM, RC and DAB supervised the investigation. Resources and Data Curation: CQ supervised local data acquisition and CY, JL, DS, PP, LL, FL and ZC coordinated data acquisition for the study, DA supervised curation of data set for this report. IYM and RGW supervised genomewide analyses in CKB. Visualization: PS generated all the figures. Supervision: RC and DAB. Project Administration: ZC and LL are joint co-principal investigators for the study. Funding acquisition: ZC and LL. Writing original draft: AE, RC and DAB wrote the initial draft. Writing, reviewing and editing: All other authors provided additional comments on the report.

Funding

The funding body for the baseline survey was the Kadoorie Charitable Foundation, Hong Kong, China and the funding sources for the long-term continuation of the study include UK Wellcome Trust (212946/Z/18/Z, 202922/Z/16/Z, 104085/Z/14/Z, 088158/Z/09/Z), grants from the National Natural Science Foundation of China (82192901, 82192904, 82192900, 82388102) and the Noncommunicable Chronic Diseases-National Science and Technology Major Project (2023ZD0510101, 2023ZD0510100). Core funding was provided to the CTSU, University of Oxford, by the British Heart Foundation, the UK Medical Research Council, and Cancer Research UK. The long-term follow-up was funded in part by the UK Wellcome Trust (212946/Z/18/Z, 202922/Z/16/Z, 104085/Z/14/Z, 088158/Z/09/Z). HW was supported by the British Heart Foundation's Big Beat Challenge award to CureHeart (BBC/F/21/220106) and AG was supported by PG/24/11862.

Acknowledgements

The chief acknowledgment is to the participants, CKB project staff, staff of the China CDC and its regional offices for access to death and disease registries. The Chinese National Health Insurance scheme provided electronic linkage to all hospital admissions. The CKB study is jointly coordinated by the University of Oxford and the Chinese Academy of Medical Sciences.

Data availability

Data for this study is described on CKB Research Tracker (DAR-2023-00342: Ahmed Edris Mohamed). Data on the analyses of associations of the 224 CAD SNPs for acute CAD in Europeans[9] and results in Chinese are available under University of Oxford's Open Source International (MIT) license. The code used for data analysis, the data underlying the figures presented in this study, and the summary results on associations of the 224 CAD SNPs with acute CAD and other vascular outcomes in CKB are included in the CKB Project GitHub[48].

Data access statement

The China Kadoorie Biobank (CKB) is a blood-based prospective study of 512,000 Chinese adults. The data for the present report are accessible to investigators via the CKB study website[49]. Details about the study are provided on the CKB data access pages[50]. Data from baseline, first and second resurveys, and disease follow-up are available under the CKB Open Access Data Policy to bona fide researchers. Sharing of genotyping data is constrained by the Administrative Regulations on Human Genetic Resources of the People's Republic of China. Access to these and certain other data is available through collaboration with CKB researchers. Genotyping data were exported from China to the Oxford CKB International Coordinating Centre under Data Export Approvals 2014-13 and 2015-39 from the Office of Chinese Human Genetic Resource Administration.

Competing interests

The authors declare no competing interests.

References

1. Vaduganathan M, Mensah GA, Turco JV, Fuster V, Roth GA. The Global Burden of Cardiovascular Diseases and Risk: A Compass for Future Health. J Am Coll Cardiol. 2022;80(25):2361-71. https://doi.org/10.1016/j.jacc.2022.11.005
2. Roth GA, Mensah GA, Johnson CO, Addolorato G, Ammirati E, Baddour LM, et al. Global Burden of Cardiovascular Diseases and Risk Factors, 1990-2019: Update From the GBD 2019 Study. J Am Coll Cardiol. 2020;76(25):2982-3021. https://doi.org/10.1016/j.jacc.2020.11.010.
3. Li JJ, Liu HH, Li S. Landscape of cardiometabolic risk factors in Chinese population: a narrative review. Cardiovasc Diabetol. 2022;21(1):113. https://doi.org/10.1186/s12933-022-01551-3.
4. Rehman S, Rehman E, Mumtaz A, Jianglin Z. Cardiovascular Disease Mortality and Potential Risk Factor in China: A Multi-Dimensional Assessment by a Grey Relational Approach. Int J Public Health. 2022;67:1604599. doi: 10.3389/ijph.2022.1604599.
5. Doi T, Langsted A, Nordestgaard BG. Lipoproteins, Cholesterol, and Atherosclerotic Cardiovascular Disease in East Asians and Europeans. J Atheroscler Thromb. 2023;30(11):1525-46. https://doi.org/10.5551/jat.RV22013
6. Oakkar EE, Stevens J, Truesdale KP, Cai J. BMI and all-cause mortality among Chinese and Caucasians: the People's Republic of China and the Atherosclerosis Risk in Communities Studies. Asia Pac J Clin Nutr. 2015;24(3):472-9. doi: 10.6133/apjcn.2015.24.3.12.
7. Lewington S, Lacey B, Clarke R, Guo Y, Kong XL, Yang L, et al. The Burden of Hypertension and Associated Risk for Cardiovascular Mortality in China. JAMA Intern Med. 2016;176(4):524-32. doi:10.1001/jamainternmed.2016.0190
8. World Health Organization. WHO Global Report on Trends in Prevalence of Tobacco Use 2000-2025. 2021. ISBN: 978-92-4-011627-6
9. Aragam KG, Jiang T, Goel A, Kanoni S, Wolford BN, Atri DS, et al. Discovery and systematic characterization of risk variants and genes for coronary artery disease in over a million participants. Nat Genet. 2022;54(12):1803-15. https://doi.org/10.1038/s41588-022-01233-6
10. Lu X, Wang L, Chen S, He L, Yang X, Shi Y, et al. Genome-wide association study in Han Chinese identifies four new susceptibility loci for coronary artery disease. Nat Genet. 2012;44(8):890-4. https://doi.org/10.1038/ng.2337
11. Assimes TL, Roberts R. Genetics: Implications for Prevention and Management of Coronary Artery Disease. J Am Coll Cardiol. 2016;68(25):2797-818. https://doi.org/10.1016/j.jacc.2016.10.039
12. Dandona S, Stewart AF, Chen L, Williams K, So D, O'Brien E, et al. Gene dosage of the common variant 9p21 predicts severity of coronary artery disease. J Am Coll Cardiol. 2010;56(6):479-86. DOI: 10.14797/mdcj-10-1-7
13. Jing J, Su L, Zeng Y, Tang X, Wei J, Wang L, et al. Variants in 9p21 Predicts Severity of Coronary Artery Disease in a Chinese Han Population. Ann Hum Genet. 2016;80(5):274-81. https://doi.org/10.1111/ahg.12163
14. Ralapanawa U, Sivakanesan R. Epidemiology and the Magnitude of Coronary Artery Disease and Acute Coronary Syndrome: A Narrative Review. J Epidemiol Glob Health. 2021;11(2):169-77. https://doi.org/10.2991/jegh.k.201217.001.
15. Zdravkovic S, Wienke A, Pedersen NL, Marenberg ME, Yashin AI, De Faire U. Heritability of death from coronary heart disease: a 36-year follow-up of 20 966 Swedish twins. J Intern Med. 2002;252(3):247-54. https://doi.org/10.1046/j.1365-2796.2002.01029.x
16. Tcheandjieu C, Zhu X, Hilliard AT, Clarke SL, Napolioni V, Ma S, et al. Large-scale genome-wide association study of coronary artery disease in genetically diverse populations. Nat Med. 2022;28(8):1679-92. https://doi.org/10.1038/s41591-022-01891-3.

17. Turnbull I, Clarke R, Wright N, Sun D, Kaartsonaki C, Pei P, Yu C et al. Diagnostic accuracy, treatment and prognosis of myocardial infarction: an 11-year follow-up of a community-based cohort of 0.5 million Chinese adults. BMJ PublicHealth.2026;4(1):e004019. doi: 10.1136/bmjph-2025-004019.

18. Chen Z, Chen J, Collins R, Guo Y, Peto R, Wu F, et al. China Kadoorie Biobank of 0.5 million people: survey methods, baseline characteristics and long-term follow-up. Int J Epidemiol. 2011;40(6):1652-66. https://doi.org/10.1093/ije/dyr120.
19. Walters RG, Millwood IY, Lin K, Schmidt Valle D, McDonnell P, Hacker A, et al. Genotyping and population characteristics of the China Kadoorie Biobank. Cell Genom. 2023 Jul 20;3(8):100361. doi: 10.1016/j.xgen.2023.100361.
20. Clarke R, Wright N, Walters R, Gan W, Guo Y, Millwood IY, et al. Genetically Predicted Differences in Systolic Blood Pressure and Risk of Cardiovascular and Noncardiovascular Diseases: A Mendelian Randomization Study in Chinese Adults. Hypertension. 2023;80(3):566-76. https://doi.org/10.1161/HYPERTENSIONAHA.122.20120
21. Sun L, Clarke R, Bennett D, Guo Y, Walters RG, Hill M, et al. Causal associations of blood lipids with risk of ischaemic stroke and intracerebral haemorrhage in Chinese adults. Nat Med. 2019;25(4):569-74. https://doi.org/10.1038/s41591-019-0366-x
22. Pozarickij A, Said S, Lin K, et al. Ancestry diversity in the genetic determinants of the human plasma proteome enhances identification of potential drug targets. MedRxiv 2023.11.13.23298365; doi: https://doi.org/10.1101.
23. Mazidi M, Wright N, Yao P, Kartsonaki C, Millwood IY, Fry H, et al. Plasma Proteomics to Identify Drug Targets for Ischaemic Heart Disease. J Am Coll Cardiol. 2023;82(20): 1906-20. https://doi.org/10.1016/j.jacc.2023.09.804.
24. Walters RG, Millwood IY, Lin K, Valle DS, McDonnell P, Hacker A, et al. Genotyping and population structure of the China Kadoorie Biobank. medRxiv. 2022:2022.05.02.22274487.
25. Walters RG, Millwood IY, Lin K, Schmidt Valle D, McDonnell P, Hacker A, et al. Genotyping and population characteristics of the China Kadoorie Biobank. Cell Genom. 2023;3(8):100361. https://doi.org/10.1016/j.xgen.2023.100361
26. Easton DF, Peto J, Babiker AG. Floating absolute risk: an alternative to relative risk in survival and case-control analysis avoiding an arbitrary reference group. Stat Med. 1991;10(7):1025-35. https://doi.org/10.1002/sim.4780100703.
27. Iona A, Bragg F, Guo Y, Yang L, Chen Y, Pei P, et al. Adiposity and risks of vascular and non-vascular mortality among Chinese adults with type 2 diabetes: a 10-year prospective study. BMJ Open Diabetes Res Care. 2022;10(1). https://doi.org/10.1136/bmjdrc-2021-002489
28. Kundu S, Aulchenko YS, van Duijn CM, Janssens AC. PredictABEL: an R package for the assessment of risk prediction models. Eur J Epidemiol. 2011;26(4):261-4. https://doi.org/10.1007/s10654-011-9567-4
29. Verma A, Bradford Y, Dudek S, Lucas AM, Verma SS, Pendergrass SA, et al. A simulation study investigating power estimates in phenome-wide association studies. BMC Bioinformatics. 2018;19(1):120. https://doi.org/10.1186/s12859-018-2135-0.
30. Bastarache L. Using Phecodes for Research with the Electronic Health Record: From PheWAS to PheRS. Annu Rev Biomed Data Sci. 2021;4:1-19. https://doi.org/10.1146/annurev-biodatasci-122320-112352
31. Hemani G, Zheng J, Elsworth B, Wade KH, Haberland V, Baird D, et al. The MR-Base platform supports systematic causal inference across the human phenome. Elife. 2018;7. https://doi.org/10.7554/eLife.34408
32. Hemani G, Tilling K, Davey Smith G. Orienting the causal relationship between imprecisely measured traits using GWAS summary data. PLoS Genet. 2017;13(11):e1007081. https://doi.org/10.1371/journal.pgen.1007081

33. Palmer T, Spiller, W. and Sanderson, E. OneSampleMR - R package for one-sample Mendelian randomization and instrumental variable analyses. 2021. https://remlapmot.github.io/OneSampleMR/
34. World Health Organization. Regional Office for the Western P. People's Republic of China health system review. Manila: WHO Regional Office for the Western Pacific; 2015 2015. ISBN: 9789290617280
35. Gijsberts CM, Seneviratna A, de Carvalho LP, den Ruijter HM, Vidanapthirana P, Sorokin V, et al. Ethnicity Modifies Associations between Cardiovascular Risk Factors and Disease Severity in Parallel Dutch and Singapore Coronary Cohorts. PLoS One. 2015;10(7):e0132278. doi: 10.1371/journal.pone.0132278.
36. O'Sullivan JW, Raghavan S, Marquez-Luna C, Luzum JA, Damrauer SM, Ashley EA, et al. Polygenic Risk Scores for Cardiovascular Disease: A Scientific https://doi.org/10.1161/CIR.0000000000001077Statement From the American Heart Association. Circulation 2022;146(8):e93-e118. https://doi.org/10.1161/CIR.0000000000001077
37. Mars N, Kerminen S, Feng YA, Kanai M, Lall K, Thomas LF, et al. Genome-wide risk prediction of common diseases across ancestries in one million people. Cell Genom. 2022;2(4). doi: 10.1016/j.xgen.2022.100118.
38. Kuchenbaecker K, Telkar N, Reiker T, Walters RG, Lin K, Eriksson A, et al. The transferability of lipid loci across African, Asian and European cohorts. Nat Commun. 2019;10(1):4330. https://doi.org/10.1038/s41467-019-12026-7.
39. Kilpelainen TO, Bentley AR, Noordam R, Sung YJ, Schwander K, Winkler TW, et al. Multi-ancestry study of blood lipid levels identifies four loci interacting with physical activity. Nat Commun. 2019;10(1):376. 10.1038/s41467-018-08008-w
40. Clarke R, Wright N, Lin K, Yu C, Walters RG, Lv J, Hill M, Kartsonaki C, Millwood IY, Bennett DA, Avery D, Yang L, Chen Y, Du H, Sherliker P, Yang X, Sun D, Li L, Qu C, Marcovina S, Collins R, Chen Z, Parish S; China Kadoorie Biobank Collaborative Group. Causal Relevance of Lp(a) for Coronary Heart Disease and Stroke Types in East Asian and European Ancestry Populations: A Mendelian Randomization Study. Circulation. 2025; 151: 1699-1711. https://doi.org/10.1161/CIRCULATIONAHA.124.07208
41. Clarke R, Peden JF, Hopewell JC, et al. Genetic variants associated with Lp(a) lipoprotein level and coronary disease. N Engl J Med 2009; 361: 2518-2528. DOI: 10.1056/NEJMoa0902604
42. Banerjee A, Lim CC, Silver LE, Welch SJ, Banning AP, Rothwell PM. Familial history of stroke is associated with acute coronary syndromes in women. Circ Cardiovasc Genet. 2011;4(1):9-15. https://doi.org/10.1161/CIRCGENETICS.110.957688.
43. Dichgans M, Malik R, Konig IR, Rosand J, Clarke R, Gretarsdottir S, et al. Shared genetic susceptibility to ischaemic stroke and coronary artery disease: a genome-wide analysis of common variants. Strohttps://doi.org/10.1007/s11883-015-0498-5ke. 2014;45(1):24-36. https://doi.org/10.1161/STROKEAHA.113.002707
44. Kessler T, Erdmann J, Dich.gans M, Schunkert H. Shared genetic aetiology of coronary artery disease and atherosclerotic stroke - 2015. Curr Atheroscler Rep. 2015;17(4):498. https://doi.org/10.1007/s11883-015-0498-5.
45. Chan K, Patel RS, Newcombe P, Nelson CP, Qasim A, Epstein SE, et al. Association between the chromosome 9p21 locus and angiographic coronary artery disease burden: a collaborative meta-analysis. J Am Coll Cardiol. 2013;61(9):957-70. http://dx.doi.org/10.1016/j.jacc.2012.10.051
46. Hou L, Chen S, Yu H, Lu X, Chen J, Wang L, et al. Associations of PLA2G7 gene polymorphisms with plasma lipoprotein-associated phospholipase A2 activity and coronary artery disease in a Chinese Han population: the Beijing atherosclerosis study. Hum Genet. 2009;125(1):11-20. https://doi.org/10.1093/ije/dyw087.

47. Skol AD, Scott LJ, Abecasis GR, Boehnke M. Joint analysis is more efficient than replication-based analysis for two-stage genome-wide association studies. Nat Genet. 2006;38:209-13. https://doi.org/10.1038/ng1706.
48. Edris A, Lin K, Massa S, Turnbull I, Millwood IY, Sherliker P, et al. China Kadoorie Biobank Study Group. Edris-2025-genetic-determinants-of-acute-coronary-artery-disease. GitHub. https://github.com/ckbiobank/Edris-2025-genetic-determinants-of-acute-coronary-artery-disease.
49. China Kadoorie Biobank. www.ckbiobank.org. Accessed 15 Jul 2026.
50. China Kadoorie Biobank Data Access. www.ckbiobank.org/site/ Data+Access. Accessed 15 Jul 2026.

Figure legends

Figure 1: Flow diagram of study design and numbers of participants in genetic analyses of CAD SNPs with: (A) Major Coronary Events and chronic IHD, (B) other CVD, (C) CVD risk factors, (D) PheWAS with 117 diseases and (E) plasma proteomics in Chinese adults.

Figure 2: Odds ratio (OR) and 95% CI of (A) major coronary events in Chinese for 224 individual CAD SNPs discovered in Europe; and (B) Odds ratio (OR) and 95% CI of major coronary events in Chinese for a subset of 25 SNPs that were replicated in Chinese.

Figure 3: Odds ratios (OR) and (95%CI) of (A) major coronary events, (B) myocardial infarction or (C) chronic IHD in Chinese for fifths of a genetic score (GS-CAD) for 224 CAD SNPs discovered in Europeans.

Figure 4: Odds ratios (OR) and (95%CI) of CAD, stroke, and other vascular diseases in Chinese for a 1 SD higher genetic score for 224 CAD SNPs discovered in Europeans.

Figure 5: Associations of fifths of a genetic score for 224 CAD SNPs discovered in Europeans with blood pressure, body mass index and LDL-cholesterol in Chinese. Values shown are line plots for 1 SD higher levels of systolic blood pressure (SBP), diastolic blood pressure (DBP), body mass index (BMI) and LDL-cholesterol (LDL-C) versus mean standardised genetic score (GS-CAD) per quintile. The number of participants included in each quintile is indicated below each data point.

Table 1: Baseline characteristics of participants with acute and chronic coronary artery disease and controls

Mean (SD) or %	Major Coronary Events (n=4748)	Chronic Ischaemic Heart disease (n=9000)	Controls (n=62107)
**Demographic factors**			
Age, mean (SD), yr	62.2 (9.6)	58.9 (9.7)	51.1 (10.4)
Female, %	43.1	61.8	59.3
Urban, %	42.0	56.5	42.8
**Lifestyle factors**			
Male ever-smokers, %	78.0	75.3	74.4
Female ever-smokers, %	9.5	7.4	3.0
Male ever-drinkers, %	41.8	46.8	49.7
Female ever-drinkers, %	4.1	6.0	4.2
**Medical history**			
Diabetes*, %	16.6	11.1	5.0
Hypertension*, %	26.4	24.0	8.9
**Clinical measurements**,			
SBP, mean (SD), mmHg	146 (25)	138(24)	131 (21)
DBP, mean (SD), mmHg	81 (13)	80 (12)	78 (11)
BMI, mean (SD), Kg/m^2	23.8 (3.9)	24.6 (3.9)	23.6 (3.4)

*Self-reported doctor-diagnosed diabetes or diabetes identified by blood glucose at baseline; MCE: Major coronary events, chronic IHD: chronic ischaemic heart disease; SBP: systolic blood pressure; DBP: diastolic blood pressure; BMI: body mass index

Figure 1: Flow diagram of study design and numbers of participants in genetic analyses of CAD SNPs with: (A) Major Coronary Events and chronic IHD, (B) other CVD, (C) CVD risk factors, (D) PheWAS with 117 diseases and (E) plasma proteomics in Chinese adults.

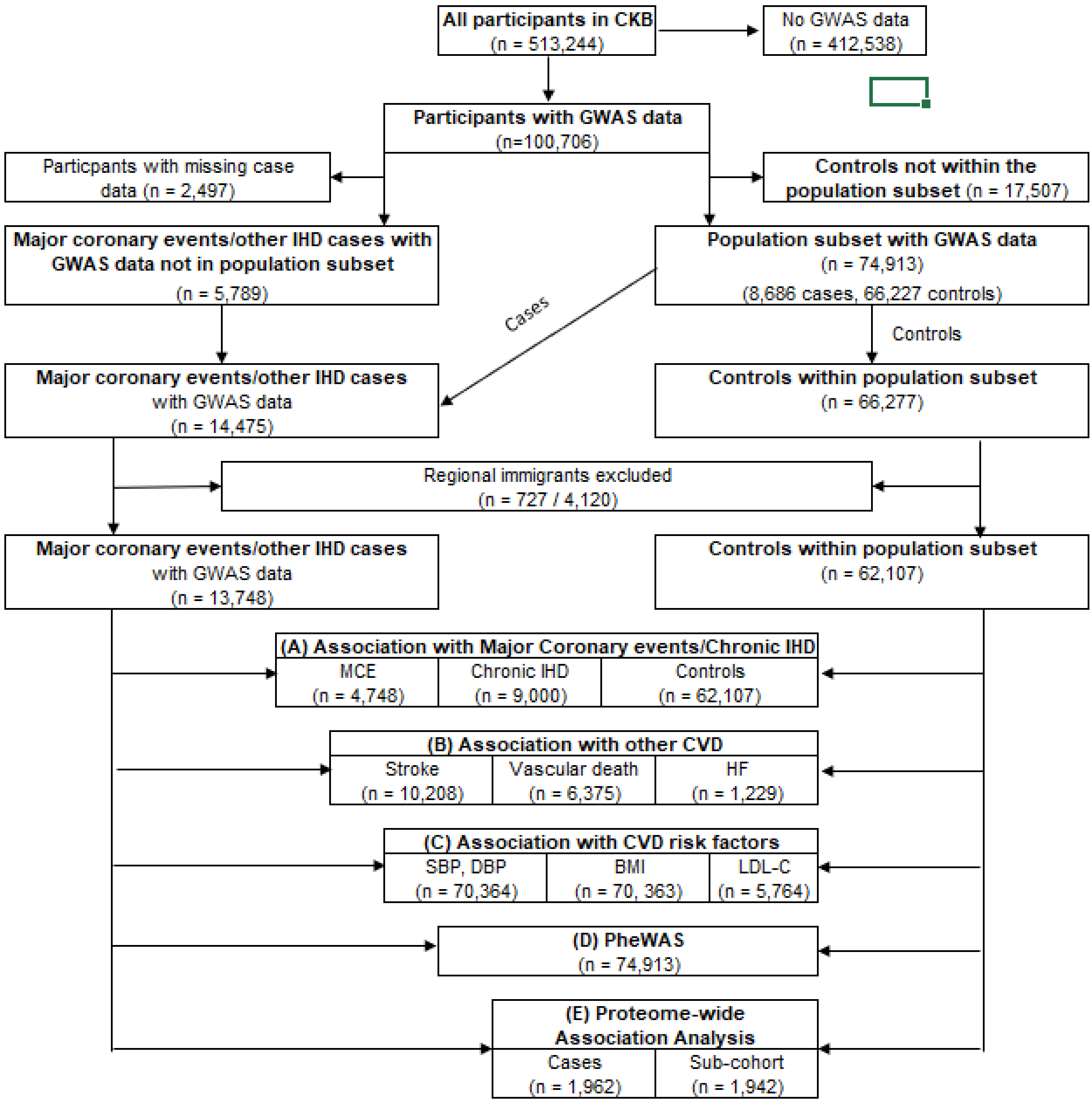

Figure 2: Associations of 224 European CAD SNPs with (A) CAD in Chinese and Europeans (B) and associations with CAD for a subset of 25 SNPs that were successfully replicated in CKB.

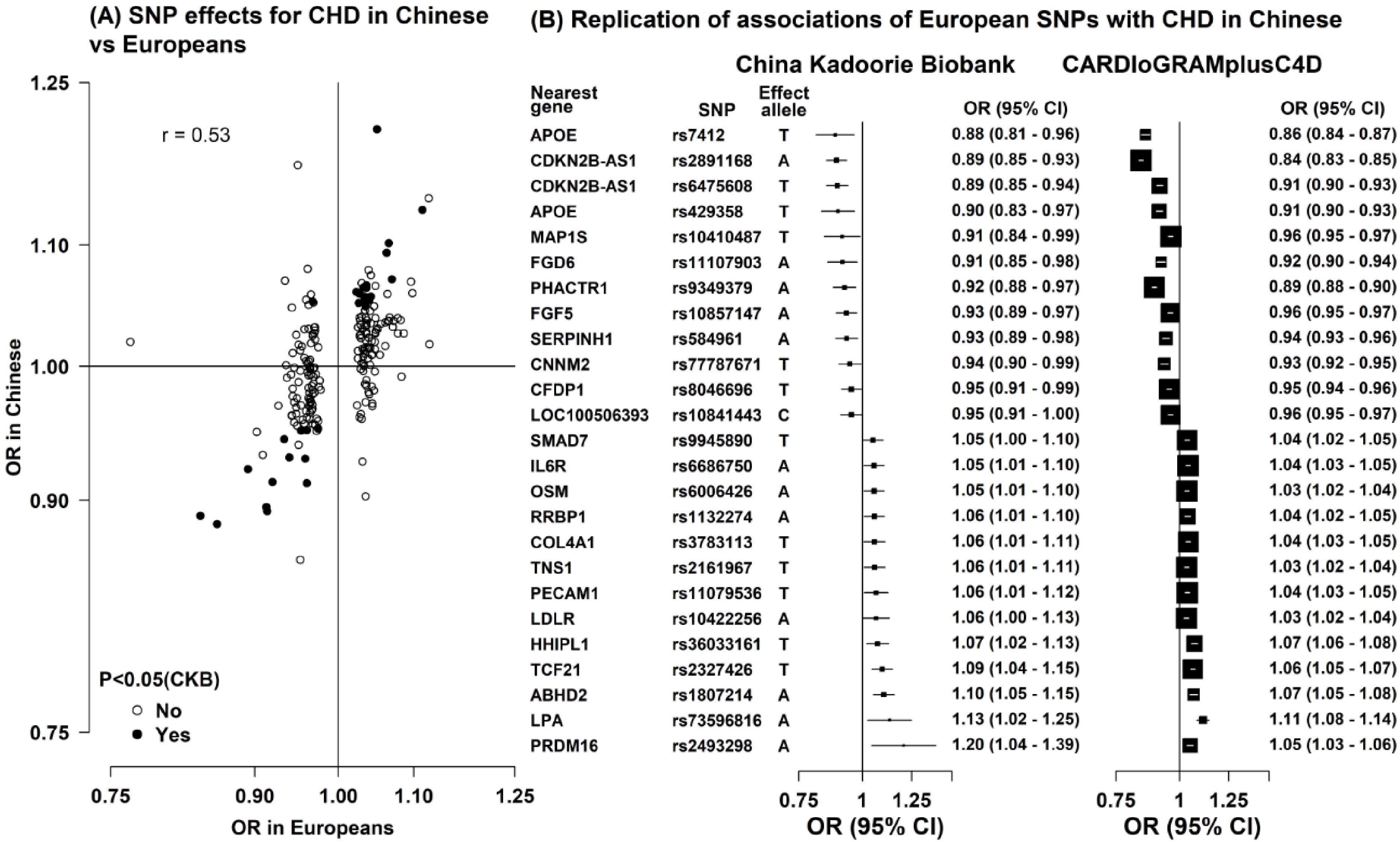

Figure 3: Odds ratios (OR) and (95%CI) of (A) major coronary events, (B) myocardial infarction or (C) chronic IHD in Chinese for fifths of a genetic score (GS-CAD) for 224 CAD SNPs discovered in Europeans.

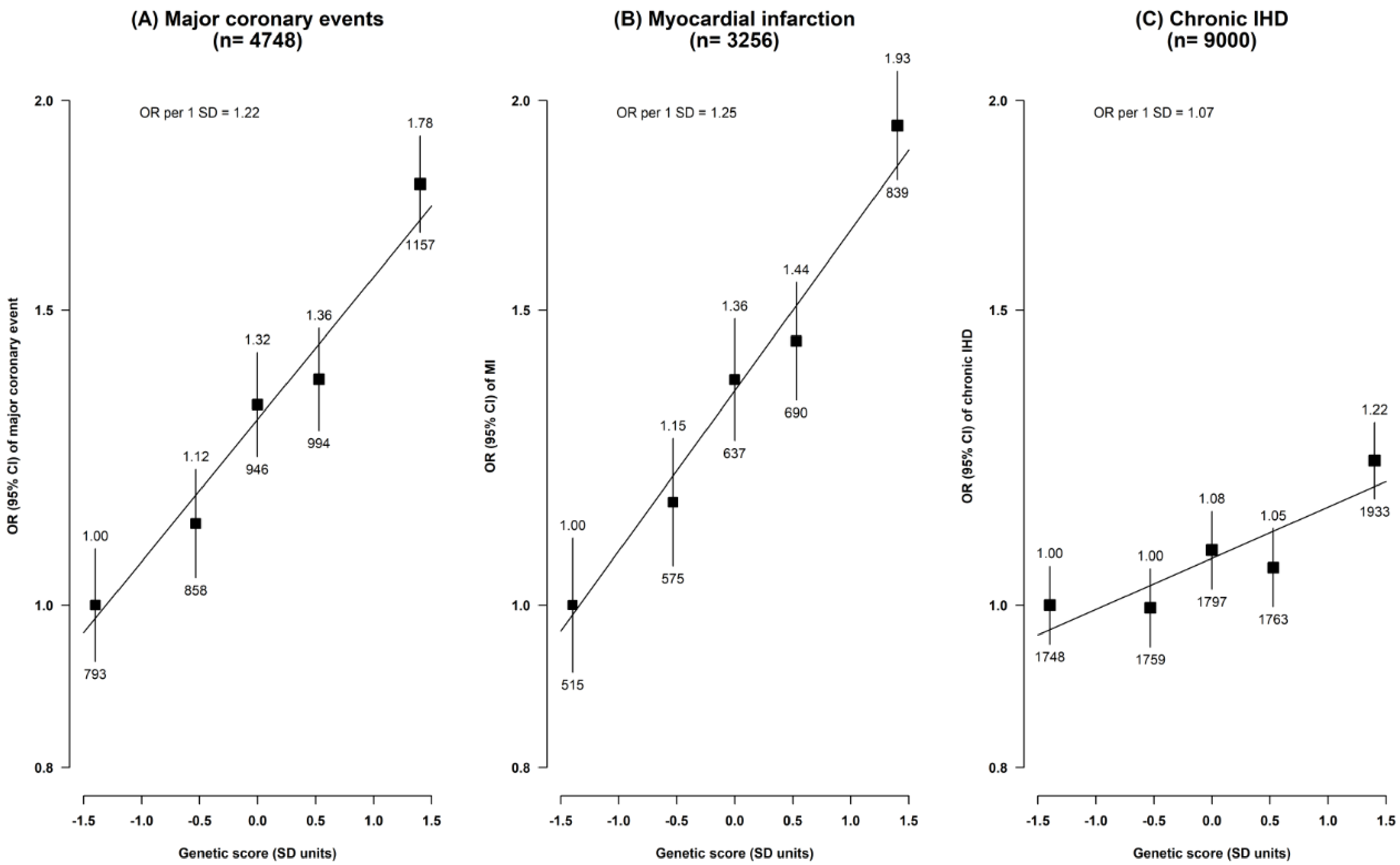

Figure 4: Odds ratios (OR) and (95% CI) of CAD, stroke and other vascular diseases in Chinese for a 1 SD higher genetic score for 224 CAD SNPs discovered in Europeans. The slope of the line represents the OR for 1 SD higher GS-CAD genetic score.

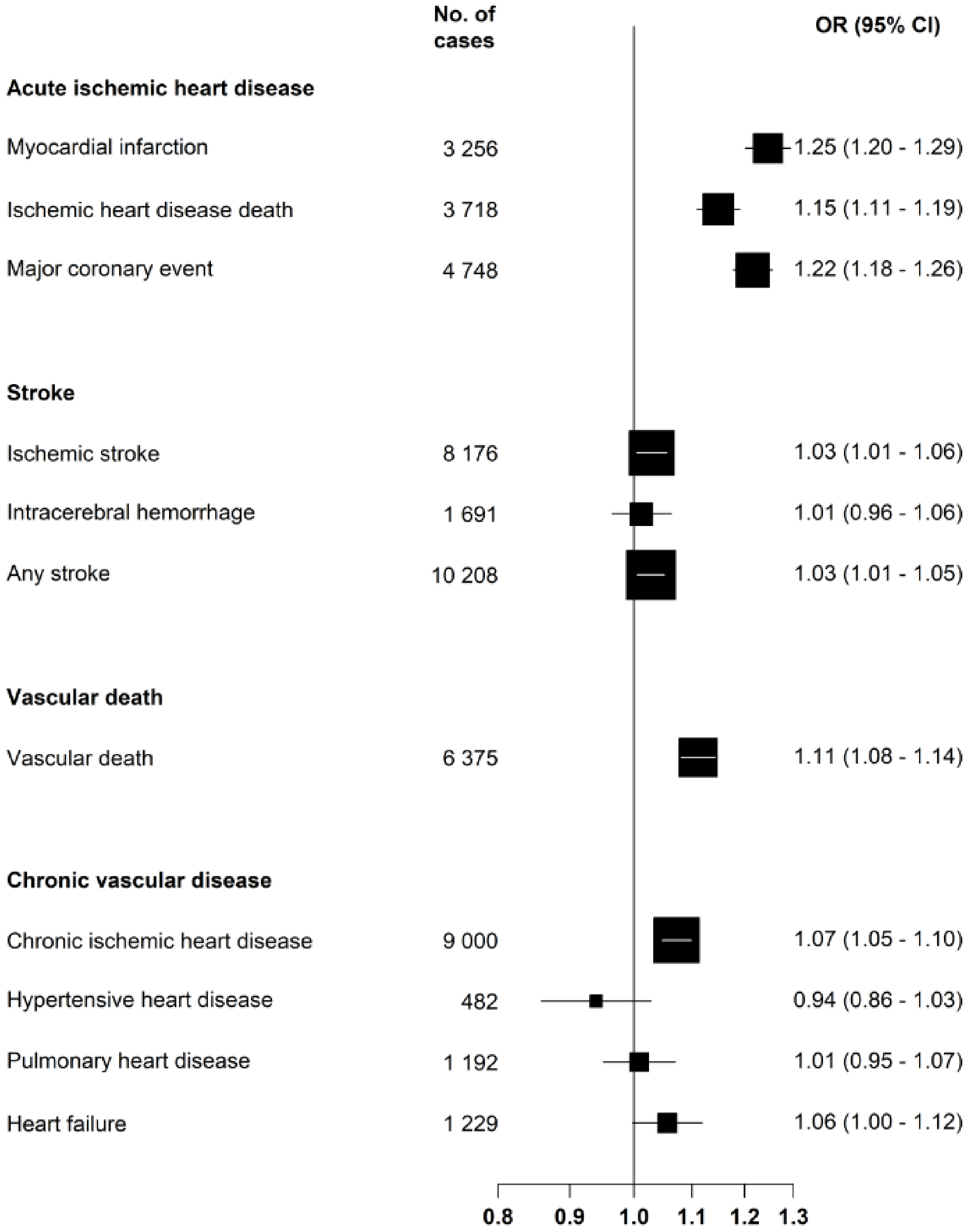

Figure 5: Associations of fifths of a European genetic score for CAD with blood pressure, body mass index and LDL-cholesterol in Chinese

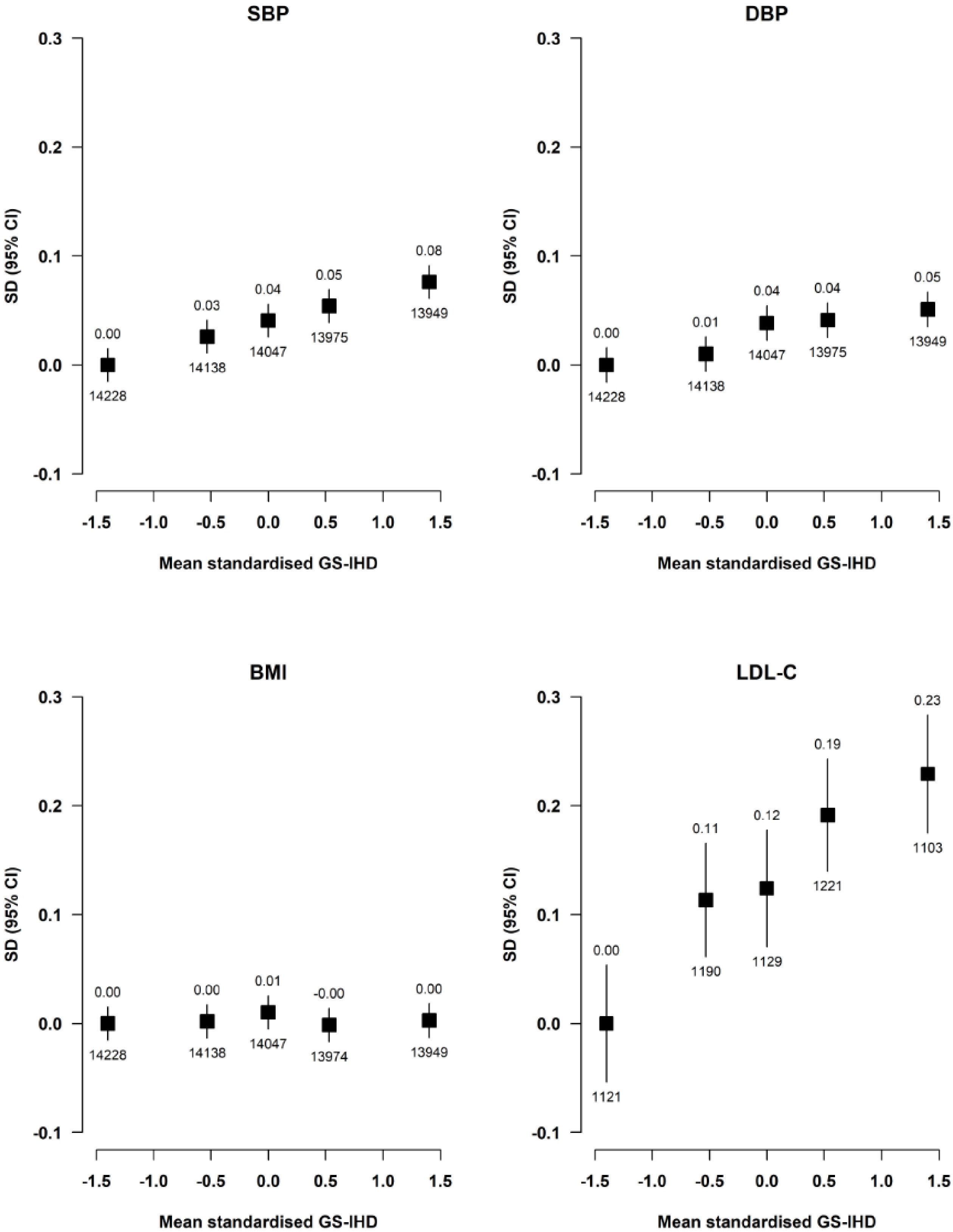

Click here to access/download
Supplementary Material
Supplementary materials for CC4D paper-16-7-2026
final.docx

Click here to access/download

Supplementary Material

Response to reviewer comments-21-4-2026.docx

Personal Cover Click here to access/download;Personal Cover;Cover letter Dr Ma -21-4-2026.docx

OXFORD POPULATION HEALTH
UNIVERSITY OF OXFORD

21 April 2026

Dr Dongi Ma
Editor
GigaScience

Re: Concordance of genetic determinants of acute coronary artery disease between European and Chinese populations

Dear Dr Ma,

We are very grateful to the Editor and reviewers for kind comments and excellent suggestions to improve our attached report for consideration for publication in *GigaScience.* The report compares the strength of associations of GWAS hits for CAD with CAD in East Asians. Myocardial infarction and acute CAD account for a smaller proportion of the Chinese population with ischaemic heart disease (IHD) than in Europeans and the relevance of these genetic variants for acute CAD discovered in Europeans for acute CAD, chronic IHD and heart failure or stroke in Chinese is uncertain.

We have outlined our responses to the reviewer comments in the attached document and uploaded a clean copy of the revised manuscript and TRACKED copy that includes all the changes made. In addition, we have added a point-by-point response to the reviewer comments in separate document. We hope that you might now consider the revised report for publication in GigaScience.

Yours sincerely

Robert Clarke
Emeritus Professor of Epidemiology and Public Health Medicine

Richard Doll Building, Old Road Campus, Roosevelt Drive, Oxford OX3 7LF
+44(0)1865 743743 enquiries@ndph.ox.ac.uk

Head of Department
Professor Sir Rory Collins FMed Sci FRCP

Graphical abstract

Click here to access/download;Graphical Abstract;Graphical abstract.docx

Concordance of genetic determinants of acute coronary artery disease between European and Chinese populations

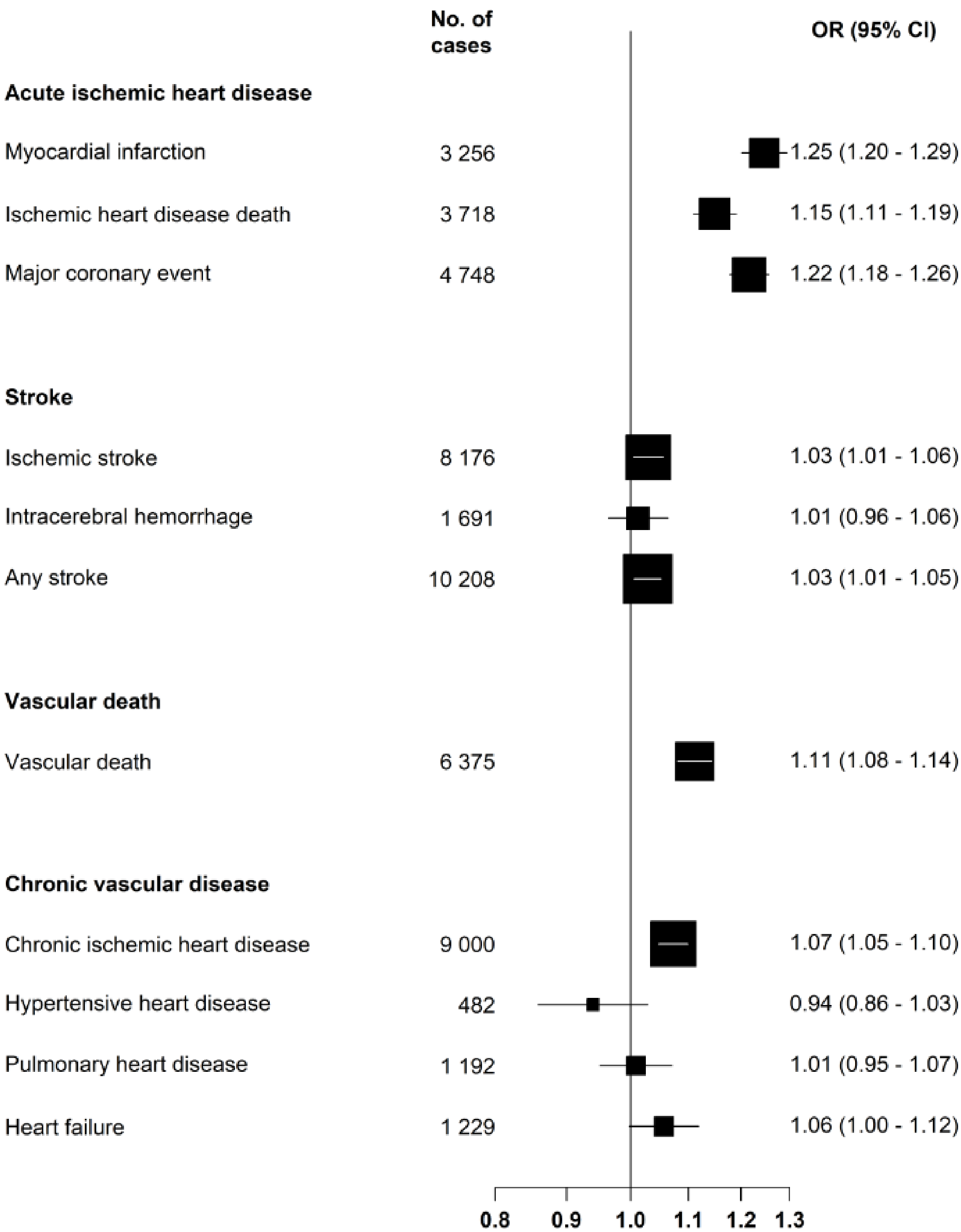

Supplement: Supplementary file 3 [file giag084_giga-d-25-00454_revision_1.pdf]
