# Supplementary material for: Concordance of genetic determinants of acute coronary artery disease between European and Chinese populations
Source: Gigascience. 2026 Aug 11;15:giag084. doi: 10.1093/gigascience/giag084 (PMC13543311; doi:10.1093/gigascience/giag084)
Supplement: Supplementary file 4 [file giag084_reviewer_1_report_original_submission.pdf]

Reviewer Report

Title: Concordance of genetic determinants of acute coronary artery disease between European and Chinese populations

Version: Original Submission **Date:** 1/4/2026

Reviewer name: Reviewer 1

Reviewer Comments to Author:

The authors reported findings on the role of SNPs discovered in the European population and and tested in the Chinese participants in the genetic determination of atherosclerotic diseases. The sample size is large and the manuscript was well written. There are several suggestions for revision of the manuscript.

1. Such studies are often in large size and may draw attention from the editors and readers. However, the authors may need to explain the clinical relevance of such studies either in the Introduction or in the Discussion, especially after so many high-cost GWAS studies of various technological platforms had been published in the past about three decades. Did these studies ever help in any sense or manner for health ?
2. Another major issue is the study design. In this study, the cases are from a large population-based study. There may be many strengths of this study design. There are apparently also limitations. A possible major limitation of such a study design is the diagnostic accuracy of the diseases under study. Thus, the authors may need to compare this kind of studies with studies based on clinical cases collected from hospitalized patients.
3. GWAS data are only available in about 20% of the total Kadoorie study participants. The authors may need to clarify why. If the selection was for purpose, the authors should also clarify.
4. There are several corresponding authors from the UK and several coauthors from China. This reviewer guesses that these Chinese authors might also contribute to this project significantly. Is it worthwhile to have a Chinese expert to be among the corresponding authors for the proper interpretation of the findings.

Methods

Are the methods appropriate to the aims of the study, are they well described, and are necessary controls included? Choose an item.

Conclusions

Are the conclusions adequately supported by the data shown? Choose an item.

Reporting Standards

Does the manuscript adhere to the journal's guidelines on [minimum standards of reporting?](#) Choose an item.

Choose an item.

Statistics

Are you able to assess all statistics in the manuscript, including the appropriateness of statistical tests used? Choose an item.

Quality of Written English

Please indicate the quality of language in the manuscript: Choose an item.

Declaration of Competing Interests

Please complete a declaration of competing interests, considering the following questions:

- Have you in the past five years received reimbursements, fees, funding, or salary from an organisation that may in any way gain or lose financially from the publication of this manuscript, either now or in the future?
- Do you hold any stocks or shares in an organisation that may in any way gain or lose financially from the publication of this manuscript, either now or in the future?
- Do you hold or are you currently applying for any patents relating to the content of the manuscript?
- Have you received reimbursements, fees, funding, or salary from an organization that holds or has applied for patents relating to the content of the manuscript?
- Do you have any other financial competing interests?
- Do you have any non-financial competing interests in relation to this paper?

If you can answer no to all of the above, write 'I declare that I have no competing interests' below. If your reply is yes to any, please give details below.

None.

I agree to the open peer review policy of the journal. I understand that my name will be included on my report to the authors and, if the manuscript is accepted for publication, my named report including any attachments I upload will be posted on the website along with the authors' responses. I agree for my report to be made available under an Open Access Creative Commons CC-BY license (http://creativecommons.org/licenses/by/4.0/). I understand that any comments which I do not wish to be included in my named report can be included as confidential comments to the editors, which will not be published.

Choose an item.

To further support our reviewers, we have joined with Publons, where you can gain additional credit to further highlight your hard work (see: https://publons.com/journal/530/gigascience). On publication of this paper, your review will be automatically added to Publons, you can then choose whether or not to claim your Publons credit. I understand this statement.

Yes Choose an item.
