# Supplementary material for: Concordance of genetic determinants of acute coronary artery disease between European and Chinese populations
Source: Gigascience. 2026 Aug 11;15:giag084. doi: 10.1093/gigascience/giag084 (PMC13543311; doi:10.1093/gigascience/giag084)
Supplement: Supplementary file 8 [file giag084_supplemental_file.docx]

**Supplementary material for “Concordance of genetic determinants of acute coronary artery disease between European and Chinese populations”**

Appendix:

[Members of the China Kadoorie Biobank Collaborative Group	2](#_Toc202937106)

[Table S1: Study endpoints and associated ICD-10 codes	3](#_Toc202937107)

[Table S2: OR (95%CI) of Major Coronary Events in CKB for variants identified in a meta-analysis of CARDIoGRAMplusC4D and Biobank Japan	4](#_Toc202937108)

[Table S3: Phenotypes associated with replicated acute IHD variants in PheWAS of CKB	12](#_Toc202937109)

[Table S4: Comparison of OR (95% CI) of major coronary events for 1 SD higher genetic score for 224 European SNPs for CAD with subset of 25 SNPs that were replicated at p<0.05 in CKB.	15](#_Toc202937110)

[Table S5: Distribution of pQTLs identified for SUSD2 and PLA2G7 proteins	16](#_Toc202937111)

[Figure S1: Distribution of SNPs from European and Japanese populations in the study	17](#_Toc202937112)

[Figure S2: Phenome-wide association of a genetic risk score for CHD with all common disease outcomes in CKB	18](#_Toc202937113)

[Figure S3: Proteome-wide analysis of GS-CHD score with 3977 proteins	19](#_Toc202937114)

[Figure S4: MR results for PLA2G7 and SUSD2 effects on MCE in CKB	20](#_Toc202937115)

# Members of the China Kadoorie Biobank Collaborative Group

**Members of the China Kadoorie Biobank Collaborative Group**

**International Steering Committee:** Junshi Chen, Zhengming Chen (PI), Robert Clarke, Rory Collins, Liming Li (PI), Chen Wang, Jun Lv, Richard Peto, Robin Walters.

**International Co-ordinating Centre, Oxford:** Daniel Avery, Derrick Bennett, Ruth Boxall, Ka Hung Chan, Yiping Chen, Zhengming Chen, Jonathan Clarke, Robert Clarke, Huaidong Du, Ahmed Edris Mohamed, Hannah Fry, Pek Kei Im, Andri Iona, Christiana Kartsonaki, Hubert Lam, Kuang Lin, James Liu, Iona Millwood, Sam Morris, Qunhua Nie, Alfred Pozarickij, Paul Ryder, Dan Schmidt, Becky Stevens, Iain Turnbull, Robin Walters, Baihan Wang, Lin Wang, Neil Wright, Ling Yang, Xiaoming Yang, Pang Yao.

**National Co-ordinating Centre, Beijing:** Xiao Han, Can Hou, Qingmei Xia, Chao Liu, Jun Lv, Pei Pei, Dianjanyi Sun, Canqing Yu

**10 Regional Co-ordinating Centres:**

**Guangxi** Provincial CDC: Naying Chen, Duo Liu, Zhenzhu Tang. Liuzhou CDC: Ningyu Chen, Qilian Jiang, Jian Lan, Mingqiang Li, Yun Liu, Fanwen Meng, Jinhuai Meng, Rong Pan, Yulu Qin, Ping Wang, Sisi Wang, Liuping Wei, Liyuan Zhou. **Gansu** Provincial CDC: Caixia Dong, Pengfei Ge, Xiaolan Ren. Maiji CDC: Zhongxiao Li, Enke Mao, Tao Wang, Hui Zhang, Xi Zhang. **Hainan** Provincial CDC: Jinyan Chen, Ximin Hu, Xiaohuan Wang. Meilan CDC: Zhendong Guo, Huimei Li, Yilei Li, Min Weng, Shukuan Wu. **Heilongjiang** Provincial CDC: Shichun Yan, Mingyuan Zou, Xue Zhou. Nangang CDC: Ziyan Guo, Quan Kang, Yanjie Li, Bo Yu, Qinai Xu. **Henan** Provincial CDC: Liang Chang, Lei Fan, Shixian Feng, Ding Zhang, Gang Zhou. Huixian CDC: Yulian Gao, Tianyou He, Pan He, Chen Hu, Huarong Sun, Xukui Zhang. **Hunan** Provincial CDC: Biyun Chen, Zhongxi Fu, Yuelong Huang, Huilin Liu, Qiaohua Xu, Li Yin. Liuyang CDC: Huajun Long, Xin Xu, Hao Zhang, Libo Zhang. **Jiangsu** Provincial CDC: Jian Su, Ran Tao, Ming Wu, Jie Yang, Jinyi Zhou, Yonglin Zhou. Suzhou CDC: Yihe Hu, Yujie Hua, Jianrong Jin, Fang Liu, Jingchao Liu, Yan Lu, Liangcai Ma, Aiyu Tang, Jun Zhang. **Qingdao** CDC: Liang Cheng, Ranran Du, Ruqin Gao, Feifei Li, Shanpeng Li, Yongmei Liu, Feng Ning, Zengchang Pang, Xiaohui Sun, Xiaocao Tian, Shaojie Wang, Yaoming Zhai, Hua Zhang, Licang CDC: Wei Hou, Silu Lv, Junzheng Wang. **Sichuan** Provincial CDC: Xiaofang Chen, Xianping Wu, Ningmei Zhang, Weiwei Zhou. Pengzhou CDC: Xiaofang Chen, Jianguo Li, Jiaqiu Liu, Guojin Luo, Qiang Sun, Xunfu Zhong. **Zhejiang** Provincial CDC: Weiwei Gong, Ruying Hu, Hao Wang,Meng Wang, Min Yu. Tongxiang CDC: Lingli Chen, Qijun Gu, Dongxia Pan，Chunmei Wang, Kaixu Xie, Xiaoyi Zhang.

# Table S1: Study end-points and associated ICD-10 codes

| **Acute vascular disease events** | ICD-10 codes |
| --- | --- |
| **Acute coronary artery disease**  Acute myocardial infarction  Death from IHD  Any acute CAD (Major coronary events) | I21 – I23  I20 – I25 (fatal)  I21 – I23 or I20 – I25 (fatal) |
| **Stroke**  Ischemic stroke  Intracerebral haemorrhage  Any stroke | I63  I61  I60 – I64 |
| **Vascular death** | I00 – I99 (fatal) R96 (fatal) |
| **Chronic IHD**  Chronic ischaemic heart disease    **Non-IHD Cardiac disease**  Hypertensive heart disease  Pulmonary heart disease  Heart failure | I25.0, I25.1, I25.3-I25.9  I10-I15  I26-I28  I50 |

# Table S2: OR (95%CI) of Major Coronary Events in CKB for variants identified in a meta-analysis of CARDIoGRAMplusC4D and Biobank Japan

| Nearest gene | rsID | Chr | Position | A1 | A2 | A1 frequency | OR | CIL | CIU | P |
| --- | --- | --- | --- | --- | --- | --- | --- | --- | --- | --- |
| CDKN2B-AS1 | rs2891168 | 9 | 22098620 | G | A | 0.456401 | 0.889246 | 0.849306 | 0.931065 | 5.55E-07 |
| CDKN2B-AS1 | rs6475608 | 9 | 22101703 | T | C | 0.346201 | 0.892426 | 0.849493 | 0.937529 | 6.06E-06 |
| ABHD2 | rs1807214 | 15 | 89022026 | C | A | 0.426177 | 1.101709 | 1.051084 | 1.154772 | 5.44E-05 |
| TCF21 | rs2327426 | 6 | 1.34E+08 | C | T | 0.427884 | 1.093496 | 1.043101 | 1.146325 | 0.000205 |
| PHACTR1 | rs9349379 | 6 | 12903725 | A | G | 0.285146 | 0.922337 | 0.876407 | 0.970674 | 0.001921 |
| FGF5 | rs10857147 | 4 | 80259918 | T | A | 0.390813 | 0.929946 | 0.887012 | 0.974958 | 0.002599 |
| APOE | rs429358 | 19 | 44908684 | C | T | 0.096051 | 0.895294 | 0.830276 | 0.965404 | 0.004036 |
| APOE | rs7412 | 19 | 44908822 | T | C | 0.082361 | 0.883461 | 0.810339 | 0.96318 | 0.004937 |
| SERPINH1 | rs584961 | 11 | 75566583 | A | G | 0.308034 | 0.93093 | 0.885171 | 0.979054 | 0.005383 |
| HHIPL1 | rs36033161 | 14 | 99657150 | C | T | 0.305618 | 1.070782 | 1.018792 | 1.125425 | 0.007077 |
| FGD6 | rs11107903 | 12 | 95114195 | A | G | 0.140983 | 0.913181 | 0.853753 | 0.976745 | 0.008161 |
| PRDM16 | rs2493298 | 1 | 3409348 | A | C | 0.022292 | 1.204873 | 1.041415 | 1.393986 | 0.012226 |
| LPA | rs73596816 | 6 | 1.61E+08 | A | G | 0.050787 | 1.130956 | 1.023934 | 1.249164 | 0.015252 |
| TRAF3IP2-AS1 | rs9400480 | 6 | 1.12E+08 | G | C | 0.465068 | 1.058876 | 1.010991 | 1.109028 | 0.015394 |
| ABCG8 | rs4245791 | 2 | 43847292 | C | T | 0.004369 | 0.677695 | 0.492546 | 0.932442 | 0.016865 |
| RRBP1 | rs1132274 | 20 | 17615510 | C | A | 0.480488 | 1.055525 | 1.008605 | 1.104627 | 0.01984 |
| TNS1 | rs2161967 | 2 | 2.18E+08 | G | T | 0.432449 | 1.056416 | 1.007951 | 1.107212 | 0.021991 |
| PECAM1 | rs11079536 | 17 | 64315043 | G | T | 0.293581 | 1.063656 | 1.008847 | 1.121443 | 0.022236 |
| OSM | rs6006426 | 22 | 30273894 | G | A | 0.460748 | 1.054164 | 1.007376 | 1.103125 | 0.02277 |
| MAP1S | rs10410487 | 19 | 17718799 | C | T | 0.08629 | 0.912238 | 0.841751 | 0.988629 | 0.025173 |
| IL6R | rs6686750 | 1 | 1.54E+08 | G | A | 0.491381 | 1.053417 | 1.006113 | 1.102945 | 0.026419 |
| COL4A1 | rs3783113 | 13 | 1.1E+08 | T | C | 0.399547 | 1.055543 | 1.006183 | 1.107325 | 0.026948 |
| SGCD | rs157333 | 5 | 1.57E+08 | G | C | 0.264316 | 1.065986 | 1.006757 | 1.1287 | 0.02846 |
| CNNM2 | rs77787671 | 10 | 1.03E+08 | T | C | 0.276526 | 0.944344 | 0.897155 | 0.994014 | 0.028557 |
| CFDP1 | rs8046696 | 16 | 75408245 | T | G | 0.472083 | 0.95077 | 0.908575 | 0.994925 | 0.02928 |
| SCUBE1 | rs139012 | 22 | 43227966 | G | A | 0.495383 | 1.052426 | 1.004923 | 1.102174 | 0.030129 |
| DOCK8 | rs1536608 | 9 | 223613 | T | G | 0.273161 | 1.060313 | 1.005094 | 1.118566 | 0.031856 |
| SNORD123 | rs17263917 | 5 | 9552226 | A | G | 0.001423 | 1.687061 | 1.025313 | 2.775907 | 0.039553 |
| LOC100506393 | rs10841443 | 12 | 20067099 | C | G | 0.41036 | 0.951108 | 0.906165 | 0.99828 | 0.042386 |
| PNPLA3 | rs738408 | 22 | 43928850 | T | C | 0.361693 | 0.952219 | 0.90797 | 0.998624 | 0.043724 |
| BCAS3 | rs11655024 | 17 | 61155004 | T | C | 0.117987 | 1.083055 | 1.002015 | 1.170649 | 0.044355 |
| GNPDA2 | rs12641981 | 4 | 45177866 | T | C | 0.297242 | 1.051026 | 1.000508 | 1.104096 | 0.047682 |
| LDLR | rs10422256 | 19 | 11105941 | A | G | 0.164051 | 1.063881 | 1.000618 | 1.131143 | 0.047731 |
| SMAD7 | rs9945890 | 18 | 48989546 | T | G | 0.380774 | 1.048449 | 1.000282 | 1.098935 | 0.048638 |
| PLG | rs186696265 | 6 | 1.61E+08 | T | C | 0.00127 | 2.126235 | 0.995052 | 4.543355 | 0.051511 |
| ARHGAP21 | rs7077962 | 10 | 24765745 | C | T | 0.148051 | 1.067669 | 0.99848 | 1.141652 | 0.05543 |
| LPA | rs10455872 | 6 | 1.61E+08 | G | A | 0.000585 | 0.252669 | 0.061479 | 1.038423 | 0.056428 |
| REST | rs781663 | 4 | 56915588 | G | A | 0.383982 | 0.955922 | 0.912339 | 1.001588 | 0.05831 |
| CFAP161 | rs2683260 | 15 | 81093211 | T | G | 0.465408 | 1.044493 | 0.997023 | 1.094222 | 0.066597 |
| DYNLRB2 | rs16952537 | 16 | 80151469 | A | G | 0.438639 | 0.958016 | 0.91465 | 1.003439 | 0.069561 |
| IRF-AS1 | rs10477741 | 5 | 1.32E+08 | G | T | 0.020136 | 0.859024 | 0.728988 | 1.012255 | 0.069594 |
| LOC400043 | rs75160195 | 12 | 54127810 | T | C | 0.164847 | 1.059284 | 0.994021 | 1.128831 | 0.075871 |
| CTAGE1 | rs9951447 | 18 | 22429728 | T | C | 0.400692 | 0.955755 | 0.908882 | 1.005045 | 0.077754 |
| GOSR2 | rs17608766 | 17 | 46935905 | C | T | 0.006608 | 0.74148 | 0.531711 | 1.034005 | 0.077915 |
| MYO9B | rs7246865 | 19 | 17108295 | A | G | 0.350869 | 1.045168 | 0.994882 | 1.097995 | 0.07908 |
| FER | rs112949822 | 5 | 1.09E+08 | A | G | 0.004817 | 1.349789 | 0.958493 | 1.900826 | 0.085926 |
| PPP2R3A | rs34330586 | 3 | 1.36E+08 | T | A | 0.118642 | 1.065335 | 0.990688 | 1.145608 | 0.087716 |
| MMP13 | rs1892971 | 11 | 1.03E+08 | A | G | 0.068606 | 1.079782 | 0.987787 | 1.180345 | 0.091119 |
| KLF4 | rs1967604 | 9 | 1.08E+08 | A | G | 0.228631 | 1.048492 | 0.992231 | 1.107942 | 0.092407 |
| MECOM | rs11721038 | 3 | 1.69E+08 | C | T | 0.15515 | 1.056351 | 0.99059 | 1.126478 | 0.094587 |
| TIMP2 | rs8075861 | 17 | 78919628 | C | A | 0.352544 | 0.959665 | 0.914377 | 1.007197 | 0.095068 |
| SCARB1 | rs7296737 | 12 | 1.25E+08 | T | C | 0.33146 | 0.959436 | 0.913698 | 1.007463 | 0.096585 |
| ITIH4 | rs77347777 | 3 | 52814191 | T | C | 0.016754 | 1.171506 | 0.970597 | 1.414002 | 0.099131 |
| EXOC3L2 | rs183657985 | 19 | 45239240 | T | C | 0.003385 | 1.339879 | 0.944274 | 1.901224 | 0.10125 |
| FLT1 | rs17086617 | 13 | 28388549 | C | T | 0.418634 | 0.960856 | 0.915595 | 1.008354 | 0.104794 |
| ZNF827 | rs13124853 | 4 | 1.46E+08 | G | A | 0.463586 | 1.039786 | 0.991801 | 1.090092 | 0.105562 |
| NGF | rs61797068 | 1 | 1.15E+08 | C | G | 0.141854 | 1.053674 | 0.9883 | 1.123372 | 0.10963 |
| TRIM5 | rs11601507 | 11 | 5679844 | A | C | 0.109048 | 1.058625 | 0.986455 | 1.136074 | 0.113776 |
| ZKSCAN1 | rs6953441 | 7 | 1E+08 | A | G | 0.378601 | 1.038839 | 0.990711 | 1.089305 | 0.115394 |
| CETP | rs12446515 | 16 | 56953103 | T | C | 0.164227 | 1.049597 | 0.987405 | 1.115707 | 0.120355 |
| SLC22A2 | rs56195640 | 6 | 1.6E+08 | A | G | 0.078952 | 1.068853 | 0.982413 | 1.162899 | 0.12172 |
| HDAC9 | rs2107595 | 7 | 19009765 | A | G | 0.326111 | 1.039236 | 0.98948 | 1.091495 | 0.124173 |
| ACER2 | rs10811183 | 9 | 19436057 | A | G | 0.133716 | 1.053421 | 0.985571 | 1.125942 | 0.125495 |
| ST3GAL4 | rs10790800 | 11 | 1.26E+08 | G | A | 0.056301 | 0.927988 | 0.843077 | 1.021451 | 0.126887 |
| LOC285696 | rs2652682 | 5 | 17113548 | T | A | 0.145298 | 0.950544 | 0.890472 | 1.014668 | 0.127807 |
| MIA3 | rs17163363 | 1 | 2.23E+08 | C | T | 0.414548 | 1.03639 | 0.989469 | 1.085536 | 0.1305 |
| MIR4432 | rs243071 | 2 | 60391893 | G | A | 0.344414 | 0.963163 | 0.917075 | 1.011567 | 0.133542 |
| NBEAL1 | rs148812085 | 2 | 2.03E+08 | T | C | 0.016729 | 1.141353 | 0.960284 | 1.356562 | 0.13357 |
| LIPA | rs1051338 | 10 | 89247603 | G | T | 0.307261 | 0.962322 | 0.914789 | 1.012326 | 0.137282 |
| FGD5 | rs34991912 | 3 | 14884844 | C | T | 0.291894 | 1.038964 | 0.987282 | 1.093352 | 0.142018 |
| PTK7 | rs1034246 | 6 | 43100632 | T | G | 0.093525 | 1.058252 | 0.98042 | 1.142264 | 0.146321 |
| MAD2L1 | rs7678555 | 4 | 1.2E+08 | C | A | 0.19372 | 0.958917 | 0.906086 | 1.014829 | 0.146813 |
| NOS3 | rs3918226 | 7 | 1.51E+08 | T | C | 0.000363 | 2.192464 | 0.749608 | 6.412546 | 0.151668 |
| B9D2 | rs1800469 | 19 | 41354391 | G | A | 0.462914 | 1.03456 | 0.987584 | 1.08377 | 0.151844 |
| HTRA1 | rs2672592 | 10 | 1.22E+08 | T | G | 0.462574 | 0.967243 | 0.923386 | 1.013183 | 0.159489 |
| DNAH10 | rs7133378 | 12 | 1.24E+08 | A | G | 0.120545 | 1.051402 | 0.980223 | 1.127749 | 0.161067 |
| FHL3 | rs61776719 | 1 | 37995647 | A | C | 0.342399 | 1.034662 | 0.986454 | 1.085227 | 0.161593 |
| VEGFA | rs6905288 | 6 | 43791136 | G | A | 0.274084 | 1.036939 | 0.985447 | 1.09112 | 0.162756 |
| ZNF259 | rs964184 | 11 | 1.17E+08 | G | C | 0.216905 | 0.961866 | 0.91061 | 1.016008 | 0.164046 |
| CELSR2 | rs12740374 | 1 | 1.09E+08 | T | G | 0.056987 | 0.93284 | 0.845322 | 1.029419 | 0.166622 |
| ANKRD13B | rs11080107 | 17 | 29611406 | C | T | 0.305864 | 0.966023 | 0.919504 | 1.014896 | 0.169818 |
| ARHGEF12 | rs4938809 | 11 | 1.2E+08 | C | T | 0.159108 | 0.955365 | 0.895067 | 1.019725 | 0.169828 |
| PLCG2 | rs7189462 | 16 | 81874262 | G | T | 0.496161 | 1.033059 | 0.985813 | 1.082569 | 0.173272 |
| SEMA3C | rs1019016 | 7 | 80941246 | T | G | 0.474968 | 1.031198 | 0.985629 | 1.078874 | 0.18277 |
| DOCK9 | rs8000794 | 13 | 98782556 | C | G | 0.201427 | 0.960783 | 0.905861 | 1.019036 | 0.182822 |
| MORF4L1 | rs7173743 | 15 | 78849442 | C | T | 0.465782 | 1.031452 | 0.985381 | 1.079677 | 0.184079 |
| SPECC1L | rs5760309 | 22 | 24281863 | T | G | 0.094453 | 0.949722 | 0.87922 | 1.025877 | 0.189919 |
| SIPA1 | rs2306363 | 11 | 65638129 | T | G | 0.248017 | 0.965508 | 0.915786 | 1.017931 | 0.193195 |
| ATXN2 | rs10774625 | 12 | 1.11E+08 | A | G | 0.003614 | 1.236419 | 0.897618 | 1.703098 | 0.193974 |
| EDNRA | rs6841581 | 4 | 1.47E+08 | A | G | 0.215482 | 1.037077 | 0.981529 | 1.09577 | 0.194903 |
| LIPC | rs588136 | 15 | 58438299 | C | T | 0.379017 | 0.969115 | 0.924032 | 1.016398 | 0.196777 |
| CXCL12 | rs494207 | 10 | 44245808 | A | G | 0.370046 | 0.969481 | 0.924739 | 1.016389 | 0.198558 |
| TMEM41B | rs4537761 | 11 | 9301806 | C | T | 0.127529 | 0.9569 | 0.894626 | 1.023509 | 0.199424 |
| NCOA6 | rs6088595 | 20 | 34770696 | A | T | 0.127942 | 1.044782 | 0.97705 | 1.117209 | 0.200171 |
| SHROOM3 | rs12500824 | 4 | 76495474 | A | G | 0.449173 | 1.029906 | 0.983424 | 1.078585 | 0.211071 |
| GUCY1A3 | rs3796587 | 4 | 1.56E+08 | G | C | 0.192888 | 1.036585 | 0.978508 | 1.09811 | 0.221919 |
| SMG6 | rs4790881 | 17 | 2165638 | A | C | 0.407517 | 1.028984 | 0.982754 | 1.077388 | 0.223131 |
| TEX41 | rs10928241 | 2 | 1.45E+08 | T | C | 0.034144 | 1.074302 | 0.956295 | 1.20687 | 0.227337 |
| ABO | rs651007 | 9 | 1.33E+08 | T | C | 0.21493 | 1.033942 | 0.978839 | 1.092148 | 0.232254 |
| COL4A1 | rs4773141 | 13 | 1.1E+08 | G | C | 0.294414 | 0.969814 | 0.921899 | 1.020219 | 0.235748 |
| NME7 | rs61806987 | 1 | 1.69E+08 | G | A | 0.018156 | 0.902889 | 0.762513 | 1.069106 | 0.23605 |
| PRDM16 | rs7413494 | 1 | 3069321 | C | G | 0.467453 | 0.971446 | 0.925465 | 1.019712 | 0.241611 |
| EIF2B2 | rs10131894 | 14 | 74980176 | C | G | 0.187342 | 0.96437 | 0.907545 | 1.024752 | 0.241641 |
| RSPH6A | rs8108474 | 19 | 45798221 | T | C | 0.252193 | 1.032372 | 0.978521 | 1.089186 | 0.243776 |
| ZEB2 | rs6740731 | 2 | 1.45E+08 | A | G | 0.358975 | 0.969207 | 0.919251 | 1.021878 | 0.246694 |
| ATP2B1 | rs2681472 | 12 | 89615182 | G | A | 0.344016 | 0.972301 | 0.927107 | 1.019699 | 0.247391 |
| ZC3HC1 | rs11556924 | 7 | 1.3E+08 | T | C | 0.051827 | 0.940274 | 0.845694 | 1.045432 | 0.254883 |
| MYH11 | rs12691049 | 16 | 15815656 | A | C | 0.261615 | 1.030927 | 0.97817 | 1.08653 | 0.255765 |
| WDR87 | rs73025613 | 19 | 37843721 | T | C | 0.235094 | 0.969766 | 0.919534 | 1.022742 | 0.25791 |
| TRIB1 | rs2001846 | 8 | 1.25E+08 | T | C | 0.433232 | 1.027279 | 0.980476 | 1.076318 | 0.257954 |
| LPA | rs1998043 | 6 | 1.61E+08 | G | A | 0.003389 | 0.803221 | 0.54918 | 1.174775 | 0.258634 |
| UHRF1BP1 | rs9469899 | 6 | 34825347 | A | G | 0.488108 | 1.025836 | 0.980361 | 1.073421 | 0.270191 |
| HDGFL1 | rs6909752 | 6 | 22612400 | A | G | 0.180957 | 1.033128 | 0.974343 | 1.095459 | 0.275542 |
| MC4R | rs476828 | 18 | 60185354 | C | T | 0.213048 | 0.970195 | 0.918652 | 1.02463 | 0.277304 |
| CPLX4 | rs11663411 | 18 | 59293278 | C | T | 0.453985 | 1.02653 | 0.979062 | 1.0763 | 0.278372 |
| NAT2 | rs4646249 | 8 | 18402921 | G | T | 0.281706 | 0.972321 | 0.924047 | 1.023118 | 0.279984 |
| FLJ12334 | rs16986953 | 2 | 19742712 | A | G | 0.346599 | 1.026324 | 0.978679 | 1.076289 | 0.284001 |
| TBX3 | rs34606058 | 12 | 1.15E+08 | C | T | 0.276152 | 1.028525 | 0.976763 | 1.08303 | 0.285716 |
| CDH13 | rs7500448 | 16 | 83012185 | G | A | 0.239595 | 1.030102 | 0.97522 | 1.088073 | 0.288362 |
| PLCE1 | rs55753709 | 10 | 94269413 | C | T | 0.400678 | 0.975347 | 0.930817 | 1.022006 | 0.295097 |
| MACC1 | rs10486389 | 7 | 20260793 | A | G | 0.272166 | 0.972508 | 0.922729 | 1.024973 | 0.298393 |
| ITGA1 | rs4074793 | 5 | 52897294 | G | A | 0.068153 | 0.954426 | 0.873802 | 1.04249 | 0.300255 |
| COL4A2 | rs9515203 | 13 | 1.1E+08 | C | T | 0.128637 | 1.036042 | 0.968717 | 1.108045 | 0.301664 |
| CDC123 | rs17566555 | 10 | 12233948 | A | G | 0.309884 | 1.025763 | 0.976634 | 1.077364 | 0.309725 |
| ELL | rs78030362 | 19 | 18464383 | G | A | 0.005671 | 0.78733 | 0.495885 | 1.250064 | 0.310709 |
| MCF2L-AS1 | rs4907571 | 13 | 1.13E+08 | C | T | 0.218028 | 0.971254 | 0.917603 | 1.028043 | 0.314396 |
| ACVR2A | rs35611688 | 2 | 1.48E+08 | C | T | 0.176324 | 1.031166 | 0.971159 | 1.094881 | 0.315723 |
| GP2 | rs10852238 | 16 | 20242052 | T | A | 0.291611 | 1.027487 | 0.97434 | 1.083534 | 0.316972 |
| ADAMTS7 | rs7177201 | 15 | 78773038 | C | T | 0.111431 | 1.038661 | 0.964153 | 1.118927 | 0.317902 |
| UMPS | rs17843797 | 3 | 1.25E+08 | G | T | 0.074537 | 1.047301 | 0.956065 | 1.147244 | 0.3203 |
| MMP24 | rs17406518 | 20 | 35211373 | C | A | 0.10771 | 1.038716 | 0.963645 | 1.119635 | 0.320976 |
| MAD1L1 | rs62435159 | 7 | 1871645 | A | G | 0.187999 | 1.030006 | 0.971265 | 1.092298 | 0.323731 |
| CMIP | rs10493891 | 16 | 81477137 | T | C | 0.352179 | 1.024305 | 0.976487 | 1.074465 | 0.324865 |
| HBS1L | rs9399136 | 6 | 1.35E+08 | C | T | 0.275315 | 1.025375 | 0.973774 | 1.079711 | 0.341502 |
| TARID | rs6919211 | 6 | 1.34E+08 | G | C | 0.221544 | 0.974445 | 0.923084 | 1.028664 | 0.348736 |
| BSND | rs34232196 | 1 | 55023869 | T | C | 0.138621 | 0.968332 | 0.905272 | 1.035785 | 0.348944 |
| LOC283033 | rs1870634 | 10 | 43985363 | T | G | 0.269811 | 0.975307 | 0.925136 | 1.0282 | 0.353451 |
| AFAP1L2 | rs646668 | 10 | 1.14E+08 | A | G | 0.304148 | 1.024358 | 0.972642 | 1.078823 | 0.362554 |
| KIAA0040 | rs2285219 | 1 | 1.75E+08 | T | A | 0.103106 | 1.036638 | 0.95919 | 1.120339 | 0.363733 |
| CABIN1 | rs12484557 | 22 | 24159893 | G | A | 0.098627 | 1.035213 | 0.96016 | 1.116133 | 0.367454 |
| KDF1 | rs79598313 | 1 | 26958422 | T | C | 0.000293 | 2.137159 | 0.397594 | 11.48773 | 0.376099 |
| FAM46A | rs9361867 | 6 | 81886242 | T | C | 0.172766 | 0.972669 | 0.913883 | 1.035236 | 0.383612 |
| CFTR | rs13222797 | 7 | 1.17E+08 | G | T | 0.473209 | 0.97951 | 0.934191 | 1.027026 | 0.391659 |
| COL4A1 | rs11619113 | 13 | 1.1E+08 | G | C | 0.02113 | 1.069678 | 0.914787 | 1.250794 | 0.398665 |
| PRKCE | rs582384 | 2 | 45669298 | A | C | 0.250311 | 1.022598 | 0.970503 | 1.07749 | 0.402223 |
| WT1 | rs7118294 | 11 | 32358975 | T | C | 0.26734 | 0.978366 | 0.929554 | 1.029741 | 0.402251 |
| MCL1 | rs11585169 | 1 | 1.51E+08 | A | T | 0.380214 | 0.979994 | 0.93444 | 1.02777 | 0.405345 |
| HHAT | rs60154123 | 1 | 2.1E+08 | T | C | 0.296529 | 0.978354 | 0.929078 | 1.030243 | 0.406555 |
| PCSK9 | rs472495 | 1 | 55055640 | G | T | 0.2587 | 1.022865 | 0.969248 | 1.079449 | 0.410521 |
| ACVRL1 | rs2277383 | 12 | 51920604 | G | T | 0.321544 | 0.979484 | 0.931969 | 1.02942 | 0.413875 |
| LOC646736 | rs952227 | 2 | 2.26E+08 | A | G | 0.110361 | 0.96707 | 0.891329 | 1.049248 | 0.420997 |
| MTAP | rs11523031 | 9 | 21843843 | G | A | 0.474485 | 0.98118 | 0.936633 | 1.027846 | 0.422873 |
| CDKN2B-AS1 | rs76959412 | 9 | 22011084 | C | G | 0.000193 | 0.047841 | 2.27E-05 | 100.6659 | 0.436173 |
| LIPG | rs12965923 | 18 | 49687312 | A | G | 0.004407 | 0.858603 | 0.584715 | 1.260784 | 0.436714 |
| IGF2BP1 | rs4643373 | 17 | 49046061 | C | T | 0.013019 | 1.07872 | 0.882763 | 1.318176 | 0.458788 |
| PDE1A | rs1430158 | 2 | 1.82E+08 | T | C | 0.347868 | 0.981642 | 0.934559 | 1.031098 | 0.46001 |
| NA | rs75655731 | 8 | 9863884 | C | G | 0.104398 | 0.972687 | 0.901332 | 1.049692 | 0.476215 |
| COG5 | rs12112877 | 7 | 1.07E+08 | C | T | 0.292056 | 1.018414 | 0.968018 | 1.071433 | 0.481013 |
| ZNF335 | rs8124182 | 20 | 45980262 | A | G | 0.097928 | 1.026238 | 0.952888 | 1.105235 | 0.493631 |
| KAT2A | rs12952244 | 17 | 42118063 | G | A | 0.12495 | 1.025212 | 0.953707 | 1.102079 | 0.499661 |
| NCRNA00310 | rs149487184 | 21 | 34233562 | T | C | 0.000125 | 0.012393 | 3.33E-08 | 4613.001 | 0.502291 |
| TCF7L2 | rs7903146 | 10 | 1.13E+08 | T | C | 0.035336 | 0.959592 | 0.850363 | 1.082851 | 0.503496 |
| PIP4K2B | rs16968377 | 17 | 38786143 | C | T | 0.044321 | 0.963459 | 0.861898 | 1.076987 | 0.512468 |
| SYN3 | rs4452 | 22 | 32887270 | T | C | 0.08885 | 1.029692 | 0.942772 | 1.124626 | 0.515506 |
| OBFC1 | rs2067831 | 10 | 1.04E+08 | C | G | 0.175089 | 1.019936 | 0.960559 | 1.082983 | 0.518902 |
| COL4A2 | rs7333991 | 13 | 1.1E+08 | C | T | 0.266893 | 1.017294 | 0.964731 | 1.072721 | 0.526424 |
| GPSM1 | rs3935875 | 9 | 1.36E+08 | A | G | 0.028198 | 0.954719 | 0.826702 | 1.102559 | 0.528146 |
| SKI | rs2843152 | 1 | 2314131 | C | G | 0.367055 | 0.984356 | 0.937149 | 1.033941 | 0.529454 |
| CCDC30 | rs6656344 | 1 | 42482914 | C | A | 0.424113 | 1.015108 | 0.968634 | 1.063813 | 0.530564 |
| WIPI1 | rs2909217 | 17 | 68467844 | C | T | 0.46195 | 0.984965 | 0.939414 | 1.032726 | 0.530617 |
| PALLD | rs869396 | 4 | 1.69E+08 | C | A | 0.456715 | 0.984731 | 0.938302 | 1.033458 | 0.532353 |
| APOB | rs515135 | 2 | 21063185 | T | C | 0.102172 | 0.976771 | 0.907075 | 1.051823 | 0.533763 |
| CDK8 | rs12864131 | 13 | 26471802 | A | G | 0.298411 | 0.983996 | 0.934568 | 1.036038 | 0.539497 |
| NCRNA00310 | rs28451064 | 21 | 34221526 | A | G | 0.001209 | 1.260899 | 0.597507 | 2.660834 | 0.542909 |
| ARHGAP42 | rs633185 | 11 | 1.01E+08 | C | G | 0.486589 | 1.014469 | 0.968453 | 1.062671 | 0.544154 |
| PPAP2B | rs11206803 | 1 | 56411837 | T | C | 0.450298 | 1.014508 | 0.967903 | 1.063357 | 0.548293 |
| PID1 | rs4140748 | 2 | 2.29E+08 | G | A | 0.450501 | 1.013944 | 0.967724 | 1.062372 | 0.560747 |
| EDNRA | rs13120678 | 4 | 1.47E+08 | A | G | 0.49216 | 1.01368 | 0.967563 | 1.061995 | 0.567368 |
| ARVCF | rs71313931 | 22 | 19972661 | G | C | 0.147106 | 0.981705 | 0.920804 | 1.046635 | 0.572026 |
| MRAS | rs185244 | 3 | 1.38E+08 | T | C | 0.2236 | 1.016034 | 0.960726 | 1.074527 | 0.577517 |
| ANGPTL4 | rs116843064 | 19 | 8364439 | A | G | 0.000227 | 0.256554 | 0.001985 | 33.15657 | 0.583377 |
| LPL | rs894211 | 8 | 20008236 | T | C | 0.192829 | 1.015837 | 0.958838 | 1.076224 | 0.593808 |
| TBX20 | rs2215614 | 7 | 35237481 | C | A | 0.236442 | 1.014622 | 0.961847 | 1.070292 | 0.594288 |
| ZHX3 | rs6102343 | 20 | 41295639 | A | G | 0.280204 | 0.986218 | 0.937021 | 1.037999 | 0.595046 |
| OTUD7B | rs67807996 | 1 | 1.5E+08 | A | G | 0.277569 | 1.014406 | 0.961961 | 1.069711 | 0.597421 |
| MAP3K1 | rs3936510 | 5 | 56565039 | T | G | 0.117588 | 1.019428 | 0.949014 | 1.095066 | 0.598252 |
| LOC339894 | rs4266144 | 3 | 1.57E+08 | C | G | 0.351513 | 0.986486 | 0.937561 | 1.037965 | 0.600106 |
| SLC22A1 | rs6932293 | 6 | 1.6E+08 | C | T | 0.117061 | 1.019532 | 0.947675 | 1.096837 | 0.60394 |
| XPO6 | rs111806192 | 16 | 28241061 | T | G | 0.095558 | 1.021488 | 0.942531 | 1.107058 | 0.604469 |
| FES | rs7183988 | 15 | 90885359 | T | G | 0.20595 | 1.016717 | 0.954161 | 1.083375 | 0.608854 |
| ATP1B3 | rs7622417 | 3 | 1.42E+08 | G | C | 0.455172 | 1.012306 | 0.965732 | 1.061125 | 0.610783 |
| AAK1 | rs12468870 | 2 | 69452405 | C | G | 0.488615 | 0.98884 | 0.944569 | 1.035186 | 0.631055 |
| ANKRD55 | rs62362364 | 5 | 56145744 | C | G | 0.13286 | 0.983227 | 0.915836 | 1.055576 | 0.640535 |
| PREX1 | rs2008614 | 20 | 48816613 | C | T | 0.032421 | 0.971175 | 0.852434 | 1.106457 | 0.660237 |
| PARTICL | rs10176176 | 2 | 85534925 | T | A | 0.377705 | 1.010575 | 0.96389 | 1.059521 | 0.662885 |
| ADAMTS7 | rs112238647 | 15 | 78759363 | T | C | 0.000854 | 0.77411 | 0.226118 | 2.650148 | 0.683433 |
| PCSK9 | rs11591147 | 1 | 55039974 | T | G | 5.59E-05 | 252.5605 | 5.02E-10 | 1.27E+14 | 0.687392 |
| VPS11 | rs1177562 | 11 | 1.19E+08 | T | C | 0.180199 | 1.012392 | 0.953367 | 1.075071 | 0.68781 |
| GIGYF2 | rs283485 | 2 | 2.33E+08 | A | G | 0.307808 | 1.00996 | 0.961089 | 1.061317 | 0.695323 |
| PRIM2 | rs11752218 | 6 | 57280764 | T | C | 0.02995 | 1.026224 | 0.896342 | 1.174926 | 0.707703 |
| SAP130 | rs114192718 | 2 | 1.28E+08 | T | C | 3.79E-05 | 0.016668 | 7.96E-12 | 34892124 | 0.708475 |
| MIR4693 | rs2839812 | 11 | 1.04E+08 | A | T | 0.372224 | 0.991071 | 0.94445 | 1.039994 | 0.715239 |
| FOXC1 | rs72836800 | 6 | 1617092 | C | T | 0.002062 | 0.90554 | 0.520623 | 1.575041 | 0.725319 |
| SLC2A12 | rs2492304 | 6 | 1.34E+08 | A | T | 0.204936 | 0.989319 | 0.931025 | 1.051264 | 0.728923 |
| RAC1 | rs10951983 | 7 | 6406396 | G | A | 0.078758 | 1.015032 | 0.931249 | 1.106353 | 0.734265 |
| COL4A1 | rs11617955 | 13 | 1.1E+08 | A | T | 0.004584 | 1.066814 | 0.728431 | 1.562389 | 0.739701 |
| PLEKHG1 | rs17080093 | 6 | 1.51E+08 | T | C | 0.107511 | 0.987708 | 0.917681 | 1.063078 | 0.741657 |
| PTRF | rs8068844 | 17 | 42419266 | C | T | 0.31309 | 0.992041 | 0.944256 | 1.042244 | 0.751049 |
| DOCK5 | rs1510758 | 8 | 25204291 | G | A | 0.428884 | 1.007604 | 0.960854 | 1.056629 | 0.75464 |
| SWAP70 | rs360153 | 11 | 9740727 | C | T | 0.408046 | 0.993004 | 0.947812 | 1.040351 | 0.767684 |
| TSPAN14 | rs17680741 | 10 | 80491758 | C | T | 0.025631 | 1.020743 | 0.886998 | 1.174654 | 0.77448 |
| LDLR | rs72658867 | 19 | 11120527 | A | G | 9.99E-05 | 5.280489 | 3.25E-05 | 859115.7 | 0.785777 |
| MAP9 | rs7440763 | 4 | 1.56E+08 | T | G | 0.452021 | 0.993581 | 0.948342 | 1.040978 | 0.786509 |
| HMGCR | rs12916 | 5 | 75360714 | T | C | 0.471971 | 0.993853 | 0.949768 | 1.039983 | 0.789948 |
| JCAD | rs9337951 | 10 | 30028144 | A | G | 0.024577 | 1.024253 | 0.856501 | 1.22486 | 0.792865 |
| KCNK5 | rs733701 | 6 | 39204086 | T | C | 0.116673 | 1.009375 | 0.939609 | 1.08432 | 0.798449 |
| PDE1C | rs215634 | 7 | 32329536 | G | A | 0.366611 | 1.006226 | 0.958736 | 1.056068 | 0.801338 |
| GEM | rs34917849 | 8 | 94266079 | C | G | 0.122241 | 1.008939 | 0.940447 | 1.082419 | 0.804053 |
| PPAP2B | rs56170783 | 1 | 56550459 | C | A | 0.026836 | 1.01751 | 0.87858 | 1.178409 | 0.816732 |
| ZNF831 | rs6026739 | 20 | 59164414 | T | A | 0.045393 | 1.012818 | 0.905642 | 1.132676 | 0.823389 |
| DMXL1 | rs13169691 | 5 | 1.19E+08 | T | C | 0.005715 | 0.963927 | 0.694292 | 1.338277 | 0.826291 |
| BMP1 | rs56408342 | 8 | 22190977 | A | G | 0.001751 | 1.066935 | 0.595625 | 1.911185 | 0.827552 |
| SMAD3 | rs56062135 | 15 | 67163292 | T | C | 0.022354 | 0.982564 | 0.838555 | 1.151305 | 0.827794 |
| MICA | rs9266631 | 6 | 31379121 | A | G | 0.157696 | 1.006794 | 0.947025 | 1.070334 | 0.828336 |
| FN1 | rs1250247 | 2 | 2.15E+08 | C | G | 0.088916 | 0.98978 | 0.898874 | 1.08988 | 0.834454 |
| LAMB2 | rs34759087 | 3 | 49124851 | T | C | 0.002776 | 1.038974 | 0.720229 | 1.498781 | 0.837953 |
| SRFBP1 | rs4345341 | 5 | 1.22E+08 | A | T | 0.061745 | 0.987997 | 0.878644 | 1.11096 | 0.840097 |
| LOC100131060 | rs71646019 | 1 | 58967682 | T | C | 0.142556 | 1.006516 | 0.943059 | 1.074242 | 0.845022 |
| FBN2 | rs6883598 | 5 | 1.29E+08 | C | A | 0.136832 | 0.993451 | 0.928634 | 1.062792 | 0.848632 |
| CXCR4 | rs4954580 | 2 | 1.36E+08 | T | C | 0.422033 | 0.995632 | 0.949427 | 1.044085 | 0.856711 |
| C9orf146 | rs10961206 | 9 | 13724052 | T | A | 0.052717 | 1.009049 | 0.910463 | 1.118309 | 0.863647 |
| FDX1 | rs10488763 | 11 | 1.1E+08 | T | A | 0.329717 | 1.004091 | 0.955176 | 1.05551 | 0.872716 |
| SERPINA1 | rs112635299 | 14 | 94371805 | T | G | 1.82E-05 | 0.000131 | 1.34E-52 | 1.27E+44 | 0.873954 |
| ARHGAP15 | rs4662330 | 2 | 1.43E+08 | C | T | 0.304349 | 0.99597 | 0.947378 | 1.047054 | 0.87427 |
| FGF1 | rs249760 | 5 | 1.43E+08 | C | T | 0.478624 | 0.996281 | 0.951283 | 1.043408 | 0.874463 |
| DAB2IP | rs885150 | 9 | 1.22E+08 | C | T | 0.290576 | 1.003988 | 0.95344 | 1.057215 | 0.879981 |
| HNF1A | rs2244608 | 12 | 1.21E+08 | G | A | 0.408617 | 0.996482 | 0.951627 | 1.043451 | 0.880782 |
| CCM2 | rs2107732 | 7 | 45038379 | A | G | 0.00664 | 0.969418 | 0.611061 | 1.537933 | 0.895054 |
| SREBF1 | rs12936927 | 17 | 17823651 | T | C | 0.06856 | 1.005148 | 0.918407 | 1.100081 | 0.911215 |
| R3HCC1L | rs884811 | 10 | 98164006 | G | C | 0.455368 | 1.002613 | 0.956868 | 1.050545 | 0.912792 |
| ABCG8 | rs76866386 | 2 | 43848344 | C | T | 0.017555 | 0.991965 | 0.841323 | 1.16958 | 0.923524 |
| PAWR | rs8176893 | 12 | 79605529 | A | T | 0.383954 | 0.99773 | 0.949725 | 1.048162 | 0.928027 |
| LDLR | rs55997232 | 19 | 11077441 | T | C | 0.008448 | 0.9891 | 0.756557 | 1.29312 | 0.936118 |
| TFAP2B | rs62405422 | 6 | 50829192 | C | T | 0.228609 | 0.997891 | 0.945339 | 1.053365 | 0.939031 |
| ARHGAP26 | rs3776307 | 5 | 1.43E+08 | G | A | 0.14506 | 0.997677 | 0.934205 | 1.065461 | 0.944708 |
| SLC18A1 | rs66778572 | 8 | 20058609 | A | G | 0.255666 | 1.001876 | 0.94997 | 1.056618 | 0.944947 |
| N4BP2L2 | rs7991314 | 13 | 32551937 | C | T | 0.165797 | 0.997971 | 0.939179 | 1.060442 | 0.947716 |
| UNC13D | rs2410859 | 17 | 75845204 | C | T | 0.444598 | 1.001496 | 0.955641 | 1.04955 | 0.950161 |
| PLG | rs184278183 | 6 | 1.61E+08 | T | C | 0.000113 | 1.204024 | 0.003278 | 442.2633 | 0.95087 |
| FIGN | rs10930115 | 2 | 1.64E+08 | T | A | 0.407481 | 1.00112 | 0.954985 | 1.049484 | 0.962901 |
| SCARB1 | rs7485656 | 12 | 1.25E+08 | A | G | 0.409584 | 1.000986 | 0.954415 | 1.049829 | 0.967664 |
| TGFB1 | rs11466359 | 19 | 41331710 | A | G | 0.278438 | 0.99913 | 0.948164 | 1.052836 | 0.974009 |
| LNX1 | rs17083333 | 4 | 53705899 | T | G | 0.455434 | 0.9992 | 0.951778 | 1.048986 | 0.974278 |
| DHX38 | rs12445401 | 16 | 72114520 | G | A | 0.243099 | 0.999295 | 0.946881 | 1.054611 | 0.979533 |
| FHL5 | rs2983896 | 6 | 96581995 | A | G | 0.067878 | 0.999222 | 0.91238 | 1.09433 | 0.986615 |
| RGL3 | rs167479 | 19 | 11416089 | T | G | 0.479607 | 0.999744 | 0.954862 | 1.046735 | 0.991275 |
| LOC145820 | rs17581137 | 15 | 95603185 | C | A | 0.103987 | 1.000129 | 0.925254 | 1.081063 | 0.99741 |

# Table S3: Phenotypes associated with replicated acute IHD variants in PheWAS of CKB

| **rsID** | **Gene name** | **PheWAS of major**  **diseases in UKB** | **PheWAS of**  **continuous**  **traits in CKB** |
| --- | --- | --- | --- |
| rs2493298 | PRDM16 | Hypertension (+) | DBP(+)  SBP(+) |
| rs6686750 | IL6R |  | Albumin(-)  Alkaline phosphatase(+)  C-reactive-protein(+)  SHBG(-) |
| rs2161967 | TNS1 |  | DBP(+)  SBP(+) |
| rs10857147 | FGF5 | Hypercholesterolemia (+)   Hypertension (+) | Apolipoprotein-B(-)  Cholesterol(-)  Creatinine(-)  Cystatin-C(-)  DBP(+)  SBP(+)  Urate (-) |
| rs9349379 | PHACTR1 | Migraine (-) | SBP(-) |
| rs2327426 | TCF21 | Hypertension (+) | Creatinine(-)  Urea(-) |
| rs73596816 | LPA | Hypercholesterolemia (+) | Apolipoprotein-B(+)  Cholesterol(+)  LDL-direct(+)  Lipoprotein A(+) |
| rs2891168 | CDKN2B-AS1 | Type 2 Diabetes (+)  Glaucoma (+)  Heart Failure (+)  Hypercholesterolemia (+)  Peripheral vascular disease (+) | Apolipoprotein-B(-)  Creatinine (+) |
| rs6475608 | CDKN2B-AS1 |  |  |
| rs77787671 | CNNM2 | Hypertension (+) | BMI(-)  DBP(+)  HbA1c(+)  SBP(+) |
| rs584961 | SERPINA1 |  |  |
| rs10841443 | LOC100506393 | Hypertension (+) | DBP(+) |
| rs11107903 | FGD6 |  |  |
| rs3783113 | COL4A1 |  | DBP(+) |
| rs36033161 | HHIPL1 |  |  |
| rs1807214 | ABHD2 |  | Creatinine(+)  Glucose(+)  Oestradiol (+) |
| rs8046696 | CFDP1 | Hypertension (+) | DBP(+)  SBP(+) |
| rs11079536 | PECAM1 |  | SBP(+) |
| rs9945890 | SMAD7 | Hypercholesterolemia (+) |  |
| rs10422256 | LDLR | Hypercholesterolemia (+) | Apolipoprotein-B(+)  Cholesterol(+)  HbA1c(+)  LDL-direct (+) |
| rs10410487 | MAP1S |  | Gamma glutamyltransferase (+) |
| rs429358 | APOE | Alzheimers or Dementia (+)  Hypercholesterolemia (+) | Alanine aminotransferase (-)  Alkaline-phosphatase(-)  Apolipoprotein-A(-)  Apolipoprotein-B(+)  BMI(-)  C-reactive-protein(-)  Cholesterol(+)  Creatinine(-)  Cystatin-C(-)  Gamma glutamyltransferase(-)  HDL-cholesterol(-)  LDL-direct(+)  SHBG(+)  Triglycerides(+)  Urate(-)  Urea (-) |
| rs7412 | APOE | Hypercholesterolemia (+)   Hypertension (+) | Albumin(+)  Alkaline-phosphatase(-)  Apolipoprotein-A(-)  Apolipoprotein-B(+)  BMI(-)  C-reactive-protein(-)  Cholesterol(+)  Creatinine(-)  Cystatin-C(-)  Direct-bilirubin(-)  HDL-cholesterol(-)  LDL-direct(+)  Lipoprotein(a)(+)  SBP(+)  Triglycerides(-) |
| rs1132274 | RRBP1 |  | Alkaline-phosphatase(+)  Apolipoprotein-A(-)  Aspartate aminotransferase(-)  Vitamin-D(+) |

| **No. of cases** | **No. of SNPs** | **OR (95% CI) of major coronary events** |
| --- | --- | --- |
| 4748  4748 | 224  25 | 1.22 (1.18 – 1.26)  1.21 (1.17 – 1.25) |

# Table S5: Distribution of pQTLs identified for SUSD2 and PLA2G7 proteins

| rsID | Chr | Position | Effect allele | Non-effect allele | Protein |
| --- | --- | --- | --- | --- | --- |
| rs76863441 | 6 | 46709361 | A | C | PLA2G7 |
| rs145598072 | 22 | 24250747 | T | C | SUSD2 |

# Figure S1: Distribution of SNPs from European and Japanese populations in the study

# Figure S2: Phenome-wide association of a genetic risk score for CHD with all common disease outcomes in CKB

**The X-axis shows the different phenotype categories and Y-axis represents the log(p-values). The data point shapes represent the direction of effects and the point sizes represent the effect sizes. The horizontal red line denotes the Bonferroni significant threshold for CHD used in CKB.**

# Figure S3: Proteome-wide analysis of GS-CHD scores with 3977 proteins

**The upper panel (A) shows the associations using the full GS and the lower panel (B) shows the results using an adjusted genetic score after removal of any variants within a 1 Mb of the gene region to which the protein was annotated.**

# Figure S4: MR results for PLA2G7 and SUSD2 effects on MCE in CKB
